# Lysine Phoshoglycerylation Is Widespread in Bacteria and Overlaps with Acylation

**DOI:** 10.3390/microorganisms12081556

**Published:** 2024-07-30

**Authors:** Stefan Mikkat, Michael Kreutzer, Nadja Patenge

**Affiliations:** 1 Core Facility Proteome Analysis, Rostock University Medical Center, 18057 Rostock, Germany; 2 Medical Research Center, Rostock University Medical Center, 18057 Rostock, Germany; michael.kreutzer@med.uni-rostock.de; 3 Institute of Medical Microbiology, Virology and Hygiene, Rostock University Medical Center, 18057 Rostock, Germany; nadja.patenge@med.uni-rostock.de

**Keywords:** *Streptococcus pyogenes*, phosphoglycerylation, acetylation, phosphoglyceryl-lysine, post-translational modification, immonium and diagnostic ions, proteomics

## Abstract

Phosphoglycerylation is a non-enzymatic protein modification in which a phosphoglyceryl moiety is covalently bound to the ε-amino group of lysine. It is enriched in glycolytic enzymes from humans and mice and is thought to provide a feedback mechanism for regulating glycolytic flux. We report the first proteomic analysis of this post-translational modification in bacteria by profiling phosphoglyceryl-lysine during the growth of *Streptococcus pyogenes* in different culture media. The identity of phosphoglyceryl-lysine was confirmed by a previously unknown diagnostic cyclic immonium ion generated during MS/MS. We identified 370 lysine phosphoglycerylation sites in 123 proteins of *S. pyogenes*. Growth in a defined medium on 1% fructose caused a significant accumulation of phosphoglycerylation compared to growth in a rich medium containing 0.2% glucose. Re-analysis of phosphoproteomes from 14 bacterial species revealed that phosphoglycerylation is generally widespread in bacteria. Many phosphoglycerylation sites were conserved in several bacteria, including *S. pyogenes*. There was considerable overlap between phosphoglycerylation, acetylation, succinylation, and other acylations on the same lysine residues. Despite some exceptions, most lysine phosphoglycerylations in *S. pyogenes* occurred with low stoichiometry. Such modifications may be meaningless, but it is also conceivable that phosphoglycerylation, acetylation, and other acylations jointly contribute to the overall regulation of metabolism.

## 1. Introduction

Post-translational modifications (PTMs) are covalent modifications that change the physicochemical properties of proteins and can thereby influence their conformation, localization, interaction with other molecules, enzymatic activity, and stability [1,2]. With the rapid development of mass spectrometry-based proteomics, the number of discrete PTMs in all domains of life has increased to more than 500 [3]. Of the bacterial PTMs, phosphorylation has been studied most intensively [1]. In recent years, N^ε^-lysine acetylation and other acyl modifications, such as succinylation, butyrylation, and malonylation, have attracted much attention [2,4,5,6]. Bacterial N^ε^-lysine acetylation can occur both enzymatically and non-enzymatically and appears to be irreversible in many cases. In bacteria, acetyl coenzyme A (AcCoA) and acetyl phosphate (AcP) can acetylate proteins non-enzymatically, with AcP appearing to be the predominant acetyl donor. Thus, there is a close association between acetylation and metabolism [2]. Recent acylome studies have typically identified several thousand modified sites in a bacterium (review in [6]). It has been suggested that these modifications fulfill different regulatory functions, but the exact regulatory mechanisms and their significance in vivo have not yet been clarified.

Another non-enzymatic protein modification at the ε-amino group of lysine is phosphoglyceryl-lysine (pgK), in which a phosphoglyceryl moiety is covalently bound to a lysine residue (referred to as phosphoglycerylation). This was first reported by Boel et al. in 2002 [7], who observed that enolases from bacteria, yeast, and rabbit muscle became covalently modified by their substrate 2-phosphoglycerate (2-PG). From experiments with labeled substrates and mass spectrometry analyses, they concluded that *Escherichia coli* enolase is probably modified via a peptide bond between the carboxyl group of 2-PG and the ε-amino group of Lys341. This residue is located in the active site of the *E. coli* enolase; accordingly, the in vitro-modified enolase lost its activity. Interestingly, the phosphoglyceryl modification of Lys341 was found to be a prerequisite for the export of enolase from cells of *E. coli* into the medium. Since in vivo only 1–2% of the intracellular enolase was phosphoglycerylated in *E. coli* and growth on different carbon sources did not influence this ratio, the authors concluded that the modification is not used to regulate the enzymatic activity. Later, Moellering and Cravatt [8] reported that the highly electrophilic acyl phosphate group of the primary glycolytic intermediate 1,3-bisphosphoglycerate (1,3-BPG) reacts with selected lysine residues in proteins to form 3-phosphoglyceryl-lysine. This reaction mechanism enabled the modification of several solvent-exposed lysine residues near the active site of glyceraldehyde-3-phosphate dehydrogenase (GAPDH). Phosphoglycerylated peptides could be identified in several protein classes in human cell lines by a standard IMAC-based phosphoproteomic workflow. In addition, a meta-analysis of published phosphoproteomic datasets confirmed that pgK-modified proteins are also present in normal mouse tissue and that several pgK sites are conserved in human and mouse protein orthologs. Enrichment of pgK sites was particularly observed in or around the active site of glycolytic enzymes. The extent of phosphoglycerylation increased in cells exposed to high glucose and proved to be reversible. Since the pgK modification inhibited the activity of glycolytic enzymes, the authors hypothesized a regulatory function of this modification [8].

Following these two experimental reports on phosphoglycerylation of lysine residues [7,8], several articles have been published dealing with the prediction of pgK sites using computational techniques, e.g., [9]. These reports are all based on 184 pgK sites identified by [8] and deposited in the Protein Lysine Modifications Database [10]. New experimental data did not appear until 2023 when the identification of 419 pgK sites in 205 proteins was reported by re-interrogating the raw MS data from a large-scale mouse liver phosphoproteomics study [11]. Most recently, an open modification re-analysis of a large-scale *Arabidopsis thaliana* mass spectrometry tissue atlas identified phosphoglycerylation on glycolytic enzymes in plants [12]. So far, there is no further knowledge about the occurrence of phosphoglycerylation in bacterial cells.

Recently, we applied a phosphoproteomic workflow to study Ser/Thr/Tyr phosphorylation in the human pathogen *Streptococcus pyogenes* [13]. In addition to phosphorylation, we found frequent pgK modifications, which prompted us to report on these separately from the phosphoproteome here. A meta-analysis of published phosphoproteomics datasets revealed the universal occurrence of phosphoglyceryl-lysine modification across bacterial groups. We also report a unique mass spectrometric signature of phosphoglyceryl-lysine.

## 2. Materials and Methods

### 2.1. Bacterial Culture Conditions

*S. pyogenes* serotype M49 strain 591 [14] was cultured in Todd Hewitt broth containing 0.2% glucose supplemented with 0.5% yeast extract (THY; Oxoid, Thermo Fisher Scientific, Darmstadt, Germany) at 37 °C under a 5% CO_2_/20% O_2_ atmosphere as previously described [13]. The bacteria were grown either THY or chemically defined medium [15] without carbon source (CDM−) or with 1% fructose (CDMF) and harvested at different time points (Figure S1).

### 2.2. Sample Preparation for Proteomics

The workflow for proteomics sample preparation and phosphopeptide enrichment was described in detail in our previous publication [13]. In brief, bacterial cells were disrupted using Precellys 24 homogenizer (peqLab Biotechnologie GmbH, Erlangen, Germany), and proteins were solubilized and denatured using a sodium deoxycholate (SDC)-containing buffer solution and a reduction/alkylation reagent containing tris(2-carboxyethyl)phosphine hydrochloride and 2-chloroacetamide. A methanol/chloroform precipitation was then carried out before the proteins were digested with trypsin in buffer solution containing SDC. After proteolytic digestion, the SDC was precipitated by acidification with trifluoroacetic acid and removed from the peptide solutions by centrifugation. Finally, the peptide solutions were desalted with OASIS HLB 1cc 30 mg Vac Cartridges (Waters, Manchester, UK). Phosphopeptide enrichment was performed using a mixture of MagReSyn TiO_2_ and MagReSyn Ti-IMAC hyperporous magnetic microparticles (ReSyn Biosciences, Edenvale, Gauteng, South Africa) as previously described [13].

### 2.3. Mass Spectrometry

LC-MS/MS analysis was performed using a nanoAcquity UPLC system (Waters, Manchester, UK) coupled to a Waters Synapt G2-S mass spectrometer equipped with a NanoLockSpray ion source as previously described [16]. For label-free quantification, the Synapt G2-S instrument was operated in data-independent mode with ion mobility separation as an additional dimension of separation (referred to as HDMS^E^). The samples from the first and third experiments (described in Section 3.2) were measured in triplicate, while the samples from the second experiment were measured in duplicate. Phosphoproteome measurements were additionally performed in data-dependent mode (DDA) as previously described [13].

### 2.4. Data Processing and Peptide and Protein Identification

The identification and quantification of the total proteome have been described previously [13]. For the simultaneous identification of phosphorylated and phosphoglycerylated peptides, raw data from data-independent (HDMS^E^) acquisitions were processed with Progenesis QI for proteomics (Nonlinear Dynamics, Newcastle upon Tyne, UK). Three different approaches for peptide and protein identification were then applied to the same data: (i) ion accounting search, (ii) Mascot search, and (iii) spectral library search (Figure S1) [13]. A database containing 1701 protein sequences from *S. pyogenes* serotype M49, strain NZ131 (UniProt release 2021_02) was searched using the ion accounting algorithm implemented in Progenesis. Trypsin was specified as the cleavage enzyme, and up to two missed cleavages were considered. Carbamidomethylation of cysteine residues was considered a fixed modification, while methionine oxidation; asparagine deamidation; phosphorylation of serine, threonine, and tyrosine residues; and phosphoglyceryl modification of lysine residues were set as variable modification. Further criteria for filtering the data were applied as described previously [13]. The fragment spectra of all remaining pgK-modified peptides were manually checked for plausibility of identification.

For identification with the Mascot search engine, HDMS^E^ data were converted to Mascot generic format (MGF) using Progenesis as previously described [13]. The database search was performed with Mascot (version 2.6.2) against the S. pyogenes database using the same enzyme specificity and fixed and variable modifications used for identification with the ion accounting algorithm. Further processing parameters of the Mascot results and the use of spectral libraries for peptide identification were also described in our previous publication [13].

Raw data from DDA acquisitions were also processed with Progenesis QI for proteomics. Export of peak lists, peptide identification using the Mascot search engine, and further processing of search results were performed as described for the HDMS^E^ data.

The changes in physicochemical and spatial properties associated with phosphoglycerylation of a lysine residue prevent cleavage by trypsin. Therefore, peptides with a single C-terminal lysine were never phosphoglycerylated unless the peptide originated from the C-terminus of a protein. However, if a peptide contained a second lysine residue immediately before or near the C-terminal lysine, the modification was sometimes incorrectly assigned to the C-terminal lysine by the search engine. In cases where the identity of the peptide was otherwise unquestionable, the position of the pgK site was corrected manually. The mass spectrometry proteomics data have been deposited to the ProteomeXchange Consortium via the PRIDE [17] partner repository with the dataset identifier PXD044423 and 10.6019/PXD044423.

### 2.5. Re-Analysis of Phosphoproteome Datasets from Other Bacteria

Mass spectrometry datasets of bacterial phosphoproteomes were retrieved from the PRIDE [17] and jPOST [18] databases of the ProteomeXchange consortium. The ProteomeXchange identifiers and other relevant details of the datasets are listed in Table S1. The raw data were processed with Progenesis to generate peak lists in Mascot generic format, as described above. Mascot searches against the UniProt protein sequence data of the respective bacterial strains (the UniProt database releases used are given in Table S1) were performed with the following parameters: Carbamidomethylation of cysteine residues was set as fixed modification and methionine oxidation, phosphorylation of serine, threonine, and tyrosine residues, as well as phosphoglyceryl modification of lysine residues as variable modifications. The peptide mass tolerance was typically set to 10 ppm and the fragment mass tolerance to 0.02 Da. Up to two missed cleaved sites of tryptic peptides were allowed. The false discovery rate was adjusted to 0.5%.

### 2.6. Additional Methods

Unsupervised hierarchical clustering of the quantified phosphoglycerylation sites was performed using the Interactive CHM Builder [19] at https://build.ngchm.net/NGCHM-web-builder/, accessed on 5 March 2024. Cluster analysis of z-transformed rows was carried out using Euclidean distance metric and average agglomeration method. Area-proportional Venn diagrams were created using BioVenn [20] at https://biovenn.nl, accessed on 2 May 2024. Multiple sequence alignments were performed using Clustal Omega 1.2.4 [21] at https://www.ebi.ac.uk/jdispatcher/msa/clustalo, accessed on 6 May 2024.

## 3. Results and Discussion

In our previous publication, we investigated growth phase- and culture medium-dependent Ser/Thr/Tyr phosphorylation in *S. pyogenes* [13]. As typical for shotgun approaches, many peptides remained unidentified in the phosphopeptide-enriched samples. Therefore, we performed an error-tolerant Mascot search [22], which indicated phosphoglycerylation of numerous lysine residues based on the mass increase of 167.98 Da. Here, we describe the analysis of this post-translational modification using the mass spectrometry data from our previous publication [13] and phosphoproteome data of other bacteria available in public data repositories.

### 3.1. A Cyclic Immonium Ion of Phosphoglyceryl-Lysine Confirms Phosphoglycerylation

**Figure 1:**
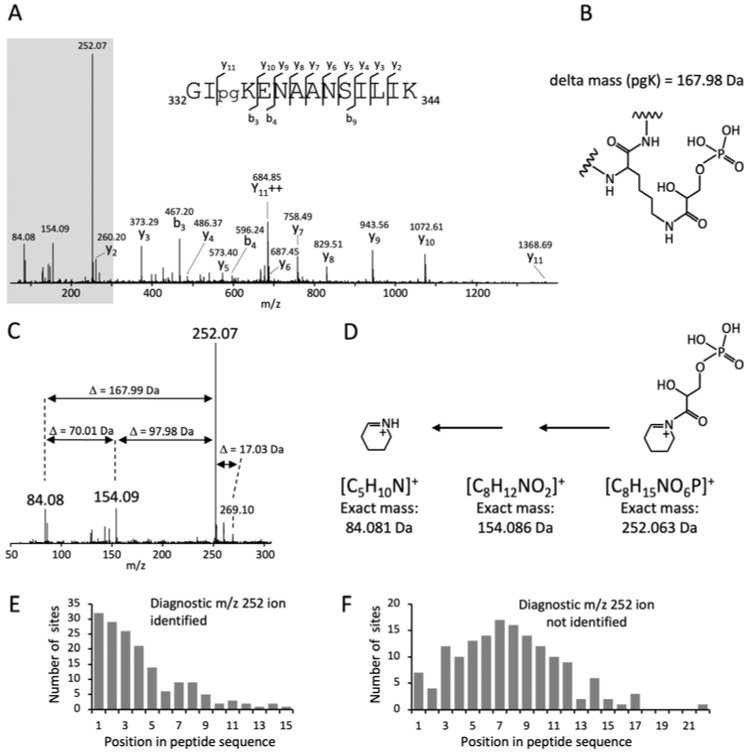
Detection of a diagnostic cyclic immonium-derived ion that confirms the pgK modification in *S. pyogenes*. (**A**) MS/MS mass spectrum of a peptide from enolase phosphoglycerylated at K334. The ions of the nearly complete y-ion series are indicated. The shaded section in shown in (**C**). (**B**) Structure of phosphoglyceryl-lysine [8]. (**C**) Section of the MS/MS spectrum shown in A. The diagnostic cyclic immonium-derived ion at *m*/*z* 252.07, the corresponding immonium ion at *m*/*z* 269.10, and the ions arising from the *m*/*z* 252.07 ion by neutral losses are labeled. (**D**) Proposed structures and formulas of the cyclic immonium-derived ions shown in C. (**E**,**F**) Dependence of the diagnostic *m*/*z* 252.07 ion on the distance of the pgK site from the N-terminus of the peptide shown for the peptides in whose MS/MS spectra the *m*/*z* 252 ion was detected (**E**) or not detected (**F**).

To rule out misidentification due to isobaric modifications, we searched the mass spectra for diagnostic ions that clearly confirm the pgK modification. Those diagnostic ions can be generated during peptide fragmentation by the neutral loss of modified side chains, such as the loss of phosphoric acid (−97.98 Da) from phosphorylated Ser and Thr residues [23], or in the form of fragments of individual amino acids, so-called immonium ions and immonium-related ions [24,25]. However, no diagnostic ions for pgK have been described to date. We found a prominent ion at *m*/*z* 252.07 in many MS/MS spectra of putative pgK-modified peptides. In some spectra it was the highest peak, accompanied by ions at *m*/*z* 154.09 and 84.08 (Figure 1A–D). The mass difference of 97.98 Da between the peaks at *m*/*z* 252.07 and *m*/*z* 154.09 corresponds to the neutral loss of phosphoric acid, and the mass difference of 167.99 Da between the peaks at *m*/*z* 252.07 and *m*/*z* 84.08 corresponds to the delta mass of the pgK modification. The difference of 70.01 Da between the ions at *m*/*z* 84.08 and 154.09 corresponds to the glycerate residue. These characteristic delta masses clearly demonstrate the phosphoglycerylation of lysine. The peak at *m*/*z* 84.08 is a common cyclic immonium-related ion in the MS/MS spectra of lysine-containing peptides, which is formed from the lysine immonium ion by loss of ammonia (–NH_3_) [26,27]. In the MS/MS spectra of pgK-modified peptides, the intensity of the *m*/*z* 84.08 ion appeared to be positively correlated with that of the *m*/*z* 252.07 ion. Although this finding is based on visual inspection of the mass spectra and was not statistically analyzed, it shows the relationship of both ions and indicates that the ion at *m*/*z* 252.07 is the cyclic immonium–NH_3_ ion of phosphoglyceryl-lysine. This was also confirmed by the occurrence of a low-intensity peak at *m*/*z* 269.10, which corresponds to the less stable immonium ion of phosphoglyceryl-lysine (Figure 1C). Thus, the ion at *m*/*z* 252.07 was identified as a new diagnostic ion for phosphoglycerylated lysine.

Cyclic immonium-related ions of acyl-lysine modifications, such as acetylation, crotonylation, succinylation, 3-hydroxypimelylation, and lactylation, to name a few, have proven to be extremely valuable for the identification and validation of acylated peptides [28,29,30,31,32]. Unfortunately, the formation of these diagnostic ions depends on the peptide characteristics, in particular, the position of the modified lysine in a peptide sequence [28,30,32]. We observed the diagnostic immonium–NH_3_ ion at *m*/*z* 252.07 in 60% of the MS/MS spectra of phosphoglycerylated peptides from *S. pyogenes*. Its intensity in relation to the other peaks within a spectrum was variable, and when it was low, the accompanying ions at *m*/*z* 154.09 and 84.08 were usually absent. The formation of the *m*/*z* 252.07 ion was significantly increased when the modified lysine residue was located at or near the N-terminus of the peptide. The average distance of the pgK site from the N-terminus of the peptide was 4.1 and 7.7 residues for the peptides with and without detected *m*/*z* 252.07 ion, respectively (Figure 1E,F). The amino acid residues surrounding the pgK site also had a certain influence on the formation of the diagnostic ion. When the pgK site was preceded by the small amino acids glycine or alanine, the formation of the diagnostic ion was supported, whereas proline prevented its formation due to increased fragmentation at the N-terminal side of proline [33].

Another feature of the phosphoglycerylated peptides was a greatly prolonged chromatographic retention time compared to their native counterparts and to those phosphorylated at Ser, Thr, or Tyr residues. From a small set of 11 groups of three peptides, we calculated mean retention time shifts of 11.9 and 8.6 min for phosphoglycerylated peptides compared to unmodified peptides and to peptides phosphorylated at Ser, Thr, or Tyr residues, respectively (Figure S2). Differentially increased peptide retention times were also caused by different types of acylation [31,32]. In summary, the immonium–NH_3_ ion at *m*/*z* 252.07 is a valuable feature for the confirmation of phosphoglyceryl-lysine, but its absence does not exclude this modification. The large shift in retention time of pgK-modified peptides can be used as a further diagnostic feature.

### 3.2. Identification of Phosphoglycerylation Depends on Protein Abundance

This study on lysine phosphoglycerylation is based on the mass spectrometric data of a previous work on Ser/Thr/Tyr phosphorylation in *S. pyogenes*, in which three growth experiments were performed [13]. In the first and second experiments, bacteria were grown until stationary phase (24 h) and late stationary phase (72 h), respectively, in three different media: (i) rich THY broth containing 0.2% glucose providing optimal growth conditions, (ii) chemically defined medium containing 1% fructose (CDMF) to induce growth on a single carbon source, and (iii) chemically defined medium without a carbon source (CDM–) to provoke starvation. In the third experiment, the bacteria were grown in THY until the late stationary phase and then transferred to fresh THY medium to determine whether the modification pattern changed within 40 min after the addition of fresh growth medium (Figure S1). Mass spectrometric analysis of enriched phosphopeptides/pgK peptides was performed using complementary methods, including both data-dependent and label-free, data-independent HDMS^E^ acquisition [13]. Four datasets were generated from each of the three experiments: (i) HDMS^E^ data searched with the ion accounting algorithm, (ii) HDMS^E^ data searched with Mascot, (iii) HDMS^E^ data searched against a spectral library, and (iv) DDA data searched with Mascot (Figure S1). For each experiment, the three identification results of the HDMS^E^ data were combined in a single Excel sheet (Excel 2016), whereas the DDA results are shown separately (Table S2).

**Figure 2:**
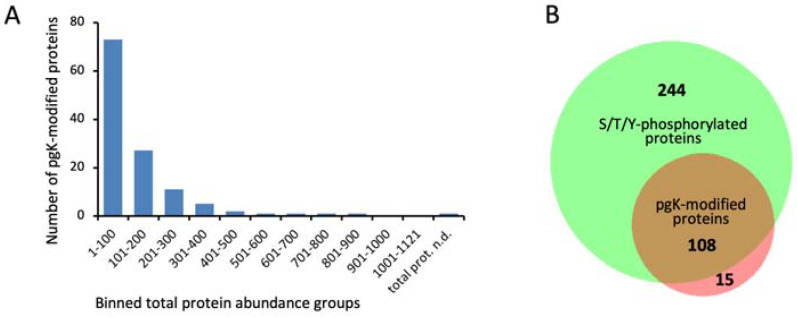
Phosphoglyceryl-lysine was mainly found on high-abundance proteins that overlap with Ser/Thr/Tyr-phosphorylated proteins. (**A**) Dependence of the identification of pgK-modified peptides on protein abundance. Each bar represents 100 proteins ordered according to decreasing abundance. (**B**) Area-proportional Venn diagram showing the overlap of Ser/Thr/Tyr-phosphorylated and pgK-modified proteins in *S. pyogenes*.

After aligning the pgK-modified peptides to 15 amino acid stretches with a central pgK site, a list of 370 lysine phosphoglycerylation sites from the three experiments was compiled (Table S3). Almost all pgK sites identified in the first and third experiments were also found in the second experiment, which included the most growth conditions, resulting in the most identifications (Figure S3). The 370 pgK sites were distributed among 123 proteins. Most pgK sites were found in the chaperone protein DnaK (22 sites), followed by enolase (Eno) (11 sites) and chaperonin GroEL (10 sites) (Table S3). Our previous analysis of the Ser/Thr/Tyr phosphoproteome of *S. pyogenes* revealed a strong correlation between protein abundance and phosphopeptide identification [13]. The same picture emerged for lysine phosphoglycerylation. Of the total 123 pgK-modified proteins, 100 (81%) were among the 200 most abundant proteins. Accordingly, 88% of the pgK-modified proteins were previously found to be Ser/Thr/Tyr-phosphorylated (Figure 2).

Obviously, the dataset of pgK-modified proteins primarily reflects protein abundance and depends on the sensitivity of the proteomics methods used. Therefore, we have refrained from a GO term analysis, which would be strongly biased by protein abundance.

### 3.3. Phosphoglycerylation Accumulates during Growth with 1% Fructose

**Figure 3:**
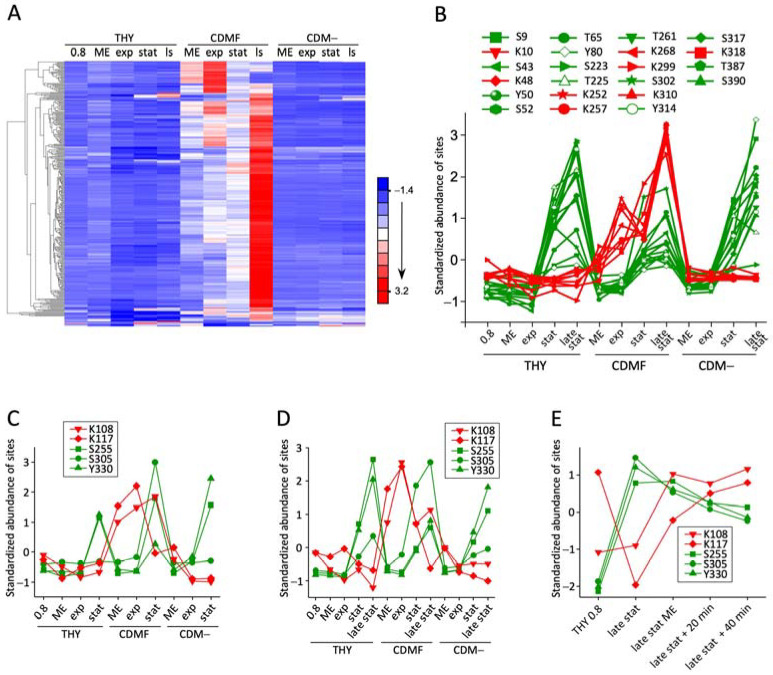
Changes in lysine phosphoglycerylation during growth of *S. pyogenes* on different culture media. (**A**) Hierarchical clustering of protein level-normalized phosphoglycerylation site abundances in *S. pyogenes* cultures at different growth phases in THY, CDMF, and CDM–. Quantitative data of 309 pgK sites from the second experiment are included. (**B**) Protein level-normalized abundance of phosphoglycerylation sites (marked in red) and Ser/Thr/Tyr phosphorylation sites (marked in green) in EF-Tu during growth of *S. pyogenes* in different culture media. (**C**–**E**) Protein level-normalized abundance of phosphoglycerylation sites (marked in red) and Ser/Thr/Tyr phosphorylation sites (marked in green) in GAPDH observed in the first (**C**), second (**D**), and third (**E**) experiments. Abbreviations: 0.8: OD_600_ = 0.8; ME: medium exchange; exp: exponential growth phase; stat: stationary phase; ls, late stat: late stationary phase.

For the quantitative analysis of dynamic lysine phosphoglycerylation, for each pgK site, the amounts of all associated ions were summarized. These included species with different charge states, missed cleavages, and additional modifications. To exclude the influence of altered protein expression, the pgK site values were normalized to the corresponding protein levels (Table S4). Hierarchical clustering was performed to visualize the dynamics of phosphoglycerylation during growth in the different media. In CDMF, in which 1% fructose is the only carbon source, phosphoglycerylation increased significantly. Of the three hundred nine pgK sites included in the quantitative analysis of the second experiment, only six sites, which clustered in the lowest part of the heat map, showed no increase in phosphoglycerylation during growth in CDMF (Figure 3A, Supplementary Data Sheet S1).

Before normalization to the protein level, however, these pgK sites were also highly elevated (Table S2). Of the six pgK sites, three belong to the archaeal S-adenosylmethionine synthetase (A0A0H3BW88) and three to the F420_ligase domain-containing protein (A0A0H3BZ61), respectively. The amount of these proteins was increased 170- and 389-fold, respectively, during growth in CDMF (Table S5). Such extreme differences pose a challenge for the label-free quantification approach used to measure protein levels. The normalization of the pgK site values to inaccurately calculated protein levels, therefore, led to an underestimation of the pgK sites in CDMF. This example shows that protein level normalization of PTM sites can lead to incorrect results in the case of a very wide range of protein abundances. The general increase of phosphoglycerylation during growth in CDMF containing 1% fructose compared to cells grown in rich THY medium containing 0.2% glucose or in CDM– was confirmed by the cluster analysis of the first experiment, which, however, included only 72 pgK sites (Supplementary Data Sheet S1).

Lysine phosphoglycerylation was highest in the stationary and late stationary phase in CDMF. In this respect, the accumulation of phosphoglycerylation is reminiscent of the global accumulation of acetylation in *E. coli*, which depends on rapid carbon flux and a carbon–nutrient imbalance that restricts growth [2]. It was found that lysine acetylation is principally correlated with the initial sugar concentration, regardless of the type of sugar [34]. The predominant acetyl donor for non-enzymatic acetylation is AcP, which is generated in the process of CoA regeneration by the phosphotransacetylase–acetate kinase pathway when the carbon flux into the AcCoA node exceeds the capacity of the TCA cycle and other central metabolic pathways. It is assumed that lysine acetylation reduces the carbon flux through the central metabolism under these conditions [2]. Similarly, the growth of *S. pyogenes* in CDMF containing 1% fructose may have resulted in an excess of the potential phosphoglyceryl donors 1,3-BPG [8] and 2-PG [7] when a carbon-free nutrient became growth-limiting, leading to an accumulation of phosphoglycerylation. In human cell cultures, 1,3-BPG concentrations increased in cells exposed to high glucose levels, and at the same time, the pgK modification state of several proteins increased [8].

In *S. pyogenes*, the dynamics of phosphoglycerylation are fundamentally different from those of Ser/Thr/Tyr phosphorylation. As previously reported [13], phosphorylation of most sites increased during the stationary phase, even in CDM– without a carbon source. This is shown in Figure 3B for 14 Ser/Thr/Tyr phosphorylation events along with eight lysine phosphoglycerylation sites in EF-Tu. In contrast to Ser/Thr/Tyr phosphorylation, lysine phosphoglycerylation only increased in CDMF, where it began to accumulate shortly after the cells were transferred to the fructose-containing medium. The same dynamics in its modifications were shown by GAPDH (Figure 3C,D), which was previously identified as a target of phosphoglycerylation in *Enterococcus faecalis*, mice, and humans [7,8]. In the third experiment, lysine phosphoglycerylation was low in the late stationary phase in THY and increased after transfer to fresh THY, in contrast to Ser/Thr/Tyr phosphorylation, which was highest in late stationary phase and slowly decreased in fresh medium (Figure 3E, Supplementary Data Sheet S1). In starving cells in CDM–, the level of phosphoglycerylation remained constant. We found no clear indication of the reversibility of the pgK modification. The slight decrease toward the stationary growth phase in THY may be related to a dilution effect with new proteins. However, it was found that the pgK modification is reversible in vitro [7,8]. Answering the question of whether pgK modification is reversible in vivo and whether there are enzymes that reverse this modification in bacteria is important for assessing the possible regulatory function of phosphoglycerylation. However, the relationship between phosphoglycerylation and the metabolic activity of the cells was clearly demonstrated. Future studies should investigate this relationship in more detail, e.g., by analyzing phosphoglycerylation in bacteria grown with gradually increasing sugar concentrations or after deprivation of a non-carbon nutrient to induce metabolic imbalances. Alternatively, strains with mutations that block certain metabolic pathways could be investigated.

### 3.4. The Phosphoglyceryl Modification Is Mostly Low-Stoichiometric

Despite a high number of 370 identified pgK sites, this modification occurs with low stoichiometry in *S. pyogenes*. In the previous publication on Ser/Thr/Tyr-phosphorylation, we calculated for one of the experiments that of a total of 3581 peptides identified after phosphopeptide enrichment, the proportion of unmodified peptides, Ser/Thr/Tyr-phosphorylated peptides, and pgK-modified peptides was 63%, 26%, and 11%, respectively. Label-free quantification revealed corresponding percentages of 73%, 24%, and 3%, respectively (see Figure S9 in reference [13]). This means that although the number of unique pgK sites is almost half the number of unique Ser/Thr/Tyr phosphorylation sites, there are quantitatively about eight times fewer pgK modification events than phosphorylations. Certainly, the occupancy of lysine phosphoglycerylation sites in most *S. pyogenes* proteins is low and may not be of appreciable physiological relevance. We, therefore, looked at the quantitatively dominant pgK sites that are most likely to be associated with biological consequences for the bacteria.

If the data were not normalized to the protein level, the quantitatively predominant pgK sites originated from the glycolytic enzymes fructose-bisphosphate aldolase class II (Fba), triosephosphate isomerase (Tpi), GAPDH, phosphoglycerate kinase (Pgk), 2_3-bisphosphoglycerate-dependent phosphoglycerate mutase (GpmA), Eno, and pyruvate kinase (Pyk), as well as from DNA-binding protein HU (Hup) and elongation factor Tu (EF-Tu) (Figure S4A,C,E). All of these proteins are among the 25 most abundant proteins in *S. pyogenes* (Table S5). The dominance of their pgK sites is to be expected if the data are not normalized to the protein level. After normalization to the protein level, the pgK sites of the glycolytic enzymes Tpi (K216), Pgk (K126), GpmA (K98), and Pyk (K383), as well as of GTP cyclohydrolase 1 (Gch1, K110), glucose-1-phosphate thymidydyltransferase (K275), and CsbD domain-containing protein (CsbD), were most abundant, indicating a relatively high occupancy of lysine phosphoglycerylation sites (Figure S4B,D,F). It should be noted that the measured peptide abundance also depends on the specific ionization efficiency of each peptide, which makes label-free quantification of individual peptides imprecise.

Interestingly, CsbD was represented by six pgK sites among the quantitatively dominant phosphoglycerylation events. CsbD is classified as a general bacterial stress response protein of the YjbJ superfamily (IPR036629). Thus, strong phosphoglycerylation of CsbD could indicate a stress response in *S. pyogenes*. However, the lysine richness of the small 66 amino acids encompassing CsbD protein leads to several tryptic peptides consisting of only five amino acids that are excluded during proteome identification (Figure S4G). As a result, the amount of unmodified protein may have been underestimated in label-free quantification, resulting in values that are too high for the pgK sides when normalized to the amount of protein. In this context, the importance of CsbD as a target of phosphoglycerylation needs to be interpreted with caution.

In summary, it was found that proteins of *S. pyogenes* undergo phosphoglycerylation at many lysine residues. The modification accumulates during growth with 1% fructose as the sole carbon source but remains low in stoichiometry. A few pgK sites, such as K98 of GpmA, appear to have a higher occupancy and may be of particular physiological importance.

### 3.5. Phosphoglycerylation Is Conserved in Bacteria and Overlaps with Acylation

The unexpectedly frequent occurrence of phosphoglycerylation in *S. pyogenes* raises the question of phosphoglycerylation in other bacteria. Therefore, we analyzed raw data from 15 phosphoproteome projects [35,36,37,38,39,40,41,42,43,44,45,46,47,48,49] comprising 14 bacterial species for the occurrence of the pgK modification. The raw data were downloaded from public data repositories, using only a selection of files from comprehensive datasets (Table S1). Surprisingly, we identified peptides with pgK modifications in all datasets (Table S6). The diagnostic ion at *m*/*z* 252.07 was widespread in the MS/MS spectra of these peptides.

**Figure 4:**
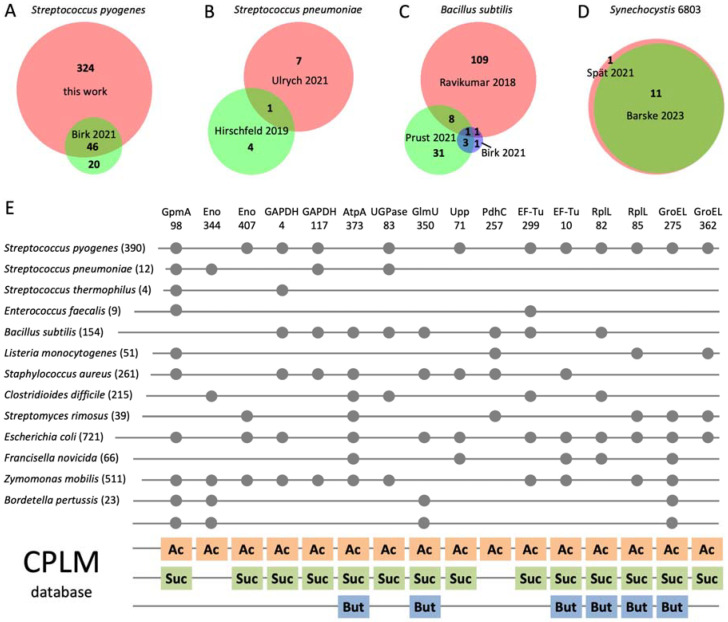
Widespread phosphoglycerylation in bacteria. (**A**–**D**) Area-proportional Venn diagrams showing the overlap of pgK sites identified by re-analysis of different phosphoproteome datasets from *S. pyogenes* [35] (**A**), *S. pneumoniae* [36,37] (**B**), *B. subtilis* [35,38,39] (**C**), and *Synechocystis* 6803 [40,41] (**D**). The number of identified pgK sites is indicated, and references for the datasets are given in square brackets. (**E**) Conserved pgK sites in bacteria. The pgK sites identified from different datasets of the same species (see **A**–**D**) were merged. The number of unique pgK sites is given in brackets after the name of the respective species. The amino acid position of the pgK site indicated under the short name of the protein corresponds to the proteins of *S. pyogenes*, with the exception of PdhC, which corresponds to *B. subtilis*. Phosphoglycerylation is indicated by a grey circle. Modification sites that overlap with acetylation (Ac), succinylation (Suc), and butyrylation (But) according to the Compendium of Protein Lysine Modifications 4.0 (CPLM 4.0) [10] are indicated in the lower part of the figure. Abbreviations: GAPDH, glyceraldehyde-3-phosphate dehydrogenase; GpmA, 2_3-bisphosphoglycerate-dependent phosphoglycerate mutase; Eno, enolase; AtpA, alpha chain of the ATP synthase; UGPase, UTP–glucose-1-phosphate uridylyltransferase; GlmU, bifunctional protein GlmU; Upp, uracil phosphoribosyltransferase; PdhC, dihydrolipoamide acetyltransferase component of the pyruvate dehydrogenase complex; EF-Tu, elongation factor Tu; RplL, large ribosomal subunit bL12; GroEL, 60 kDa chaperonin.

First, we investigated the concordance of pgK sites identified in different datasets of the same bacterial species. Of the 60 pgK sites identified in *S. pyogenes* M1 [35], 42 matched the 370 sites we identified in *S. pyogenes* M49, indicating a high conservation of pgK sites in *S. pyogenes* (Figure 4A). In contrast, there was only one match (K29 of acetate kinase) between the few pgK sites identified in two datasets of *Streptococcus pneumoniae* [36,37] (Figure 4B). Also, the concordance of pgK sites between three datasets of *Bacillus subtilis* [35,38,39] was relatively low; only K224 of GAPDH was consistently present (Figure 4C). However, almost completely matching pgK sites were identified in two datasets of the cyanobacterium *Synechocystis* 6803 (Figure 4D). This high level of consistency could be due to the fact that both proteome projects were carried out by the same scientific group [40,41].

We identified between four and seven hundred twenty-one pgK sites in the different bacteria (Figure 4E). These numbers should not be taken as an indicator of the frequency of phosphoglycerylation in a particular bacterial species, as it may depend on several factors, such as the growth conditions of the bacteria, the method of phosphopeptide enrichment, the number of raw data files used for the database search, the suitability of our database search approach for the specific data, and, last but not least, the depth of the respective phosphoproteome analysis. The influence of the latter factor is obvious, as we identified a particularly large number of pgK sites from the raw data of *B. subtilis* [38], *Staphylococcus aureus* [39], *Clostridioides difficile* [44], and *E. coli* [46], in each of whose phosphoproteomes more than 1500 phosphorylation sites were reported. The only exception with significantly more pgK than Ser/Thr/Tyr phosphorylation sites was found in *Zymomonas mobilis*, where we identified 511 pgK sites, but only 363 phosphorylation sites were reported [48]. This could be related to the particular metabolism of this diazotrophic bacterium and its cultivation with a high glucose concentration of 2%, just as phosphoglycerylation was increased in *S. pyogenes* when grown on 1% fructose.

Similar to *S. pyogenes*, the pgK sites in the different bacteria were mainly found in glycolytic and other enzymes of the central metabolism, ribosomal proteins, elongation factors, and chaperones. The specific metabolic pathways of *C. difficile* and *Z. mobilis* provided additional targets for phosphoglycerylation. In the anaerobe *C. difficile*, enzymes of the acid biosynthesis pathway, such as pyruvate:ferredoxin oxidoreductase, formate acetyltransferase, and 3-hydroxybutyryl-CoA dehydrogenase, as well as the putative oxidative stress protein rubrerythrin, contained several pgK sites. In the diazotrophic bacterium *Z. mobilis*, most pgK modifications (17 sites) were found in the alpha chain of the nitrogenase complex, while other proteins involved in nitrogen fixation also had many pgK sites. Accordingly, in *Synechocystis* 6803, which belongs to the cyanobacteria that are the only oxygenic photosynthetic bacteria, proteins involved in photosynthesis were found to be phosphoglycerylated (Table S6).

Despite the diversity of metabolic pathways, many pgK sites were conserved in different bacterial species (Figures S5–S18). PgK sites found in at least four of the bacterial species tested are shown in Figure 4E. The sites are distributed across 11 proteins with a broad spectrum of biological functions. The most common pgK site was K98 from GpmA, which was found in nine of the fourteen bacterial species. GpmA is not produced in *B. subtilis* [50] and is also not known for *C. difficile*, *Francisella novicida*, and *Synechocystis* 6803. Of the GpmA-producing bacteria, only *Streptomyces rimosus* was not found to exhibit phosphoglycerylation at K98. GpmA catalyzes the interconversion of 2-PG and 3-PG and requires 1,3-BPG as a cofactor. Since K98 is involved in substrate binding [8,11], the phosphoglycerylation of this site is well explainable. Other glycolytic enzymes with frequent pgK modifications were Eno and GAPDH. K344 of Eno, situated in the active site of the enzyme, corresponds to the site of modification in *E. coli* described in the first publication on phosphoglycerylation [7] and was also phosphoglycerylated in Eno from humans and mice [8,11]. A further very frequent pgK site was K373 of the regulatory alpha chain of the ATP synthase (AtpA), which was found in seven bacteria. UTP–glucose-1-phosphate uridylyltransferase (UGPase), bifunctional protein GlmU (GlmU), uracil phosphoribosyltransferase (Upp), and the dihydrolipoamide acetyltransferase component of the pyruvate dehydrogenase complex (PdhC) also possessed pgK sites that were conserved in various bacteria. What these proteins have in common is the conversion of energy-rich phosphate compounds such as AcCoA and di- and triphosphates. Furthermore, EF-Tu, the protein of the large ribosomal subunit bL12 (RplL), and the 60 kDa chaperonin (GroEL) contained several pgK sites that are common to different bacteria (Figure 4E).

Of the 12 phosphoglycerylated proteins found in *Synechocystis* 6803, only one pgK site of Gch1 was also present in *S. pyogenes*, *E. coli*, and *Z. mobilis* (Figure S13). Gch1 hydrolyzes guanosine triphosphate (GTP) in the biosynthetic pathway of biopterin. In summary, it can be seen that proteins involved in the conversion of energy-rich compounds are phosphoglycerylated particularly frequently.

Next, we compared the pgK sites with the bacterial lysine modification sites included in the Compendium of Protein Lysine Modifications 4.0 (CPLM 4.0) [10]. Surprisingly, all pgK sites listed in Figure 4E are also targets of acetylation, often in several different bacteria, and, with the exception of two sites, also of succinylation. A striking overlap between acetylated and succinylated lysines has been described previously [6,51]. Additionally, several of the sites were found butyrylated in *Clostridium acetobutylicum* (Figure 4E). The overlap between phosphoglycerylated lysines and different types of acylated lysines will be even greater if the recently published acylome analyses not yet included in CPLM 4.0 are taken into account, e.g., [6,52,53,54]. The coincidence of phosphoglycerylation and acetylation sites also applied to *Synechocystis* 6803, as six of the twelve pgK sites (Table S6) were previously found in the acetylome [55].

K98 of GpmA and other lysine residues of glycolytic enzymes were identified as conserved acetylation sites in a proteomic analysis of 48 phylogenetically distinct bacteria without enrichment of acetylated peptides prior to mass spectrometric analysis [5]. This shows that acetylation can be analyzed without the usual immunoaffinity enrichment. Therefore, we re-searched our mass spectrometry data of the *S. pyogenes* proteome for lysine acetylation and identified 14 acetylation sites. Of these, 12 acetylation sites, including K98 of GpmA and K85 of RplL, listed in Figure 4E, matched the pgK sites in *S. pyogenes* (Table S7). Thus, phosphoglycerylated and acetylated lysines overlap in *S. pyogenes* as well as in other bacteria.

### 3.6. Conclusions

Our analysis revealed several similarities between lysine phosphoglycerylation and ac(et)ylation in bacteria. Both modifications accumulate in cells exposed to high concentrations of a carbon source, leading to a rapid carbon flux and possibly to a carbon–nutrient imbalance [2]. Phosphoglycerylation and non-enzymatic acetylation are closely related to the concentration of the phosphoglyceryl and acetyl donors 1,3-BPG and AcP, respectively. Both modifications were preferentially found in proteins of the central metabolism and protein biosynthesis and additionally in proteins of specific biosynthetic pathways such as nitrogen fixation [56], photosynthesis [55], and anaerobic fermentation [57]. Both modifications often affect the same lysine residues of bacterial proteins. Matching phosphoglycerylation, acetylation, and succinylation sites were also found in the murine liver proteome [11].

Acetylation neutralizes the positive charge of the lysine residue, while phosphoglycerylation reverses the charge by adding a negatively charged moiety, both of which can severely impair structure and function. Several in vitro or in silico studies have shown that acetylation [5,58,59,60,61] and phosphoglycerylation [7,8] alter enzyme activity, especially when the modification affects the active site of a protein. Consequently, regulatory mechanisms of carbon flux and glycolytic activity through phosphoglycerylation [8] and acetylation [4] have been postulated. We have identified several pgK sites that could potentially fulfill a regulatory function due to their relatively high abundance and site occupancy, as well as their conservation among bacteria. Most noteworthy is the phosphoglycerylation of K98 of GpmA, which is also a conserved acylation site in bacteria [5]. Interestingly, the corresponding site in GpmA of *E. coli* was acetylated with high stoichiometry in vitro, in contrast to several other enzymes tested [60]. On the other hand, phosphoglycerylation occurred in proteins with a wide range of biological functions, and most pgK sites were modified with a low stoichiometry, making a direct regulatory effect unlikely. And in general, phosphoglycerylation can be detrimental to protein function. Therefore, phosphoglycerylation should be considered beyond its possible role in the regulation of glycolysis by specific pgK sites. An interesting consideration arises from the close coupling between metabolic activity and acetylation, which manifests itself in the fact that proteins are acetylated at accessible lysines as soon as an excess of acetyl donors is present. Schilling [34] therefore formulated the question, “how does the cell cope with, or perhaps utilize for its advantage, acetylation that it cannot avoid”? One possibility is that acetylation disrupts metabolic, transcriptional, and translational protein complexes and thus serves as a rheostat to tune down the flux of carbon and optimize growth [2]. These considerations can be transferred to phosphoglycerylation. It is conceivable that phosphoglycerylation, acetylation, and other acylations, which often overlap at the same lysine residues, jointly contribute to the general regulation of metabolism. Alternatively, phosphoglycerylated lysines could serve to store energy, as has been discussed for acetylation, or they could be benign and have no function [2]. Further work is needed to explore lysine phosphoglycerylation in bacteria.

## Data Availability

The mass spectrometry proteomics data have been deposited to the ProteomeXchange Consortium via the PRIDE [17] partner repository with the dataset identifier PXD044423 and 10.6019/PXD044423.

## Supplementary Materials

The following supporting information can be downloaded at https://www.mdpi.com/article/10.3390/microorganisms12081556/s1. Figure S1: Bacterial growth and proteomics workflow; Figure S2: Shift in retention time in reversed-phase chromatography due to phosphoglycerylation; Figure S3: Venn diagram comparing pgK sites identified in three experiments; Figure S4: Quantitatively predominant phosphoglycerylation events in *S. pyogenes;* Figures S5–S18: Protein sequence alignments indicating conserved pgK sites in bacteria; Table S1: List of mass spectrometry data retrieved from ProteomeXchange; Table S2: Detailed data on pgK site identification in *S. pyogenes*; Table S3: List of 370 pgK sites and 123 phosphoglycerylated proteins identified in *S. pyogenes*; Table S4: Quantification of pgK sites; Table S5: Label-free quantification of the *S. pyogenes* proteome; Table S6: pgK sites identified in 18 mass spectrometry datasets from 14 bacterial species; Table S7: Acetylated peptides of *S. pyogenes* identified without prior enrichment and their overlap with phosphoglycerylation; Data Sheet S1: Cluster analyses of pgK site abundances.

## Abbreviations

THY: Todd Hewitt broth supplemented with yeast extract; CDM–: chemically defined medium without carbon source; CDMF: chemically defined medium with fructose; HDMS^E^: data-independent acquisition mode with ion mobility separation as an additional dimension of separation; DDA: data-dependent acquisition.
