# Supplementary material for: Lysine Phoshoglycerylation Is Widespread in Bacteria and Overlaps with Acylation
Source: Microorganisms. 2024 Jul 30;12(8):1556. doi: 10.3390/microorganisms12081556 (PMC11356508; doi:10.3390/microorganisms12081556)
Supplement: Supplementary file 1 [file microorganisms-12-01556-s001.zip › Suppl Figures S1-S18.pdf]

Supplementary figures S1-S18

Lysine phoshoglycerylation is widespread in bacteria and overlaps with acylation

Stefan Mikkat[1]*, Michael Kreutzer[2], Nadja Patenge[3]

[1] Core Facility Proteome Analysis, Rostock University Medical Center, Rostock, Germany
[2] Medical Research Center, Rostock University Medical Center, Rostock, Germany
[3] Institute of Medical Microbiology, Virology and Hygiene, Rostock University Medical Center, Rostock, Germany

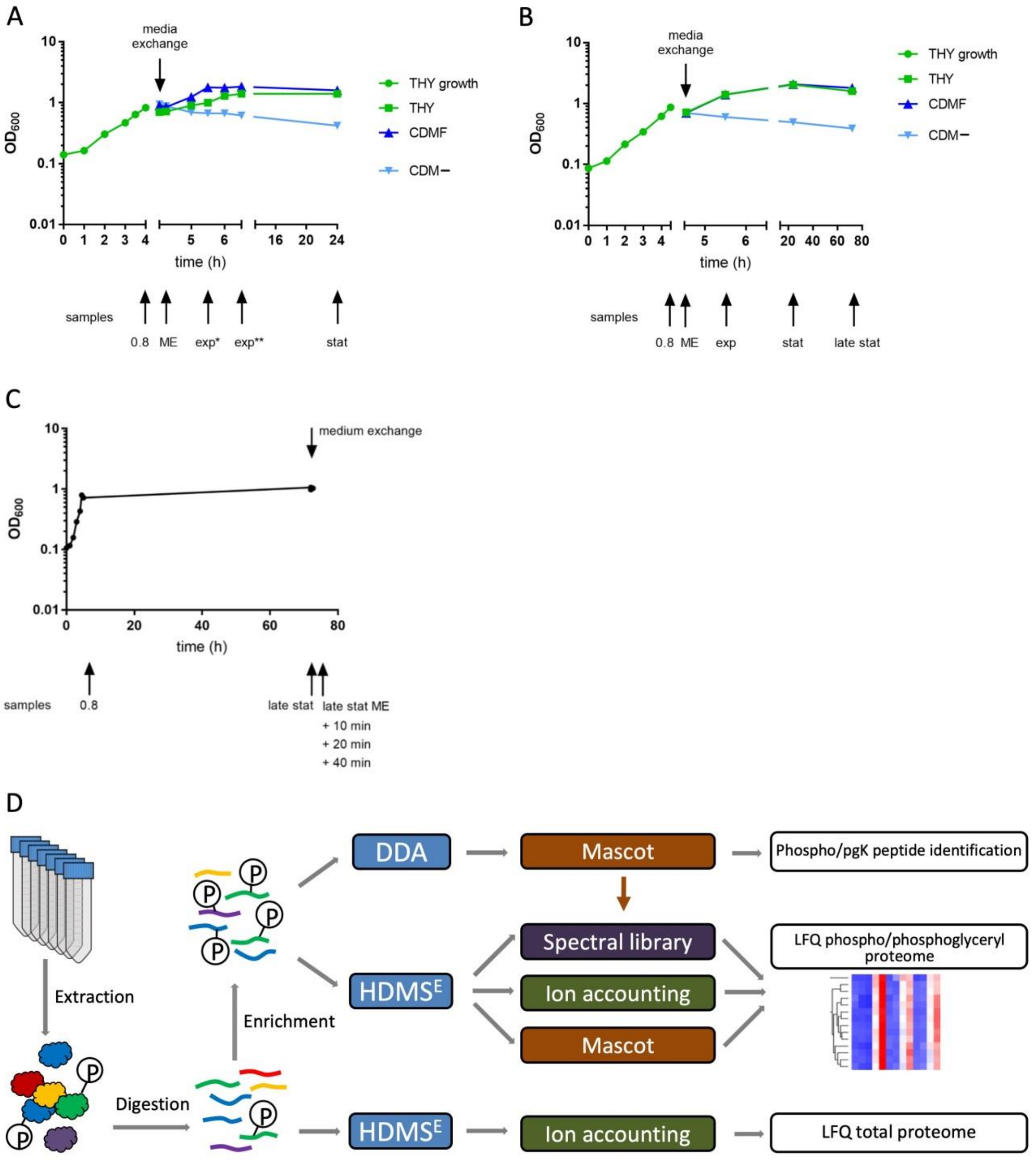

Figure S1. Bacterial growth and proteomics workflow. **(A,B)** *S. pyogenes* was grown in THY to an OD_{600} = 0.8 (green circles). For media exchange (ME), bacterial cultures were centrifuged and pellets were suspended in either THY (THY, green squares), CDM without carbon source (CDM–, light blue triangles) or CDM with 1% fructose (CDMF, dark blue triangles). Sample collection is indicated by arrows. exp: exponential growth phase (one doubling), stat: stationary phase, late stat: late stationary phase. **(C)** *S. pyogenes* was grown in THY for 72 h until the late stationary phase. For medium exchange (ME), bacterial cultures were centrifuged, pellets were suspended in fresh THY and incubated for 10, 20, and 40 min. Sample collection is indicated by arrows. 0.8: OD_{600} = 0.8; late stat: late stationary phase. **(D)** Experimental workflow for proteomic and phosphoproteomic nano LC-MS/MS analysis using a Synapt G2-S mass spectrometer and the analysis software Progenesis QI for proteomics. For label-free quantification (LFQ) of the total proteome, tryptic digests of the extracted proteins were subjected to data-independent $HDMS^E$ acquisition. The ion accounting algorithm implemented in Progenesis was used for peptide and protein identification. Enriched phosphopeptides were subjected to both data-dependent (DDA) and $HDMS^E$ acquisition. The peak lists from the DDA measurements were exported for identification by the Mascot search engine. The $HDMS^E$ data were subjected to peptide identification by Mascot, ion accounting and comparison with a spectral library assembled from phosphopeptides identified in the DDA/Mascot approach (indicated by the brown arrow). The figures were previously published in [13].

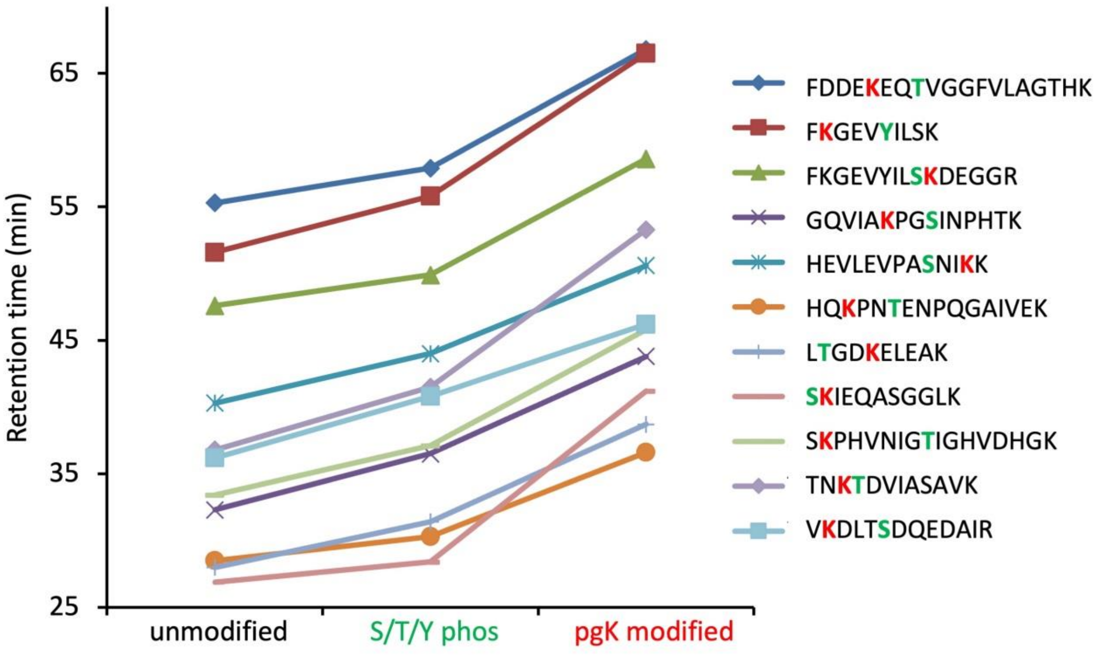

Figure S2. Shift in retention time in reversed-phase chromatography due to phosphoglycerylation compared to unmodified and phosphorylated peptides.

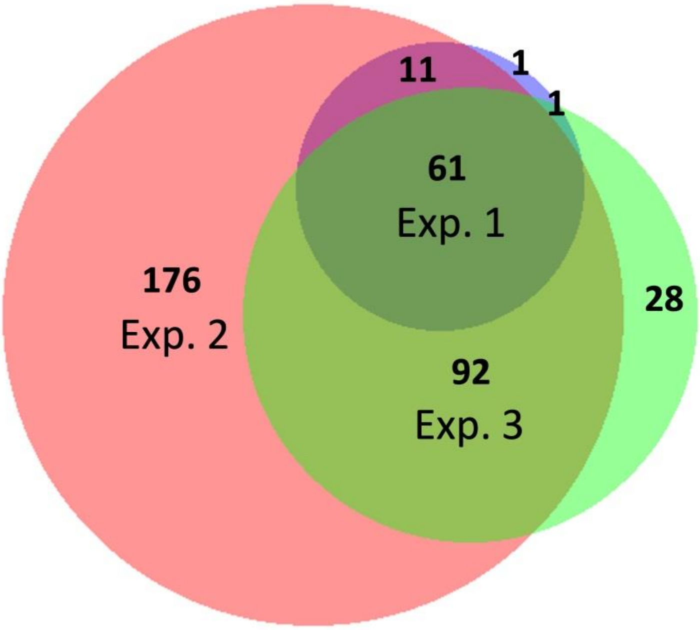

Figure S3. Area-proportional Venn diagram showing the number and overlap of pgK sites identified in each of the three experiments.

A

Exponential growth in THY; not protein level-normalized

Acc. No	Protein name	pgK site
A0A0H3C1H3	Fructose-bisphosphate aldolase class II	K124
A0A0H3C1H3	Fructose-bisphosphate aldolase class II	K274
B5XKI2	Triosephosphate isomerase	K216
A0A0H3BYM9	Glyceraldehyde-3-phosphate dehydrogenase	K108
A0A0H3BYM9	Glyceraldehyde-3-phosphate dehydrogenase	K117
B5XIF1	Phosphoglycerate kinase	K126
B5XIF1	Phosphoglycerate kinase	K130
B5XM69	2_3-bisphosphoglycerate-dependent phosphoglycerate mutase	K98
B5XKM7	Enolase	K428
A0A0H3BYL1	Pyruvate kinase	K383
B5XLE8	GTP cyclohydrolase 1	K110
A0A0H3C0P8	DNA-binding protein HU	K19
A0A0H3C0P8	DNA-binding protein HU	K87
A0A0H3C010	LSU ribosomal protein L7/L12 (L23e)	K27
B5XJ48	50S ribosomal protein L5	K121
B5XKI1	Elongation factor Tu	K299

B

Exponential growth in THY; protein level-normalized

Acc. No	Protein name	pgK site
B5XKI2	Triosephosphate isomerase	K216
B5XIF1	Phosphoglycerate kinase	K126
B5XIF1	Phosphoglycerate kinase	K130
B5XM69	2_3-bisphosphoglycerate-dependent phosphoglycerate mutase	K98
A0A0H3BYL1	Pyruvate kinase	K383
B5XLE8	GTP cyclohydrolase 1	K110
B5XJD9	Acetate kinase	K45
A0A0H3BYI5	Glucose-1-phosphate thymidylyltransferase	K275
A0A0H3C1S0	CsbD domain-containing protein	K9
A0A0H3C1S0	CsbD domain-containing protein	K18
A0A0H3C1S0	CsbD domain-containing protein	K23
A0A0H3C1S0	CsbD domain-containing protein	K28
A0A0H3C1S0	CsbD domain-containing protein	K44
A0A0H3C1S0	CsbD domain-containing protein	K62

C

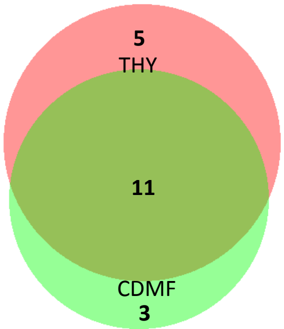

D

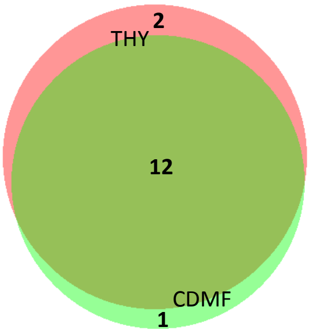

E

Stationary phase in CDMF; not protein level-normalized

Acc. No	Protein name	pgK site
A0A0H3C1H3	Fructose-bisphosphate aldolase class II	K274
B5XKI2	Triosephosphate isomerase	K12
B5XKI2	Triosephosphate isomerase	K216
A0A0H3BYM9	Glyceraldehyde-3-phosphate dehydrogenase	K108
B5XIF1	Phosphoglycerate kinase	K126
B5XIF1	Phosphoglycerate kinase	K130
B5XM69	2_3-bisphosphoglycerate-dependent phosphoglycerate mutase	K98
B5XKM7	Enolase	K190
B5XKM7	Enolase	K428
A0A0H3BYL1	Pyruvate kinase	K383
A0A0H3C1S0	CsbD domain-containing protein	K18
A0A0H3C0P8	DNA-binding protein HU	K19
A0A0H3C0P8	DNA-binding protein HU	K87
B5XKI1	Elongation factor Tu	K299

F

Stationary phase in CDMF; protein level-normalized

Acc. No	Protein name	pgK site
B5XKI2	Triosephosphate isomerase	K216
B5XIF1	Phosphoglycerate kinase	K126
B5XM69	2_3-bisphosphoglycerate-dependent phosphoglycerate mutase	K98
A0A0H3BYL1	Pyruvate kinase	K383
B5XLE8	GTP cyclohydrolase 1	K110
A0A0H3BYI5	Glucose-1-phosphate thymidylyltransferase	K275
A0A0H3C2K4	Alkyl hydroperoxide reductase C	K159
A0A0H3C1S0	CsbD domain-containing protein	K9
A0A0H3C1S0	CsbD domain-containing protein	K18
A0A0H3C1S0	CsbD domain-containing protein	K23
A0A0H3C1S0	CsbD domain-containing protein	K28
A0A0H3C1S0	CsbD domain-containing protein	K44
A0A0H3C1S0	CsbD domain-containing protein	K62

G MSEEKLKSKIEQASGGLKEGAGKLTGDKELEAKGFVEKTIAKGKELADDAKEAVEGAVDAVKEKLK

Figure S4. Quantitatively predominant phosphoglycerylation events in *S. pyogenes*. The 25 most abundant pgK sites were selected from both exponentially growing cultures in THY and stationary phase cultures in CDMF. This was done with experiments 1 and 2, and only the matched pgK sites between both experiments are shown. **(A)** Predominant pgK sites during the exponential growth of THY. No normalization to the corresponding protein levels was performed. (**B**) Predominant pgK sites during the exponential growth of THY. PgK site values were normalized to the corresponding protein levels. **(E)** Predominant pgK sites in stationary phase cultures in CDMF. No normalization to the corresponding protein levels was performed. **(F)** Predominant pgK sites in stationary phase cultures in CDMF. PgK site values were normalized to the corresponding protein levels. **(C)** Venn diagrams showing the correspondence of pgK sites in THY and CDMF for the data not normalized to protein level and **(D)** for the data normalized to protein level. **(G)** Amino acid sequence of the CsbD domain-containing protein (A0A0H3C1S0). Lysine residues are highlighted with red letters, pgK sites belonging to the quantitatively predominant sites after normalization to protein level are highlighted in green, and other identified pgK sites are highlighted in blue.

```
tr|L8EU38|L8EU38_STRR1                MADAPYKLILLRHGESEWNAKNLFTGWVDVNLNEKGEKEAVRGGELLKDAGLLPDVVHTS      60
tr|A0A0E7UZS0|A0A0E7UZS0_BORPT        ----MYKLVLMRHGESQWNLENRFTGWTDVDLTETGREQARKAGELLKREGYAFDLAYTS      56
sp|P62707|GPMA_ECOLI                  --MAVTKLVLVRHGESQWNKENRFTGWYDVDLSEKGVSEAKAAGKLLKEEGYSFDFAYTS      58
sp|Q2FE81|GPMA_STAA3                  ----MPKLILCRHGQSEWNAKNLFTGWEDVNLSEQGINEATRAGEKVRENNIAIDVAFTS      56
sp|P30798|GPMA_ZYMMO                  ----MPTLVLSRHGQSEWNLENRFTGWWDVNLTEQGVQEATAGGKALAEKGFEFDIAFTS      56
tr|J7M1Q4|J7M1Q4_STRP1                ----MVKLVFARHGESEWNKANLFTGWADVDLSEKGTQQAIDAGKLIKEAGIEFDLAFTS      56
sp|B5XM69|GPMA_STRPZ                  ----MVKLVFARHGESEWNKANLFTGWADVDLSEKGTQQAIDAGKLIKEAGIEFDLAFTS      56
sp|Q04JB4|GPMA_STRP2                  ----MVKLVFARHGESEWNKANLFTGWADVDLSEKGTQQAIDAGKLIKEAGIEFDQAYTS      56
tr|A0A0E2QGW3|A0A0E2QGW3_STRTR        ----MVKLVFARHGESEWNKANLFTGWADVDLSEKGTQQAIDAGKLIKEAGIEFDKAYTS      56
sp|Q839H4|GPMA_ENTFA                  ----MPKLVFSRHGLSEWNALNQFTGWADVDLAPEGVEEAKEGGRKIKEAGIEFDVAYTS      56
sp|Q8Y571|GPMA_LISMO                  -----MKLVLIRHGQSEWNKLNLFTGWHDVDLSQEGVVEAMTAGKRIKEAGLEFDVAFTS      55
                                            .*:: *** *:**  * **** **:*   *  :*  .*. :   .   * ..**

tr|L8EU38|L8EU38_STRR1                VQKRAIRTAQLALEAADRHWIPVHRSWRLNERHYGALQGKDKAQTLAEFGEEQFMLWRRS      120
tr|A0A0E7UZS0|A0A0E7UZS0_BORPT        VLKRAIRTLWIALDAMDAMYTPVGINWRLNERHYGQLQGLNKAETAAKYGDEQVLIWRRA      116
sp|P62707|GPMA_ECOLI                  VLKRAIHTLWNVLDELDQAWLPVEKSWKLNERHYGALQGLNKAETAEKYGDEQVKQWRRG      118
sp|Q2FE81|GPMA_STAA3                  LLTRALDTTHYILTESKQQWIPVYKSWRLNERHYGGLQGLNKDDARKEFGEEQVHIWRRS      116
sp|P30798|GPMA_ZYMMO                  VLTRAIKTTNLILEAGKTLWVPTEKDWRLNERHYGGLTGLNKAETAAKHGEEQVHIWRRS      116
tr|J7M1Q4|J7M1Q4_STRP1                VLTRAIKTTNLALENAGQLWVPTEKSWRLNERHYGALTGKNKAEAAEQFGDEQVHIWRRS      116
sp|B5XM69|GPMA_STRPZ                  VLTRAIKTTNLALENAGQLWVPTEKSWRLNERHYGALTGKNKAEAAEQFGDEQVHIWRRS      116
sp|Q04JB4|GPMA_STRP2                  VLKRAIKTTNLALEASDQLWVPVEKSWRLNERHYGGLTGKNKAEAAEQFGDEQVHIWRRS      116
tr|A0A0E2QGW3|A0A0E2QGW3_STRTR        VLKRAIKTTNLALEASDQLWVPVEKSWRLNERHYGGLTGKNKAEAAEQFGDEQVHIWRRS      116
sp|Q839H4|GPMA_ENTFA                  VLTRAIKTCNYLLEYSDQLWVPQIKSWRLNERHYGKLQGLNKKETAEKYGDEQVHIWRRS      116
sp|Q8Y571|GPMA_LISMO                  VLTRAIKTLNYVLEESDQMWVPVHKSWRLNERHYGALQGLNKQETAEKYGADQVQKWRRS      115
                                      : .**: *    *      : *   .*:******* * * :* ::  :.* :*.  ***.

tr|L8EU38|L8EU38_STRR1                YDTPPPPLADDAEFSQIDDPRYAIIPSELRPRTECLKDVVGRMLPYWYDSIVPDLSAGRT      180
tr|A0A0E7UZS0|A0A0E7UZS0_BORPT        YAIAPEPLDLEDPRHPRFDGRYAKIPADQLPATECLKDTVARVLPFWNESIAPAIRAGRR      176
sp|P62707|GPMA_ECOLI                  FAVTPPELTKDDERYPGHDPRYAKLSEKELPLTESLALTIDRVIPYWNETILPRMKSGER      178
sp|Q2FE81|GPMA_STAA3                  YDVKPPAETEEQREAYLADRRYNHLDKRMMPYSESLKDTLVRVIPFWTDHISQYLLDGQT      176
sp|P30798|GPMA_ZYMMO                  YDVPPPPMEKGSKFDLSGDRRYDG---VKIPETESLKDTVARVLPYWEERIAPELKAGKR      173
tr|J7M1Q4|J7M1Q4_STRP1                YDVLPPAMAKDDEYSAHKDRRYADLDPALIPDAENLKVTLERAMPYWEEKIAPALLDGKN      176
sp|B5XM69|GPMA_STRPZ                  YDVLPPAMAKDDEYSAHKDRRYADLDPALIPDAENLKVTLERAMPYWEEKIAPALLDGKN      176
sp|Q04JB4|GPMA_STRP2                  YDVLPPNMDRDDEHSAHTDRRYASLDDSVIPDAENLKVTLERALPFWEDKIAPALKDGKN      176
tr|A0A0E2QGW3|A0A0E2QGW3_STRTR        YDVLPPKMDRDDEYSAHKDRRYASLDDSVIPDAENLKVTLERALPFWEDKIAPALKDGKN      176
sp|Q839H4|GPMA_ENTFA                  YDTLPPLMEATDEGSAANDRRYAMLDQRDIPGGENLKVTLERALPFWQDEIAPALKDNKT      176
sp|Q8Y571|GPMA_LISMO                  YDTLPPLLEENDERQAKNDRRYQLLDTHAIPSGENLKVTLERVIPYWMDTIAPEIKEGRR      175
                                      :   *             * **        *  * *  .: * :*:* : *   :  ..

tr|L8EU38|L8EU38_STRR1                VLVAAHGNSLRALVKHLDGISDADIAALNIPTGIPLSYELDADFHPVTPGGTYLDPEA-A      239
tr|A0A0E7UZS0|A0A0E7UZS0_BORPT        VLVAAHGNSLRALIKHLDNVSDDDIVGVNIPTGQPLVYELDEDLKPIR--HYYLGDAAEI      234
sp|P62707|GPMA_ECOLI                  VIIAAHGNSLRALVKYLDNMSEEEILELNIPTGVPLVYEFDENFKPLK--RYYLGNADEI      236
sp|Q2FE81|GPMA_STAA3                  VLVSAHGNSIRALIKYLEDVSDEDIINYEIKTGAPLVYELTDDLEVID--KYYL------      228
sp|P30798|GPMA_ZYMMO                  VLIGAHGNSLRALVKHLSKLSDEEIVKFELPTGQPLVYELNDDLTPKD--RYFLNER---      228
tr|J7M1Q4|J7M1Q4_STRP1                VFVGAHGNSIRALVKHIKGLSDDEIMDVEIPNFPPLVFELDEKLNIVK--EYYLGGE---      231
sp|B5XM69|GPMA_STRPZ                  VFVGAHGNSIRALVKHIKGLSDDEIMDVEIPNFPPLVFELDEKLNIVK--EYYLGGE---      231
sp|Q04JB4|GPMA_STRP2                  VFVGAHGNSIRALVKHIKGLSDDEIMDVEIPNFPPLVFEFDEKLNVVS--EYYLGK----      230
tr|A0A0E2QGW3|A0A0E2QGW3_STRTR        VFVGAHGNSIRALVKHIKKLSDDEIMDVEIPNFPPLVFEFDEKLNVVS--EYYLGK----      230
sp|Q839H4|GPMA_ENTFA                  VLVAAHGNSLRALAKHIEGISDEDIMDLEIPTGKPLVYELNDDLTVKE--KYYL------      228
sp|Q8Y571|GPMA_LISMO                  VVIAAHGNSLRALVKFLEGISDDEIMDLEIPTGVPLVYELNADLKPVN--KYYLDK----      229
                                      *.:.*****:*** *.:. :*: :*   :: .  ** :*:  .:        :*

tr|L8EU38|L8EU38_STRR1                KAAIEAVKNQGKKK--          253
tr|A0A0E7UZS0|A0A0E7UZS0_BORPT        EAAMAAVAAQGKAKKD          250
sp|P62707|GPMA_ECOLI                  AAKAAAVANQGKAK--          250
sp|Q2FE81|GPMA_STAA3                  ----------------          228
sp|P30798|GPMA_ZYMMO                  ----------------          228
tr|J7M1Q4|J7M1Q4_STRP1                ----------------          231
sp|B5XM69|GPMA_STRPZ                  ----------------          231
sp|Q04JB4|GPMA_STRP2                  ----------------          230
tr|A0A0E2QGW3|A0A0E2QGW3_STRTR        ----------------          230
sp|Q839H4|GPMA_ENTFA                  ----------------          228
sp|Q8Y571|GPMA_LISMO                  ----------------          229
```

Figure S5. Protein sequence alignment of 2,3-bisphosphoglycerate-dependent phosphoglycerate mutase (GpmA) indicates conserved pgK sites. PgK sites that have been identified in *S. rimosus* (STRR1) [45], *B. pertussis* (BORPT) [49], *E. coli* (ECOLI) [46], *S. aureus* (STAA3) [39], *Z. mobilis* (ZYMMO) [48], *S. pyogenes* M1 (STRP1) [35], *S. pyogenes* M49 (STRPZ) [this work], *S. pneumoniae* (STRP2) [37], *S. thermophilus* (STRTR) [42], *E. faecalis* (ENTFA) [43], and *L. monocytogenes* (LISMO) [35] are highlighted in red.

```
sp|P0A6P9|ENO_ECOLI               MSKIVKIIGREIIDSRGNPTVEAEVHLEGGFVGMAAAPSGASTGSREALELRDGDKSRFL      60
sp|P33675|ENO_ZYMMO               MTAIVSIHGRQVVDSRGNPTVEVDVTLEDGSFGRAAVPSGASTGVHEAVELRDGDKTRWG      60
tr|A0A0E8CMF3|A0A0E8CMF3_BORPT    MSAIVDIIGREILDSRGNPTVECDVLLESGAMGRASVPSGASTGSREAIELRDGDKGRYL      60
tr|A0A8A1UL70|A0A8A1UL70_STRR1    MPSIDVVVAREILDSRGNPTVEVEVGLDDGSTGRAAVPSGASTGAFEAIELRDGDPNRYL      60
sp|Q181T5|ENO_CLOD6               MSVIELVYAREVLDSRGNPTVEVEVVLEDGAMGRAIVPSGASTGAFEAVELRDGDKGRYL      60
sp|Q2FIL7|ENO_STAA3               MPIITDVYAREVLDSRGNPTVEVEVLTESGAFGRALVPSGASTGEHEAVELRDGDKSRYL      60
sp|Q04KG2|ENO_STRP2               MSIITDVYAREVLDSRGNPTLEVEVYTESGAFGRGMVPSGASTGEHEAVELRDGDKSRYG      60
tr|J7M7A0|J7M7A0_STRP1            MSIITDVYAREVLDSRGNPTLEVEVYTESGAFGRGMVPSGASTGEHEAVELRDGDKSRYL      60
sp|B5XKM7|ENO_STRPZ               MSIITDVYAREVLDSRGNPTLEVEVYTESGAFGRGMVPSGASTGEHEAVELRDGDKSRYL      60
                                  *  *  : .*:::*******:* :*  :.*  * . .*******  **:******  *:

sp|P0A6P9|ENO_ECOLI               GKGVTKAVAAVNGPIAQALIG--KDAKDQAGIDKIMIDLDGTENKSKFGANAILAVSLAN      118
sp|P33675|ENO_ZYMMO               GKGVTKAVHAVNNEIANAIIG--LEAEDQELIDQTMIKLDGTPNKGKFGANAILGVSLAV      118
tr|A0A0E8CMF3|A0A0E8CMF3_BORPT    GKGVLRAVENLNTEISEALMG--LDAQEQTFVDRTLIELDGTDSKERLGANAMLAASMAV      118
tr|A0A8A1UL70|A0A8A1UL70_STRR1    GKGVEKAVLAVIEQIGPELVG--YDATEQRLIDQAMFDLDATDNKGSLGANAILGVSLAV      118
sp|Q181T5|ENO_CLOD6               GKGVETAVANVNEIIAPEIEG--MDAFDQPAIDAIMIELDGTPNKGKLGANAILGVSMAV      118
sp|Q2FIL7|ENO_STAA3               GKGVTKAVENVNEIIAPEIIEGEFSVLDQVSIDKMMIALDGTPNKGKLGANAILGVSIAV      120
sp|Q04KG2|ENO_STRP2               GLGTQKAVDNVNNIIAEAIIG--YDVRDQQAIDRAMIALDGTPNKGKLGANAILGVSIAV      118
tr|J7M7A0|J7M7A0_STRP1            GLGTQKAVDNVNNIIAEAIIG--YDVRDQQAIDRAMIALDGTPNKGKLGANAILGVSIAV      118
sp|B5XKM7|ENO_STRPZ               GLGTQKAVDNVNNIIAEAIIG--YDVRDQQAIDRAMIALDGTPNKGKLGANAILGVSIAV      118
                                  * *.  **  :   *.  :      .. :*  :*  :: **.* .*  :****:*..*:*

sp|P0A6P9|ENO_ECOLI               AKAAAAAKGMPLYEHIAELNGTPGKYSMPVPMMNIINGGEHADNNVDIQEFMIQPVGAKT      178
sp|P33675|ENO_ZYMMO               AKAAAEARGLPLYRYVG----GTAAHVLPVPMMNIVNGGMHADNPIDFQEFMIAPVGASS      174
tr|A0A0E8CMF3|A0A0E8CMF3_BORPT    ARAAADESGLSLYRYFG----GSGPMSMPVPMMNVINGGAHANNTLDLQELMILPVGAAS      174
tr|A0A8A1UL70|A0A8A1UL70_STRR1    AHAASEASDLPLFRYLG----GPNAHVLPVPMMNILNGGSHADSNVDIQEFMIAPIGAES      174
sp|Q181T5|ENO_CLOD6               ARAAADEIGLPLFQYLG----GVNAKQLPVPMMNILNGGEHADNNVDVQEFMILPVGACC      174
sp|Q2FIL7|ENO_STAA3               ARAAADLLGQPLYKYLG----GFNGKQLPVPMMNIVNGGSHSDAPIAFQEFMILPVGATT      176
sp|Q04KG2|ENO_STRP2               ARAAADYLEIPLYSYLG----GFNTKVLPTPMMNIINGGSHSDAPIAFQEFMILPVGAPT      174
tr|J7M7A0|J7M7A0_STRP1            ARAAADYLEVPLYTYLG----GFNTKVLPTPMMNIINGGSHSDAPIAFQEFMIMPVGAPT      174
sp|B5XKM7|ENO_STRPZ               ARAAADYLEVPLYTYLG----GFNTKVLPTPMMNIINGGSHSDAPIAFQEFMIMPVGAPT      174
                                  *:**:      *: :..          :*.****::*** *::  : .**:** *:**

sp|P0A6P9|ENO_ECOLI               VKEAIRMGSEVFHHLAKVLKAKGMNTAVGDEGGYAPNLGSNAEALAVIAEAVKAAGYELG      238
sp|P33675|ENO_ZYMMO               INEAVRIGTEVFHTLKKELSAKGMNTNVGDEGGFAPSLDSASSALDFIVDSISKAGYKPG      234
tr|A0A0E8CMF3|A0A0E8CMF3_BORPT    FREALRWGAEVFHMLKKLIHDQGMSTAVGDEGGFAPNVASHEAAIQLILKAITEAGYEPG      234
tr|A0A8A1UL70|A0A8A1UL70_STRR1    FSEALRWGAEVYHTLKKVLKGKGLATGLGDEGGFAPNLGSNREALDLILEAIKEAGYTPG      234
sp|Q181T5|ENO_CLOD6               FKEGLRMGAEVFHSLKKVLGEKGLACGVGDEGGFAPNLGSNREALELIVEAITKAGYKPG      234
sp|Q2FIL7|ENO_STAA3               FKESLRWGTEIFHNLKSILSKRGLETAVGDEGGFAPKFEGTEDAVETIIQAIEAAGYKPG      236
sp|Q04KG2|ENO_STRP2               FKEALRYGAEIFHALKKILKSRGLETAVGDEGGFAPRFEGTEDGVETILAAIEAAGYVPG      234
tr|J7M7A0|J7M7A0_STRP1            FKEGLRWGAEVFHALKKILKERGLVTAVGDEGGFAPKFEGTEDGVETILKAIEAAGYEAG      234
sp|B5XKM7|ENO_STRPZ               FKEGLRWGAEVFHALKKILKERGLVTAVGDEGGFAPKFEGTEDGVETILKAIEAAGYEAG      234
                                  . *.:* *:*::* * . :  :*:   :*****:** . .   .:  *  ::  ***  *

sp|P0A6P9|ENO_ECOLI               K-DITLAMDCAASEFYKD--GKYVL---AGEGNKAFTSEEFTHFLEELTKQYPIVSIEDG      292
sp|P33675|ENO_ZYMMO               E-DVFIALDAASSEFYNKDQNIYDLKG----EGRKLTSAQLVDYYVELCGKYPIYSIEDG      289
tr|A0A0E8CMF3|A0A0E8CMF3_BORPT    T-QIALGLDCASSEFYRD--GKYTL---AGEGGVSLSSQEFANLLATWCDKYPIISIEDG      288
tr|A0A8A1UL70|A0A8A1UL70_STRR1    Q-DIALALDVAASEFYKD--GVYEF------EGQQRTAEQMTAYYAELVDAYPLVSIEDP      285
sp|Q181T5|ENO_CLOD6               E-DVMLGLDVAATEMYNKETKKYVLAG----EGKELTAAEMVALYEDWSNNFPIITIEDG      289
sp|Q2FIL7|ENO_STAA3               E-EVFLGFDCASSEFYEN--GVYDYSKFEGEHGAKRTAAEQVDYLEQLVDKYPIITIEDG      293
sp|Q04KG2|ENO_STRP2               K-DVFLGFDCASSEFYDKERKVYDYTKFEGEGAAVRTSAEQIDYLEELVNKYPIITIEDG      293
tr|J7M7A0|J7M7A0_STRP1            ENGIMIGFDCASSEFYDKERKVYDYTKFEGEGAAVRTSAEQVDYLEELVNKYPIITIEDG      294
sp|B5XKM7|ENO_STRPZ               ENGIMIGFDCASSEFYDKERKVYDYTKFEGEGAAVRTSAEQVDYLEELVNKYPIITIEDG      294
                                     : :.:* *::*:* .    *             :: :           :*: :***

sp|P0A6P9|ENO_ECOLI               LDESDWDGFAYQTKVLGDKIQLVGDDLFVTNTKILKEGIEKGIANSILIKFNQIGSLTET      352
sp|P33675|ENO_ZYMMO               LAEDDFEGWKILTEKLGDKVQLVGDDLFVTNVKRLSDGIERGIANSLLVKFNQIGSLSET      349
tr|A0A0E8CMF3|A0A0E8CMF3_BORPT    MAENDWDGWKLLTDQLGKKVQLVGDDLFVTNTRILREGIQKGVANSILIKINQIGTLTET      348
tr|A0A8A1UL70|A0A8A1UL70_STRR1    LFEDDWDGWKVITDRLGTKVQLVGDDLFVTNPERLARGIEEGSANALLVKVNQIGSLTET      345
sp|Q181T5|ENO_CLOD6               LDEEDWDGWKLLTEKLGNKLQLVGDDLFVTNTERLEKGIENGVANSILVKVNQIGTITET      349
sp|Q2FIL7|ENO_STAA3               MDENDWDGWKQLTERIGDRVQLVGDDLFVTNTEILAKGIENGIGNSILIKVNQIGTLTET      353
sp|Q04KG2|ENO_STRP2               MDENDWDGWKALTERLGKKVQLVGDDFFVTNTDYLARGIQEGAANSILIKVNQIGTLTET      353
tr|J7M7A0|J7M7A0_STRP1            MDENDWDGWKVLTERLGKRVQLVGDDFFVTNTEYLARGIKENAANSILIKVNQIGTLTET      354
sp|B5XKM7|ENO_STRPZ               MDENDWDGWKVLTERLGKRVQLVGDDFFVTNTEYLARGIKENAANSILIKVNQIGTLTET      354
                                  : *.*::*:   *. :* ::******:****   *  **:.. .*::*:*.****:::**

sp|P0A6P9|ENO_ECOLI               LAAIKMAKDAGYTAVISHRSGETEDATIADLAVGTAAGQIKTGSMSRSDRVAKYNQLIRI      412
sp|P33675|ENO_ZYMMO               LAAVNMANDASYTAVMSHRSGETEDTTIADLAVATNCGQIKTGSLCRSERIAKYNQLMRI      409
tr|A0A0E8CMF3|A0A0E8CMF3_BORPT    FAAIEMAKRAGYTAVVSHRSGETEDSTIADIAVATNAMQIKTGSLSRSDRMAKYNQLLRI      408
tr|A0A8A1UL70|A0A8A1UL70_STRR1    LDAVELAQRNGFKCMMSHRSGETEDVTIADLAVATNCGQIKTGAPARSERVAKYNQLLRI      405
sp|Q181T5|ENO_CLOD6               LDAIEMAKRAGYTAVISHRSGETEDSTIADLAVAVNAGQIKTGAPSRTDRVAKYNQLLRI      409
sp|Q2FIL7|ENO_STAA3               FDAIEMAQKAGYTAVVSHRSGETEDTTIADIAVATNAGQIKTGSLSRTDRIAKYNQLLRI      413
sp|Q04KG2|ENO_STRP2               FEAIEMAKEAGYTAVVSHRSGETEDSTIADIAVATNAGQIKTGSLSRTDRIAKYNQLLRI      413
tr|J7M7A0|J7M7A0_STRP1            FEAIEMAKEAGYTAVVSHRSGETEDSTIADIAVATNAGQIKTGSLSRTDRIAKYNQLLRI      414
sp|B5XKM7|ENO_STRPZ               FEAIEMAKEAGYTAVVSHRSGETEDSTIADIAVATNAGQIKTGSLSRTDRIAKYNQLLRI      414
                                  : *:::*:   .:..::********* ****:**.. . *****: .*::*:******:**

sp|P0A6P9|ENO_ECOLI               EEALGEKAPYNGRKEIKGQA----      432
sp|P33675|ENO_ZYMMO               EEELGSVAKYAGRSVLRKAK----      429
tr|A0A0E8CMF3|A0A0E8CMF3_BORPT    EEELAEVASYPGLEAFYNLR----      428
tr|A0A8A1UL70|A0A8A1UL70_STRR1    EEILDDAAVYAGRSAFPRFRFADQ      429
sp|Q181T5|ENO_CLOD6               EEMVGEQARYCGLKSFYNLKK---      430
sp|Q2FIL7|ENO_STAA3               EDELFETAKYDGIKSFYNLDK---      434
sp|Q04KG2|ENO_STRP2               EDQLGEVAEYRGLKSFYNLKK---      434
tr|J7M7A0|J7M7A0_STRP1            EDQLGEVAQYKGIKSFYNLKK---      435
sp|B5XKM7|ENO_STRPZ               EDQLGEVAQYKGIKSFYNLKK---      435
                                  *: : . * * *  . :
```

Figure S6. Protein sequence alignment of enolase (Eno) indicates conserved pgK sites. PgK sites that have been identified in *E. coli* (ECOLI) [46], *Z. mobilis* (ZYMMO) [48], *B. pertussis* (BORPT) [49], *S. rimosus* (STRR1) [45], *C. difficile* (CLOD6) [44], *S. aureus* (STAA3) [39], *S. pneumoniae* (STRP2) [36,37], *S. pyogenes* M1 (STRP1) [35], and *S. pyogenes* M49 (STRPZ) [this work], are highlighted in red.

```
tr|A0Q7J2|A0Q7J2_FRATN               --MRVAINGFGRIGRLAFRQMFG--KDNIEIVAINDLTNPEMLAHLLKYDSAQGRFSKAA      56
tr|A0A0H2XHX6|A0A0H2XHX6_STAA3       MAVKVAINGFGRIGRLAFRRIQE--VEGLEVVAVNDLTDDDMLAHLLKYDTMQGRFTGEV      58
tr|Q181T9|Q181T9_CLOD6               -MVKVAINGFGRIGRLALRKMMEQ-QDKFEVVAINDLTDAKMLAHLFKYDTAQGRFNGEI      58
tr|Q833I8|Q833I8_ENTFA               MTVKVGINGFGRIGRLAFRRIQD--VEGIEVVAINDLTDAKMLAHLLKYDTTQGRFNGTV      58
sp|P0C0G7|G3P_STRP1                  MVVKVGINGFGRIGRLAFRRIQN--IEGVEVTRINDLTDPNMLAHLLKYDTTQGRFDGTV      58
tr|A0A0H3BYM9|A0A0H3BYM9_STRPZ       MVVKVGINGFGRIGRLAFRRIQN--IEGVEVTRINDLTDPNMLAHLLKYDTTQGRFDGTV      58
tr|Q8VVB9|Q8VVB9_STRTR               MVVKVGINGFGRIGRLAFRRIQN--VEGVEVTRINDLTDPVMLAHLLKYDTTQGRFDGTV      58
tr|A0A0H2ZNS0|A0A0H2ZNS0_STRP2       MVVKVGINGFGRIGRLAFRRIQN--VEGVEVTRINDLTDPVMLAHLLKYDTTQGRFDGTV      58
sp|P09316|G3P_ZYMMO                  MAVKVAINGFGRIGRLAARAILSRPDSGLELVTINDLGSVEGNAFLFKRDSAHGTYPGTV      60
sp|P0A9B2|G3P1_ECOLI                 MTIKVGINGFGRIGRIVFRAAQKR--SDIEIVAINDLLDADYMAYMLKYDSTHGRFDGTV      58
tr|A0A0H2XHW0|A0A0H2XHW0_STAA3       MSTNIAINGMGRIGRMVLRIALQN--KNLNVVAINASYPPETIAHLINYDTTHGKYNLKV      58
tr|A0A6M4JMC6|A0A6M4JMC6_BACSU       MKVKVAINGFGRIGRMVFRKAMLD--DQIQVVAINASYSAETLAHLIKYDTIHGRYDKEV      58
tr|A0A6M3ZG39|A0A6M3ZG39_BACSU       MAVKVGINGFGRIGRNVFRAALNN--PEVEVVAVNDLTDANMLAHLLQYDSVHGKLDAEV      58
tr|L8EIJ4|L8EIJ4_STRR1               MTIRVGINGFGRIGRNYFRALLEQ-GADIEIVGVNDLTDNATLVHLLKYDTILGRLKQDV      59
                                        .:.***:*****   *          .::. :*        ..::: *:  *

tr|A0Q7J2|A0Q7J2_FRATN               VTVAKESSIVVDGKEIKIYAEKDAANLPWGELNVDVVLECTGFYVSKAKSQAHIDA-GAK      115
tr|A0A0H2XHX6|A0A0H2XHX6_STAA3       --EVVDGGFRVNGKEVKSFSEPDASKLPWKDLNIDVVLECTGFYTDKDKAQAHIEA-GAK      115
tr|Q181T9|Q181T9_CLOD6               --EVKEGAFVVNGKEIKVTAERNPADLPWAELGVDIVLECTGFFTSKDKAEAHIQA-GAK      115
tr|Q833I8|Q833I8_ENTFA               --EVHEGSFNVNGKEIKVLANRNPEELPWGELGVDIVLECTGFFTSKEAAEKHLTA-GAK      115
sp|P0C0G7|G3P_STRP1                  --EVKEGGFEVNGNFIKVSAERDPENIDWATDGVEIVLEATGFFAKKEAAEKHLHANGAK      116
tr|A0A0H3BYM9|A0A0H3BYM9_STRPZ       --EVKEGGFEVNGNFIKVSAERDPENIDWATDGVEIVLEATGFFAKKEAAEKHLHANGAK      116
tr|Q8VVB9|Q8VVB9_STRTR               --ELKDGGFEVNGKFVKVSAERDPEQIDWANDGVEIVLEATGFFTKKVLAEKHLHPGGAK      116
tr|A0A0H2ZNS0|A0A0H2ZNS0_STRP2       --EVKEGGFEVNGKFIKVSAERDPEQIDWATDGVEIVLEATGFFAKKEAAEKHLK-GGAK      115
sp|P09316|G3P_ZYMMO                  --TTEGNDMVIDGKKIVVTAERDPANLPHKKLGVDIVMECTGIFTNTEKASAHLTA-GAK      117
sp|P0A9B2|G3P1_ECOLI                 --EVKDGHLIVNGKKIRVTAERDPANLKWDEVGVDVVAEATGLFLTDETARKHITA-GAK      115
tr|A0A0H2XHW0|A0A0H2XHW0_STAA3       --EPIENGLQVGDHKIKLVADRNPENLPWKELDIDIAIDATGKFNHGDKAIAHIKA-GAK      115
tr|A0A6M4JMC6|A0A6M4JMC6_BACSU       --VAGEDSLIVNGKKVLLLNSRDPKQLPWREYDIDIVVEATGKFNAKDKAMGHIEA-GAK      115
tr|A0A6M3ZG39|A0A6M3ZG39_BACSU       --SVDGNNLVVNGKTIEVSAERDPAKLSWGKQGVEIVVESTGFFTKRADAAKHLEA-GAK      115
tr|L8EIJ4|L8EIJ4_STRR1               --SHTDDTITVGDQTFKTMAERDPANLPWGELGADIVIESTGIFTKKADAEKHIQA-GAK      116
                                           . : :..: .    . :  .:     . ::. :.** :      :  *:   ***

tr|A0Q7J2|A0Q7J2_FRATN               KVVISAPAGNDLPT-VVFGVNHDILSA-DDKIISAASCTTNCLAPMAKALHDLATIESGF      173
tr|A0A0H2XHX6|A0A0H2XHX6_STAA3       KVLISAPATGDLKT-IVFNTNHQELDG-SETVVSGASCTTNSLAPVAKVLNDDFGLVEGL      173
tr|Q181T9|Q181T9_CLOD6               KVVISAPATGDLKT-IVFNTNSDILDG-SETVISGASCTTNCLAPMAKVLNDKYGIEKGL      173
tr|Q833I8|Q833I8_ENTFA               RVVISAPGGNDVPT-IVYNTNHETLTG-EETVISGASCTTNCLAPMAKALHDNFGVVEGL      173
sp|P0C0G7|G3P_STRP1                  KVVITAPGGNDVKT-VVFNTNHDILDG-TETVISGASCTTNCLAPMAKALHDAFGIQKGL      174
tr|A0A0H3BYM9|A0A0H3BYM9_STRPZ       KVVITAPGGNDVKT-VVFNTNHDILDG-TETVISGASCTTNCLAPMAKALHDAFGIQKGL      174
tr|Q8VVB9|Q8VVB9_STRTR               KVVITAPGGNDVKT-IVFNTNHDILDG-TETVISGASCTTNCLAPMAKALNDNFGIVEGL      174
tr|A0A0H2ZNS0|A0A0H2ZNS0_STRP2       KVVITAPGGNDVKT-VVFNTNHDVLDG-TETVISGASCTTNCLAPMAKALQDNFGVVEGL      173
sp|P09316|G3P_ZYMMO                  KVLISAPAKGDVDRTVVYGVNHKDLTA-DDKIVSNASCTTNCLAPVLHVLQQKIGIVRGL      176
sp|P0A9B2|G3P1_ECOLI                 KVVMTGPSKDNTPM-FVKGANFDKYA--GQDIVSNASCTTNCLAPLAKVINDNFGIIEGL      172
tr|A0A0H2XHW0|A0A0H2XHW0_STAA3       KVLLTGPSKGGHVQMVVKGVNDNQLDIEAFDIFSNASCTTNCIGPVAKVLNNQFGIVNGL      175
tr|A0A6M4JMC6|A0A6M4JMC6_BACSU       KVILTAPGKNEDVT-IVMGVNEDQFDAERHVIISNASCTTNCLAPVVKVLDEEFGIESGL      174
tr|A0A6M3ZG39|A0A6M3ZG39_BACSU       KVIISAPANEEDIT-IVMGVNEDKYDAANHDVISNASCTTNCLAPFAKVLNDKFGIKRGM      174
tr|L8EIJ4|L8EIJ4_STRR1               KVLISAPAKDEDIT-VVMGVNQDKYDAANHHVISNASCTTNCVAPMAKVLDENFGIVKGL      175
                                     :*:::.*.       .* ..* .         :.* ******.:.*. :.:.:   :  *:

tr|A0Q7J2|A0Q7J2_FRATN               MTTIHAYTGDQNTLDAPHAKNDFRRARAAAVNIVPNSTGAAKAIGLVIPELAGKLDGAAQ      233
tr|A0A0H2XHX6|A0A0H2XHX6_STAA3       MTTIHAYTGDQNTQDAPHRKGDKRRARAAAENIIPNSTGAAKAIGKVIPEIDGKLDGGAQ      233
tr|Q181T9|Q181T9_CLOD6               MTTIHAYTNDQNTLDGPHPKGDLRRARAAAGNIVPNTTGAAKAIGLVIPSLKGKLDGAAQ      233
tr|Q833I8|Q833I8_ENTFA               MTTIHAYTGDQMTLDGPHPKGDFRRARAAAANIVPNSTGAAKAIGLVIPELNGKLDGAAQ      233
sp|P0C0G7|G3P_STRP1                  MTTIHAYTGDQMILDGPHRGGDLRRARAGAANIVPNSTGAAKAIGLVIPELNGKLDGAAQ      234
tr|A0A0H3BYM9|A0A0H3BYM9_STRPZ       MTTIHAYTGDQMILDGPHRGGDLRRARAGAANIVPNSTGAAKAIGLVIPELNGKLDGAAQ      234
tr|Q8VVB9|Q8VVB9_STRTR               MTTIHAYTGDQMILDGPNRGGDLRRARAGAANIVPNSTGAAKAIGLVIPELNGKLDGSAQ      234
tr|A0A0H2ZNS0|A0A0H2ZNS0_STRP2       MTTIHAYTGDQMILDGPHRGGDLRRARAGAANIVPNSTGAAKAIGLVIPELNGKLDGSAQ      233
sp|P09316|G3P_ZYMMO                  MTTVHSFTNDQRILDQI-H-SDLRRARTASASMIPTSTGAARAVALVIPELKGKLDGISI      234
sp|P0A9B2|G3P1_ECOLI                 MTTVHATTATQKTVDGPSH-KDWRGGRGASQNIIPSSTGAAKAVGKVLPELNGKLTGMAF      231
tr|A0A0H2XHW0|A0A0H2XHW0_STAA3       MTTVHAITNDQKNIDNP-H-KDLRRARSCNESIIPTSTGAAKALKEVLPELEGKLHGMAL      233
tr|A0A6M4JMC6|A0A6M4JMC6_BACSU       MTTVHAYTNDQKNIDNP-H-KDLRRARACGESIIPTTTGAAKALSLVLPHLKGKLHGLAL      232
tr|A0A6M3ZG39|A0A6M3ZG39_BACSU       MTTVHSYTNDQQILDLP-H-KDYRRARAAAENIIPTSTGAAKAVSLVLPELKGKLNGGAM      232
tr|L8EIJ4|L8EIJ4_STRR1               MTTVHAYTNDQRILDFP-H-KDLRRARAAAENIIPTTTGAAKATALVLPQLEGKLDGIAM      233
                                     ***:*: *  *   *      * * .*    .::*.:****:*   *:* : *** * :

tr|A0Q7J2|A0Q7J2_FRATN               RVPVATGSLTELVAVVSK-KVTAEDVNAAMKAAAN-----KSFGYTEEELVSSDIIGISE      287
tr|A0A0H2XHX6|A0A0H2XHX6_STAA3       RVPVATGSLTELTVVLEKQDVTVEQVNEAMKNASN-----ESFGYTEDEIVSSDVVGMTY      288
tr|Q181T9|Q181T9_CLOD6               RVPVVTGSITELVCTLGK-NVTVEEINAAMKEASN-----ESFGYTEEMLVSSDIIGISY      287
tr|Q833I8|Q833I8_ENTFA               RVPVATGSLTELVTVLDK-EVTVDEVNAVMEKAAN-----ESYGYNTDEIVSSDIVGMTY      287
sp|P0C0G7|G3P_STRP1                  RVPVPTGSVTELVVTLDK-NVSVDEINAAMKAASN-----DSFGYTEDPIVSSDIVGVSY      288
tr|A0A0H3BYM9|A0A0H3BYM9_STRPZ       RVPVPTGSVTELVVTLDK-NVSVDEINAAMKAASN-----DSFGYTEDPIVSSDIVGVSY      288
tr|Q8VVB9|Q8VVB9_STRTR               RVPVPTGSVTELVAVCEK-NVTVDEVNAAMKAATN-----ESYGYTEDPIVSSDIVGMSY      288
tr|A0A0H2ZNS0|A0A0H2ZNS0_STRP2       RVPTPTGSVTELVAVLEK-NVTVDEVNAAMKAASN-----ESYGYTEDPIVSSDIVGMSY      287
sp|P09316|G3P_ZYMMO                  RVPTPDVSLVDFTFVPQR-DTTAEEINSVLKAAADTGDMTGVLGYTDEPLVSRDFYSDPH      293
sp|P0A9B2|G3P1_ECOLI                 RVPTPNVSVVDLTVRLEK-AATYEQIKAAVKAAA-EGEMKGVLGYTEDDVVSTDFNGEVC      289
tr|A0A0H2XHW0|A0A0H2XHW0_STAA3       RVPTKNVSLVDLVVDLEK-EVTAEEVNQAFENA----GLEGIIEVEHQPLVSVDFNTNPN      288
tr|A0A6M4JMC6|A0A6M4JMC6_BACSU       RVPVPNVSLVDLVVDLKT-DVTAEEVNEAFKRAA-KTSMYGVLDYSDEPLVSTDYNTNPH      290
tr|A0A6M3ZG39|A0A6M3ZG39_BACSU       RVPTPNVSLVDLVAELNQ-EVTAEEVNAALKEAA-EGDLKGILGYSEEPLVSGDYNGNKN      290
tr|L8EIJ4|L8EIJ4_STRR1               RVPVPTGSVTDLVVELER-EVTKDEINAAFQKAA-EGQLKGILEYTEDQIVSSDIVNWPA      291
                                     ***.   *:.::.       .: :::: ..: *               : :** *

tr|A0Q7J2|A0Q7J2_FRATN               GSLFDATQTKVTSLGDKSLVKVVSWYDNEMSYTNQMVRVVEYFGAL----------      333
tr|A0A0H2XHX6|A0A0H2XHX6_STAA3       GSLFDATQTRVMSVGDRQLVKVAAWYDNEMSYTAQLVRTLAYLAELSK--------      336
tr|Q181T9|Q181T9_CLOD6               GSLFDATQTKVMEVDGKQLVKVVSWYDNEMSYTSQLIRTLGYFAQLAK--------      335
tr|Q833I8|Q833I8_ENTFA               GSLFDATQTKVMTVGDKQLVKTVAWYDNEMSYTAQLVRTLEYFANL----------      333
sp|P0C0G7|G3P_STRP1                  GSLFDATQTKVMEVDGSQLVKVVSWYDNEMSYTAQLVRTLEYFAKIAK--------      336
tr|A0A0H3BYM9|A0A0H3BYM9_STRPZ       GSLFDATQTKVMEVDGSQLVKVVSWYDNEMSYTAQLVRTLEYFAKIAK--------      336
tr|Q8VVB9|Q8VVB9_STRTR               GSLFDATQTKVLDVDGKQLVKVVSWYDNEMSYTSQLVRTLEYFAKIAK--------      336
tr|A0A0H2ZNS0|A0A0H2ZNS0_STRP2       GSLFDATQTKVLDVDGKQLVKVVSWYDNEMSYTAQLVRTLEYFAKIAK--------      335
sp|P09316|G3P_ZYMMO                  SSTVDSRETAVLE---GKLARVVAWYDNEWGFSNRMVDTAAQMAKTL---------      337
sp|P0A9B2|G3P1_ECOLI                 TSVFDAKAGIALN---DNFVKLVSWYDNETGYSNKVLDLIAHISK-----------      331
tr|A0A0H2XHW0|A0A0H2XHW0_STAA3       SAIIDAKSTMVMS---GNKVKVIAWYDNEWGYSNRVVDVAEQIGALLTSKETVSAS      341
tr|A0A6M4JMC6|A0A6M4JMC6_BACSU       SAVIDGLTTMVME---DRKVKVLAWYDNEWGYSCRVVDLIRHVAARMKHPSAV---      340
tr|A0A6M3ZG39|A0A6M3ZG39_BACSU       SSTIDALSTMVME---GSMVKVISWYDNESGYSNRVVDLAAYIAKKGL--------      335
tr|L8EIJ4|L8EIJ4_STRR1               SCTFDSSLT-MAQ---GKSVKIIGWYDNEWGYSNRLVDLTVFVGGQL---------      334
                                      . .*.            .:  .***** .:: :::     ..
```

Figure S7. Protein sequence alignment of glyceraldehyde-3-phosphate dehydrogenase (GAPDH) indicates conserved pgK sites. PgK sites that have been identified in *F. novicida* (FRATN) [47], *S. aureus* (STAA3) [39], *C. difficile* (CLOD6) [44], *E. faecalis* (ENTFA) [43], *S. pyogenes* M1 (STRP1) [35], *S. pyogenes* M49 (STRPZ) [this work], *S. thermophilus* (STRTR) [42], *S. pneumoniae* (STRP2) [36,37], *Z. mobilis* (ZYMMO) [48], *E. coli* (ECOLI) [46], *B. subtilis* (BACSU) [38,39,35], and *S. rimosus* (STRR1) [45], are highlighted in red.

```
sp|P0A799|PGK_ECOLI                 ---MSVIKMTD-LDLAGKRVFIRADLNVPVKDGKVTSDARIRASLPTIELALKQGAKVMV      56
sp|P09404|PGK_ZYMMO                 ---MAFRTLDDIGDVKGKRVLVREDLNVPMDGDRVTDDTRLRAAIPTVNELAEKGAKVLI      57
tr|L8EEL5|L8EEL5_STRR1              --MKTIDDLAQ-EGLSGKRVFVRADLNVPLDGTTITDDGRIRAVQPTIAKLAQLGAKVIV      57
sp|Q04LZ5|PGK_STRP2                 ---MAKLTVKD-VDLKGKKVLVRVDFNVPLKDGVITNDNRITAALPTIKYIIEQGGRAIL      56
sp|B5XIF1|PGK_STRPZ                 ---MAKLTVKD-VDLKGKKVLVRVDFNVPLKDGVITNDNRITAALPTIKYIIEQGGRAIL      56
sp|Q8Y4I2|PGK_LISMO                 ---MAKKVVTD-LDLKDKKVLVRVDFNVPMKDGKITNDNRIVAALPTIEYILEQNGKAIL      56
sp|Q2FIM0|PGK_STAA3                 ---MAKKIVSD-LDLKGKTVLVRADFNVPLKDGEITNDNRIVQALPTIQYIIEQGGKIVL      56
tr|A0A6M3ZH21|A0A6M3ZH21_BACSU      ---MNKKTLKD-IDVKGKVVFCRVDFNVPMKDGEVTDDTRIRAALPTIKHLADQGAKVLL      56
sp|Q181T8|PGK_CLOD6                 MSMLNKKTIED-IDVCGKKVLVRCDFNVPLQDGVITDENRLNGALPTIQYLISKGAKVIL      59
                                            : :  .: .* *: * *:***:..  :*.: *:    **:    . ..: ::

sp|P0A799|PGK_ECOLI                 TSHLGRPTEGEYNEEFSLLPVVNYLKDKLSNPVRLVKDY--------LDGVDVAEGELVV      108
sp|P09404|PGK_ZYMMO                 LAHFGRPKGQP-NPEMSLARIKDALAGVLGRPVHFI--NDIKGEAAAKAVDALNPGAVAL      114
tr|L8EEL5|L8EEL5_STRR1              ASHLGRPKGAP-DPAFSLRPAAERLGELVGREVAFA--TDTVGESARSVVAGLGEGEVAV      114
sp|Q04LZ5|PGK_STRP2                 FSHLGRVKEESDKAGKSLAPVAADLAAKLGQDVVFP--GVTRGAELEAAINALEDGQVLL      114
sp|B5XIF1|PGK_STRPZ                 FSHLGRVKEEADKEGKSLAPVAADLAAKLGQDVVFP--GVTRGAKLEEAINALEDGQVLL      114
sp|Q8Y4I2|PGK_LISMO                 FSHLGKVKTEEDKEGKSLRPVAVRLSELLGKEVKFV--PTTRGPELEKAIDELKDGEVLL      114
sp|Q2FIM0|PGK_STAA3                 FSHLGKVKEESDKAKLTLRPVAEDLSKKLDKEVVFV--PETRGEKLEAAIKDLKEGDVLL      114
tr|A0A6M3ZH21|A0A6M3ZH21_BACSU      ASHLGRPKGEV-VEELRLTPVAARLGELLGKEVKKA--DEAYGDAVKAQISEMKDGDVLV      113
sp|Q181T8|PGK_CLOD6                 CSHLGKPKGEA-KPELSLAPVAKRLSEMLGKEVVFAADDNVVGENAKKATEKMENGDVVL      118
                                     :*:*: .         *      *   :.. *                   :  * : :

sp|P0A799|PGK_ECOLI                 LENVRFN----KGEKKDDETLSKKYAALCD-VFVMDAFGTAHRAQASTHGIGKFADVACA      163
sp|P09404|PGK_ZYMMO                 LENTRFY----AGEEKNDPALAAEVAKLGD-FYVNDAFSAAHRAHVSTEGLAHKLPA-FA      168
tr|L8EEL5|L8EEL5_STRR1              LENLRFNPGETSKDDAERGAFADQLAELAD-VYVGDGFGAVHRKHASVYDLPARLPH-AA      172
sp|Q04LZ5|PGK_STRP2                 VENTRYEDVDGKKESKNDPELGKYWASLGDGIFVNDAFGTAHRAHASNVGISANVEKAVA      174
sp|B5XIF1|PGK_STRPZ                 VENTRFEDVDGKKESKNDEELGKYWASLGDGIFVNDAFGTAHRAHASNVGISANVEKAVA      174
sp|Q8Y4I2|PGK_LISMO                 FENTRFEDIDGKKESKNDPELGKYWASLGD-VFVNDAFGTAHRAHASNVGIASNLES-AA      172
sp|Q2FIM0|PGK_STAA3                 VENTRYEDLDGKKESKNDPELGKYWASLGD-VFVNDAFGTAHREHASNVGISTHLET-AA      172
tr|A0A6M3ZH21|A0A6M3ZH21_BACSU      LENVRFY----PGEEKNDPELAKAFAELAD-VYVNDAFGAAHRAHASTAGIAEHLP-AVA      167
sp|Q181T8|PGK_CLOD6                 LENTRYR----KEETKNEENFSKELASLAE-IFVNDAFGTAHRAHCSTVGAGEFLQERVC      173
                                    .** *:       :  :   :.   * * : .:* *.*.:.** : *  .         .

sp|P0A799|PGK_ECOLI                 GPLLAAELDALGKALKEPARPMVAIVGGSKVSTKLTVLDSLSKIADQLIVGGGIANTFIA      223
sp|P09404|PGK_ZYMMO                 GRAMQKELEALEAALGKPTHPVAAVVGGAKVSTKLDVLTNLVSKVDHLIIGGGMANTFLA      228
tr|L8EEL5|L8EEL5_STRR1              GDLIATEVTVLKKLTEDVKRPYVVVLGGAKVSDKLAVIDELLGKADRLLIGGGMAYTFLK      232
sp|Q04LZ5|PGK_STRP2                 GFLLENEIAYIQEAVETPERPFVAILGGSKVSDKIGVIENLLEKADKVLIGGGMTYTFYK      234
sp|B5XIF1|PGK_STRPZ                 GFLLENEIAYIQEAVETPERPFVAILGGSKVSDKIGVIENLLEKADKVLIGGGMTYTFYK      234
sp|Q8Y4I2|PGK_LISMO                 GFLMEKEIKFIGGVVDNPARPLVAILGGAKVSDKIGVIENLLTKADKVLVGGGMTFTFMA      232
sp|Q2FIM0|PGK_STAA3                 GFLMDKEIKFIGGVVNDPHKPVVAILGGAKVSDKINVIKNLVNIADKIIIGGGMAYTFLK      232
tr|A0A6M3ZH21|A0A6M3ZH21_BACSU      GFLMEKELDVLGKAVSNPDRPFTAIIGGAKVKDKIGVIESLLDKVDNLIIGGGLAYTFVK      227
sp|Q181T8|PGK_CLOD6                 GYLIQKELKFLGEAVANPVRPFTAILGGAKVSDKLAVINELLEKVDNLIIGGGMAYTFLK      233
                                    *  :  *:  :        :* ..::**:**. *: *: .*   .*.:::***:: **

sp|P0A799|PGK_ECOLI                 AQGHDVGKSLYEADLVDEAKRLLT-----TCNIPVPSDVRVATEFSET-----APATLKS      273
sp|P09404|PGK_ZYMMO                 AQGVDVGKSLCEHELKDTVKGIFAAAEKTGCKIHLPSDVVVAKEFKAN-----PPIRTIP      283
tr|L8EEL5|L8EEL5_STRR1              AKGYEVGISLLQEDQIPAVTEYMERAEKNGVELVLPVDILASADFPDLKTKAPADFVTVD      292
sp|Q04LZ5|PGK_STRP2                 AQGIEIGNSLVEEDKLDVAKALLEKANG---KLILPVDSKEANAFAGY-----TEVRDTE      286
sp|B5XIF1|PGK_STRPZ                 AQGIEIGNSLVEEDKLDVAKDLLEKSNG---KLILPVDSKEANAFAGY-----TEVRDTE      286
sp|Q8Y4I2|PGK_LISMO                 AQGQEIGKSLLEADKVELAKGLLEKAGD---KLVLPVDAVVSKEFSND-----APFHTVS      284
sp|Q2FIM0|PGK_STAA3                 AQGKEIGISLLEEDKIDFAKDLLEKHGD---KIVLPVDTKVAKEFSND-----AKITVVP      284
tr|A0A6M3ZH21|A0A6M3ZH21_BACSU      ALGYEVGKSLLEEDKIELAKSFMDRAKEKGVNFYMPEDVLVADDFSND-----ANVKIVP      282
sp|Q181T8|PGK_CLOD6                 AQGYEVGTSLLEIDKVEYAKEMMEKAKNKGVNLLLPVDVVMADHFAPD-----ATPIVTE      288
                                    * * ::* ** : :    ..  :        :: :* *   :  *

sp|P0A799|PGK_ECOLI                 VNDVKADEQILDIGDASAQELAEILKNAKTILWNGPVGVFEFPNFRKGTEIVANAIAD--      331
sp|P09404|PGK_ZYMMO                 VSDVAADEMILDVGPKAVAALTEVLKASKTLVWNGPLGAFEIEPFDKATVALAKEAAALT      343
tr|L8EEL5|L8EEL5_STRR1              ADQIPSDKEGLDIGPKTRELFASKIADAETVFWNGPVGVAEHPDYAGGTTAIARALLN--      350
sp|Q04LZ5|PGK_STRP2                 GEAVSEGFLGLDIGPKSIAKFDEALTGAKTVVWNGPMGVFENPDFQAGTIGVMDAIVK--      344
sp|B5XIF1|PGK_STRPZ                 GEAVSEGFLGLDIGPKSIAEFDQALTGAKTVVWNGPMGVFENPDFQAGTIGVMDAIVK--      344
sp|Q8Y4I2|PGK_LISMO                 ADSIPADEMGLDIGQATIDLFTKELQGAKTVVWNGPMGVFELSNFAKGTIGVCEAIAN--      342
sp|Q2FIM0|PGK_STAA3                 SDSIPADQEGMDIGPNTVKLFADELEGAHTVVWNGPMGVFEFSNFAQGTIGVCKAIAN--      342
tr|A0A6M3ZH21|A0A6M3ZH21_BACSU      ISEIPSDLEAIDIGTKTRETYADVIKNSKLVVWNGPMGVFEIDLFAQGTKAVAEALAE--      340
sp|Q181T8|PGK_CLOD6                 DANVKEDYMGLDMGPKTIANFVKTIKESKTVVWNGPMGVFEFENFANGTLSVARAMAE--      346
                                       :  .   :*:*  :     . :  :. :.****:*. *   :  .*  :

sp|P0A799|PGK_ECOLI                 --SEAFSIAGGGDTLAAIDLFGIA-DKISYISTGGGAFLEFVEGKVLPAVAMLEERAKK        387
sp|P09404|PGK_ZYMMO                 KAGSLISVAGGGDTVAALNHAGVA-KDFSFVSTAGGAFLEWMEGKELPGVKALEA----        397
tr|L8EEL5|L8EEL5_STRR1              -C-DGFTVVGGGDSAAAVRSLGFDENAFGHISTGGGASLEYLEGKTLPGLAALED----        403
sp|Q04LZ5|PGK_STRP2                 -QPGVKSIIGGGDSAAAAINLGRA-DKFSWISTGGGASMELLEGKVLPGLAALTEK---        398
sp|B5XIF1|PGK_STRPZ                 -QPGVKSIIGGGDSAAAAINLGRA-DKFSWISTGGGASMELLEGKVLPGLAALTEK---        398
sp|Q8Y4I2|PGK_LISMO                 -LTDATTIIGGGDSAAAAMDLGFA-DKFTHISTGGGASLEYLEGKELPGVASISDK---        396
sp|Q2FIM0|PGK_STAA3                 -LKDAITIIGGGDSAAAAISLGFE-NDFTHISTGGGASLEYLEGKELPGIKAINNK---        396
tr|A0A6M3ZH21|A0A6M3ZH21_BACSU      -AKDTYSVIGGGDSAAAVEKFGLA-DKMSHISTGGGASLEFMEGKELPGVAALNDK---        394
sp|Q181T8|PGK_CLOD6                 -LTDATTVIGGGDSAAAVNQLGFG-DKMTHVSTGGGASLEFLEGKELPGIAALDNK---        400
                                          :: ****: **    *    . :  :**.*** :* :*** **.:  :
```

Figure S8. Protein sequence alignment of phosphoglycerate kinase (Pgk) indicates conserved pgK sites. PgK sites that have been identified in *E. coli* (ECOLI) [46], *Z. mobilis* (ZYMMO) [48], *S. rimosus* (STRR1) [45], *S. pneumoniae* (STRP2) [37], *S. pyogenes* M49 (STRPZ) [this work], *L. monocytogenes* (LISMO) [35], *S. aureus* (STAA3) [39], *B. subtilis* (BACSU) [38,39], and *C. difficile* (CLOD6) [44] and are highlighted in red.

```
tr|Q181T6|Q181T6_CLOD6              -MRKPIIAGNWKMHKTIKEALEFVNEIKDKV-NSDKVEAVICAPFTLLKDLK---EATKG      55
tr|J7M1C2|J7M1C2_STRP1              MSRKPIIAGNWKMNKNPQEAKAFVEAVASKLPSTDLVDVAVAAPAVDLVTT---IEAAKD      57
sp|B5XKI2|TPIS_STRPZ                MSRKPIIAGNWKMNKNPQEAKAFVEAVASKLPSTDLVDVAVAAPAVDLVTT---IEAAKD      57
tr|A0A6M4JPA8|A0A6M4JPA8_BACSU      -MRKPIIAGNWKMNKTLGEAVSFVEEVKSSIPAADKAEAVVCAPALFLEKLASAVK---G      56
sp|Q2FIL9|TPIS_STAA3                -MRTPIIAGNWKMNKTVQEAKDFVNAL-PTLPDSKEVESVICAPAIQLDALTTAVKEGKA      58
                                      *.*********:*.  **   **: :   .:   :. .: .:.**    *       :

tr|Q181T6|Q181T6_CLOD6              TNIKIGAQNMHFEEKGAFTGEVSPLMLKEIDMDYVVIGHSERRQYFNETDETVNKKVLKA      115
tr|J7M1C2|J7M1C2_STRP1              SVLKVAAQNCYFENTGAFTGETSPKVLAEMGADYVVIGHSERRDYFHETDEDINKKAKAI      117
sp|B5XKI2|TPIS_STRPZ                SVLKVAAQNCYFENTGAFTGETSPKVLAEMGADYVVIGHSERRDYFHETDEDINKKAKAI      117
tr|A0A6M4JPA8|A0A6M4JPA8_BACSU      TDLKVGAQNMHFEESGAFTGEISPVALKDLGVDYCVIGHSERREMFAETDETVNKKAHAA      116
sp|Q2FIL9|TPIS_STAA3                QGLEIGAQNTYFEDNGAFTGETSPVALADLGVKYVVIGHSERRELFHETDEEINKKAHAI      118
                                      :::.*** :**:.****** **   * ::.  .* ********: * **** :***.

tr|Q181T6|Q181T6_CLOD6              LEVGIDPILCVGETLEQREAGKTKDVCKVQVEKALENVLKDDLAKVVVAYEPIWAIGTGK      175
tr|J7M1C2|J7M1C2_STRP1              FANGLTPIVCCGESLETYEAGKAVEFVGAQVSAALAGLSAEQVASLVLAYEPIWAIGTGK      177
sp|B5XKI2|TPIS_STRPZ                FANGLTPIVCCGESLETYEAGKAVEFVGAQVSAALAGLSAEQVASLVLAYEPIWAIGTGK      177
tr|A0A6M4JPA8|A0A6M4JPA8_BACSU      FKHGIVPIICVGETLEEREAGKTNDLVADQVKKGLAGLSEEQVAASVIAYEPIWAIGTGK      176
sp|Q2FIL9|TPIS_STAA3                FKHGMTPIICVGETDEERESGKANDVVGEQVKKAVAGLSEDQLKSVVIAYEPIWAIGTGK      178
                                    :  *: **:* **: *  *:**: :.   **. .: .:  :::   *:************

tr|Q181T6|Q181T6_CLOD6              TATAEDANDVISYIREVIKGLYG-ELANEVRIQYGGSVKPSNVAEIMGQSDIDGALVGGA      234
tr|J7M1C2|J7M1C2_STRP1              SATQDDAQNMCKAVRDVVAADFGQEVADKVRVQYGGSVKPENVKDYMACPDVDGALVGGA      237
sp|B5XKI2|TPIS_STRPZ                SATQDDAQNMCKAVRDVVAADFGQEVADKVRVQYGGSVKPENVKDYMACPDVDGALVGGA      237
tr|A0A6M4JPA8|A0A6M4JPA8_BACSU      SSTAKDANDVCAHIRKTVAESFSQEAADKLRIQYGGSVKPANIKEYMAESDIDGALVGGA      236
sp|Q2FIL9|TPIS_STAA3                SSTSEDANEMCAFVRQTIADLSSKEVSEATRIQYGGSVKPNNIKEYMAQTDIDGALVGGA      238
                                    ::* .**:::   :*..:     . * ::  *:******** *: : *.  *:********

tr|Q181T6|Q181T6_CLOD6              SLASNDYLDLVNF----          247
tr|J7M1C2|J7M1C2_STRP1              SLEADSFLALLDFLN--          252
sp|B5XKI2|TPIS_STRPZ                SLEADSFLALLDFLN--          252
tr|A0A6M4JPA8|A0A6M4JPA8_BACSU      SLEPQSFVQLLEEGQYE          253
sp|Q2FIL9|TPIS_STAA3                SLKVEDFVQLLEGAK--          253
                                    **  :.:: *::
```

Figure S9. Protein sequence alignment of triosephosphate isomerase (Tpi) indicates conserved pgK sites. PgK sites that have been identified in *C. difficile* (CLOD6) [44], *S. pyogenes* M1 (STRP1) [35], *S. pyogenes* M49 (STRPZ) [this work], *B. subtilis* (BACSU) [38,39], and *S. aureus* (STAA3) [39] and are highlighted in red.

```
sp|P0A8F0|UPP_ECOLI                 -MKIVEVKHPLVKHKLGLMREQDISTKRFRELASEVGSLLTYEATADLETEKVTIEGWNG      59
tr|A0A6N3JAT7|A0A6N3JAT7_FRATN      -MKVVEISHPMVKHKLGLMRAASISTQEFRRLTKEITSLLTYEVTAGFELEKTEILGWQG      59
sp|B5XJZ7|UPP_STRPZ                 MGKCQVISHPLIQHKLSILRRQTTSTKDFRELVNEIAMLMGYEVSRDLPLEDVDIQTPVS      60
sp|Q8Y4B3|UPP_LISMO                 MANVHVINHPLVQHKLTIIRDKNTGTKAFRELVDEVATLMAYEITRDMELEDIQVETPLQ      60
sp|Q2FF16|UPP_STAA3                 MSKVHVFDHPLIQHKLSYIRDVNTGTKEFRELVDEVGMLMAYEVTRDLELQDVDIETPVT      60
                                      :   ..**:::***   :*     .*: **.*..*:   *: ** : .:  :.   :

sp|P0A8F0|UPP_ECOLI                 P-VEIDQIKGKKITVVPILRAGLGMMDGVLENVPSARISVVGMYRNEETLEPVPYFQKLV      118
tr|A0A6N3JAT7|A0A6N3JAT7_FRATN      ENIEIDQIKGKKLTVVPILRAGLGMMDGVFEHVPAAKVSMVGMYRDEKTAKPVAYFAKLC      119
sp|B5XJZ7|UPP_STRPZ                 K-TVQKQLAGKKLAIVPILRAGIGMVDGLLSLVPAAKVGHIGMYRNEETLEPVEYLVKLP      119
sp|Q8Y4B3|UPP_LISMO                 T-TTAKTLTGKKLGIVPILRAGLGMQDGILKLIPAAKVGHVGLYRDHDTLEPVEYFVKLP      119
sp|Q2FF16|UPP_STAA3                 K-MTAKRLAGKKLAIVPILRAGLGMTDGILSLVPAARVGHIGLYRDPETLKAVEYFAKLP      119
                                         . : ***: :*******:** **::. :*:*::. :*:**: .* : * *: **

sp|P0A8F0|UPP_ECOLI                 SNIDERMALIVDPMLATGGSVIATIDLLKKAGCSSIKVLVLVAAPEGIAALEKAHPDVEL      178
tr|A0A6N3JAT7|A0A6N3JAT7_FRATN      DKLDERVALIVDPMLATGGSMIATVSLLKKAGSKDIKIITLVSAPEGIDALAKAHPDVEL      179
sp|B5XJZ7|UPP_STRPZ                 EDINQRQIFLVDPMLATGGSAILAVDSLKKRGAANIKFVCLVAAPEGVKKLQEAHPDIDI      179
sp|Q8Y4B3|UPP_LISMO                 SDVEERLFIVVDPMLATGGSAIMAIDCLKKRGARNMKFMCLVAAPEGVKALQDAHPDVEI      179
sp|Q2FF16|UPP_STAA3                 QDITERQIIVVDPMLATGASAIEAITSLKKRGAKNIRFMCLIAAPEGVEKMHEAHPDVDI      179
                                    ..: :*   ::********.* * ::   *** *. .::.: *::****:  : .****:::

sp|P0A8F0|UPP_ECOLI                 YTASIDQGLNEHGYIIPGLGDAGDKIFGTK          208
tr|A0A6N3JAT7|A0A6N3JAT7_FRATN      YTASIDSHLNDKKYIIPGLGDAGDKIFGTK          209
sp|B5XJZ7|UPP_STRPZ                 FTAALDDHLNDHGYIVPGLGDAGDRLFGPK          209
sp|Q8Y4B3|UPP_LISMO                 YVAGLDEKLDENGYIRPGLGDAGDRLFGTK          209
sp|Q2FF16|UPP_STAA3                 YIAALDEKLNDKAYITPGLGDAGDRLFGTK          209
                                    : *.:*. *::: ** ********::** *
```

Figure S10. Protein sequence alignment of uracil phosphoribosyltransferase (Upp) indicates conserved pgK sites. PgK sites that have been identified in *E. coli* (ECOLI) [46], *F. novicida* (FRATN) [47], *S. pyogenes* M49 (STRPZ) [this work], *L. monocytogenes* (LISMO) [35], and *S. aureus* (STAA3) [39] are highlighted in red.

```
sp|P0AAB6|GALF_ECOLI                ---MTNLKAVIPVAGLGMHMLPATKAIPKEMLPIVDKPMIQYIVDEIVAAGIKEILLVTH      57
tr|Q5NLL9|Q5NLL9_ZYMMO              --MKPVRKAIFPVAGQGTRFLPATKAMPKEMLPVVDRPLIQYAVDEARAAGIEELIFVTG      58
tr|A0A0H2ZMV4|A0A0H2ZMV4_STRP2      -MTSKVRKAVIPAAGLGTRFLPATKALAKEMLPIVDKPTIQFIVEEALKSGIEDILVVTG      59
tr|J7MBC8|J7MBC8_STRP1              MLMTKVRKAIIPAAGLGTRFLPATKALAKEMLPIVDKPTIQFIVEEALKSGIEEILIVTG      60
tr|Q183E9|Q183E9_CLOD6              -MQMTVKKAIIPAAGLGTRFLPATKSQPKEMLPIVDKPTLQYIIEEAIESGIEEILIITG      59
tr|A0A6M3ZL57|A0A6M3ZL57_BACSU      --MKKVRKAIIPAAGLGTRFLPATKAMPKEMLPIVDKPTIQYIIEEAVEAGIEDIIIVTG      58
                                           **::*.** * ::*****:  *****:**:* :*: ::*   :**::::.:*

sp|P0AAB6|GALF_ECOLI                ASKNAVENHFDTSYELESLLEQRVKRQLLAEVQSICPPGVTIMNVRQGEPLGLGHSILCA      117
tr|Q5NLL9|Q5NLL9_ZYMMO              RGKQAIEDYFDIAYELEASLTAKGKTGMLDLLEGTRVAPGRACFIRQQEPLGLGHAVWCA      118
tr|A0A0H2ZMV4|A0A0H2ZMV4_STRP2      KSKRSIEDHFDSNFELEYNLKEKGKTDLLKLVDETT--GMRLHFIRQTHPRGLGDAVLQA      117
tr|J7MBC8|J7MBC8_STRP1              KSKRSIEDHFDSNFELEYNLQAKGKIELLKLVDETT--SINLHFIRQSHPRGLGDAVLQA      118
tr|Q183E9|Q183E9_CLOD6              RNKKSIEDHFDKSVELELELEQKGKTEMLEMVRDISN-MVNIHYIRQKEPKGLGHAIYCA      118
tr|A0A6M3ZL57|A0A6M3ZL57_BACSU      KSKRAIEDHFDYSPELERNLEEKGKTELLEKVKKASN-LADIHYIRQKEPKGLGHAVWCA      117
                                     .*.::*::**   ***  *  : *  :*  :            :** .* ***.::  *

sp|P0AAB6|GALF_ECOLI                RPAIGDNPFVVVLPDVVIDDASADPLRYNLAAMIARFNETGRSQVLAKRMPG-DLSEYSV      176
tr|Q5NLL9|Q5NLL9_ZYMMO              RDLIGDEPFAVLLPDELLWNP----KRPSLVQLVETYNQKGGNVVTVMEVPEEHTHRYGI      174
tr|A0A0H2ZMV4|A0A0H2ZMV4_STRP2      KAFVGNEPFVVMLGDDLMDITDEK-AVPLTKQLMDDYKRTHASTIAVMPVPHDEVSAYGV      176
tr|J7MBC8|J7MBC8_STRP1              KTFVGNEPFVVMLGDDLMDITNPN-VKPLTKQLIDDYEETHAATIAVMRVPHEDVSNYGI      177
tr|Q183E9|Q183E9_CLOD6              KSFIGDEPFAVLLGDDIVDSD-----VPCLKQLIDTYNEYKTTVLGVQKIAKEDTNKYGI      173
tr|A0A6M3ZL57|A0A6M3ZL57_BACSU      RNFIGDEPFAVLLGDDIVQAE-----TPGLRQLMDEYEKTLSSIIGVQQVPEEETHRYGI      172
                                    :  :*::**.*:* ::              ::  ::.     : .  :   .   *.:

sp|P0AAB6|GALF_ECOLI                IQTKEPLDREGKVSRIVEFIEKPDQPQTLDSDIMAVGRYVLSADIWPELERTQPGAWGRI      236
tr|Q5NLL9|Q5NLL9_ZYMMO              VDPGKT---DGEVTEVKGLVEKP---EKAPSRLAMTGRYILQPDIMPLLAQDNRGVGGEI      228
tr|A0A0H2ZMV4|A0A0H2ZMV4_STRP2      IAPQGE--GKDGLYSVETFVEKPA-PEDAPSDLAIIGRYLLTPEIFQILENQAPGAGNEI      233
tr|J7MBC8|J7MBC8_STRP1              IAPQAK--AVKGLYSVDTFVEKPQ-PQDAPSDLAIIGRYLLTPEIFSILEKQEPGAGNEV      234
tr|Q183E9|Q183E9_CLOD6              LDVKHI---EDRVYKVKDMVEKPA-IEEAPSDIAILGRYIITPAIFSILEKQTPGKGGEI      229
tr|A0A6M3ZL57|A0A6M3ZL57_BACSU      IDPLTS---EGRRYQVKNFVEKPP-KGTAPSNLAILGRYVFTPEIFMYLEEQQVGAGGEI      228
                                    :              :  ::***       * :   ***::   *   * .   *  ..:

sp|P0AAB6|GALF_ECOLI                QLTDAIAELAKKQSVDAMLMTGDSYDCGKKMGYMQAFVKYGLRNLKEGAKFRKGIEKLLS      296
tr|Q5NLL9|Q5NLL9_ZYMMO              QLTDSMAQLIGKQPFHACRFGGIRHDCGDKAGFIQANVALALERPDIGPAVRDYLHRQNF      288
tr|A0A0H2ZMV4|A0A0H2ZMV4_STRP2      QLTDAIDTLNKTQRVFAREFTGARYDVGDKFGFMKTSIDYALKHPQVKDDLKNYLIQLGK      293
tr|J7MBC8|J7MBC8_STRP1              QLTDAIDTLNKTQRVFAREFKGKRYDVGDKFGFMKTSLDYALKHPQVKDDLKAYIIQLGK      294
tr|Q183E9|Q183E9_CLOD6              QLTDALQTLGKQEAIYAYNFEGRRYDVGDKIGFLEATIDFALKRENLKDDLMDYMRKKVS      289
tr|A0A6M3ZL57|A0A6M3ZL57_BACSU      QLTDAIQKLNEIQRVFAYDFEGKRYDVGEKLGFITTTLEFAMQDKELRDQLVPFMEGLLN      288
                                    ****::  *   : . * : * :* *.* *:: : :  .:.  .    .   :

sp|P0AAB6|GALF_ECOLI                E------        297
tr|Q5NLL9|Q5NLL9_ZYMMO              -------        288
tr|A0A0H2ZMV4|A0A0H2ZMV4_STRP2      ELTEKE-        299
tr|J7MBC8|J7MBC8_STRP1              ALEKTKP        301
tr|Q183E9|Q183E9_CLOD6              EK-----        291
tr|A0A6M3ZL57|A0A6M3ZL57_BACSU      KEEI---        292
```

Figure S11. Protein sequence alignment of UTP--glucose-1-phosphate uridylyltransferase (UGPase) indicates conserved pgK sites. PgK sites that have been identified in *E. coli* (ECOLI) [46], *Z. mobilis* (ZYMMO) [48], *S. pyogenes* M1 (STRP1) [35], *S. pneumoniae* (STRP2) [37], *C. difficile* (CLOD6) [44], and *B. subtilis* (BACSU) [39] are highlighted in red.

```
tr|L8EUQ4|L8EUQ4_STRR1              MAELTIRPEEIRDALENFVQAYKPDAASREEVGTVSEAGDGIAKVEGLPSAMANELLKFE    60
sp|P0ABB0|ATPA_ECOLI                ---MQLNSTEISELIKQRIAQFNVVS-EAHNEGTIVSVSDGVIRIHGLADCMQGEMISLP    56
tr|A0A6N3JBE3|A0A6N3JBE3_FRATN      ---MQLSPSEISGLIKQRIEKFDNSV-ELKSEGTIVSVADGIVTIYGLNDVAAGEMIKLP    56
sp|Q5NQZ1|ATPA_ZYMMO                ---MEIRAAEISKVIREQIEGFGADA-EVSEVGRVISVGDGIARVYGLDKVEAGEMVTFS    56
sp|Q180W8|ATPA_CLOD6                ---MNLKPEEISSIIKQQIKNYENKV-ELTDTGSVLTVGDGIASVYGLEKAMSGELLEFP    56
tr|A0A6M4JQ33|A0A6M4JQ33_BACSU      ---MSIKAEEISTLIKQQIQNYQSDI-EVQDVGTVIQVGDGIARVHGLDNCMAGELVEFS    56
sp|Q2FF22|ATPA_STAA3                ---MAIKAEEISALLRSQIENYESEM-SVTDVGTVLQIGDGIALIHGLNDVMAGELVEFH    56
                                       : :   **   :.. :  :      .   . * :   .**:  : ** .   .*:: :

tr|L8EUQ4|L8EUQ4_STRR1              DGTLGLALNLEEREIGAVVLGEFSGIEEGQPVRRTGEVLSVAVGEGYLGRVVDPLGNPVD    120
sp|P0ABB0|ATPA_ECOLI                GNRYAIALNLERDSVGAVVMGPYADLAEGMKVKCTGRILEVPVGRGLLGRVVNTLGAPID    116
tr|A0A6N3JBE3|A0A6N3JBE3_FRATN      GDVYGLALNLNTDSVGAVVLGDYEHIKEGDKAYCTGRILEVPVGEALLGRVVDALGNPID    116
sp|Q5NQZ1|ATPA_ZYMMO                SGVQGMALNLEADNVGVVIFGSDLEVGEGDTVRRTGQIVDVPVGPELLGRVVDALGNPID    116
sp|Q180W8|ATPA_CLOD6                GEIYGMALNLEEEVVGAVILGDDSEIKEGDIVKRTGRIVEVPVGEALIGRVVNSLGQPID    116
tr|A0A6M4JQ33|A0A6M4JQ33_BACSU      NGVLGMAQNLEESNVGIVILGPFSEIREGDEVKRTGRIMEVPVGEELIGRIVNPLGQPVD    116
sp|Q2FF22|ATPA_STAA3                NGVLGLAQNLEESNVGVVILGPYTGITEGDEVKRTGRIMEVPVGEELIGRVVNPLGQPID    116
                                    .    .:* **:    :* *::*      : **   .   **.::.* **    :**:*: ** *:*

tr|L8EUQ4|L8EUQ4_STRR1              GLGEIESEGRRALELQAPGVMVRKSVHEPMQTGYKAVDSMVPIGRGQRQLIIGDRQTGKT    180
sp|P0ABB0|ATPA_ECOLI                GKGPLDHDGFSAVEAIAPGVIERQSVDQPVQTGYKAVDSMIPIGRGQRELIIGDRQTGKT    176
tr|A0A6N3JBE3|A0A6N3JBE3_FRATN      GKGEVATDLTSPIEKIAPGVIWRKSVDQALQTGIKSIDSMVPIGRGQRELIIGDRQIGKT    176
sp|Q5NQZ1|ATPA_ZYMMO                GKGPINAKLRRRSEEMAPGIIPRKSVHEAVQTGIKALDALVPVGRGQRELIIGDRQTGKT    176
sp|Q180W8|ATPA_CLOD6                GKGPIAYTKTRPVESEAPGIIDRRSVYEPLQTGIKSIDSMIPIGRGQRELIIGDRQTGKT    176
tr|A0A6M4JQ33|A0A6M4JQ33_BACSU      GLGPILTSKTRPIESPAPGVMDRKSVHEPLQTGIKAIDALIPIGRGQRELIIGDRQTGKT    176
sp|Q2FF22|ATPA_STAA3                GQGPINTTKTRPVEKKATGVMDRKSVDEPLQTGIKAIDALVPIGRGQRELIIGDRQTGKT    176
                                    * *  :          *   * *:: *:** : :*** *::*:::*:*****:******* ***

tr|L8EUQ4|L8EUQ4_STRR1              ALAVDTIINQRDNWRSGDPKKQVRCIYVAVGQKGSTIASVRGALEEAGALEYTTIVAAPA    240
sp|P0ABB0|ATPA_ECOLI                ALAIDAIINQRD--------SGIKCIYVAIGQKASTISNVVRKLEEHGALANTIVVVATA    228
tr|A0A6N3JBE3|A0A6N3JBE3_FRATN      AIAVDTIINQKG--------TGVKCIYVAIGQKASTIANIVRQLEEHGAMEHTIIVAATA    228
sp|Q5NQZ1|ATPA_ZYMMO                AIAIDAFINQKQVNSGSDNTKKLFCIYVAIGQKRSTVAQIVRQLEEMGAMEYSIVVAATA    236
sp|Q180W8|ATPA_CLOD6                SIVIDTILNQKG--------KDVICIYVAIGQKRSTIAQLVSSLEKGGALDYTIVVSATA    228
tr|A0A6M4JQ33|A0A6M4JQ33_BACSU      SVAIDAILNQKD--------QDMICVYVAIGQKESTVRGVVETLRKHGALDYTIVVTASA    228
sp|Q2FF22|ATPA_STAA3                TIAIDTILNQKD--------QGTICIYVAIGQKDSTVRANVEKLRQAGALDYTIVVAASA    228
                                    ::.:*:::**:                *:***:*** **:       *.: **:   : :* * *

tr|L8EUQ4|L8EUQ4_STRR1              SDPAGFKYLAPYTGSAIGQHWMYQGKHVLIIFDDLSKQADAYRAVSLLLRRPPGREAYPG    300
sp|P0ABB0|ATPA_ECOLI                SESAALQYLAPYAGCAMGEYFRDRGEDALIIYDDLSKQAVAYRQISLLLRRPPGREAFPG    288
tr|A0A6N3JBE3|A0A6N3JBE3_FRATN      SDSAALQYIAPYAGCSMGEYFRDRGQDALIVYDDLTKQAWAYRQISLLLRRPPGREAYPG    288
sp|Q5NQZ1|ATPA_ZYMMO                SEPAPLQYLVPYSACSMGEYFRDNKQHALIVYDDLSKQAVAYRQMSLLLRRPPGREAYPG    296
sp|Q180W8|ATPA_CLOD6                SESAPLQYIAPYAGAAMGEEFMYNGKHVLIVYDDLSKQAVAYREMSLLLRRPPGREAYPG    288
tr|A0A6M4JQ33|A0A6M4JQ33_BACSU      SQPAPLLYLAPYAGVTMAEEFMYNGKHVLVVYDDLSKQAAAYRELSLLLRRPPGREAFPG    288
sp|Q2FF22|ATPA_STAA3                SEPSPLLYIAPYSGVTMGEEFMFNGKHVLIVYDDLTKQAAAYRELSLLLRRPPGREAYPG    288
                                    *: : : *:.**:.  ::.: :   . :..*:::***:*** *** :*************:**

tr|L8EUQ4|L8EUQ4_STRR1              DVFYLHSRLLERCAKLSDEL-----------GAGSMTGLPIVETKANDVSAFIPTNVISI    349
sp|P0ABB0|ATPA_ECOLI                DVFYLHSRLLERAARVNAEYVEAFTKGEVKGKTGSLTALPIIETQAGDVSAFVPTNVISI    348
tr|A0A6N3JBE3|A0A6N3JBE3_FRATN      DVFYLHSRLLERAARVNEEYVEKFTNGEVKGKTGSLTALPIIETQAGDISAFVPTNVISI    348
sp|Q5NQZ1|ATPA_ZYMMO                DVFYLHSRLLERAAKMSDKM-----------GAGSLTALPIIETQAGDVSAYIPTNVISI    345
sp|Q180W8|ATPA_CLOD6                DVFYLHSRLLERAAKLSDEL-----------GGGSMTALPIIETQAGDVSAYIPTNVISI    337
tr|A0A6M4JQ33|A0A6M4JQ33_BACSU      DVFYLHSRLLERAAKLSDAK-----------GAGSITALPFVETQAGDISAYIPTNVISI    337
sp|Q2FF22|ATPA_STAA3                DVFYLHSRLLERAAKLNDDL-----------GGGSITALPIIETQAGDISAYVPTNVISI    337
                                    ************.*::.                    **:*.**::**:*.*:**::*******

tr|L8EUQ4|L8EUQ4_STRR1              TDGQCFLESDLFNAGQRPALNVGISVSRVGGSAQHKAMRQVSGRLRVDLAQFRELEAFAA    409
sp|P0ABB0|ATPA_ECOLI                TDGQIFLETNLFNAGIRPAVNPGISVSRVGGAAQTKIMKKLSGGIRTALAQYRELAAFSQ    408
tr|A0A6N3JBE3|A0A6N3JBE3_FRATN      TDGQIFLETDLFNSGLRPAINPGNSVSRVGGAAQTKIIKKLGGGIRLALAQYRELEAFSQ    408
sp|Q5NQZ1|ATPA_ZYMMO                TDGQIFLETDLFYQGIRPAINVGLSVSRVGGSAAQTKAMKKVAGSIKLELAQYREMAAFAQ    405
sp|Q180W8|ATPA_CLOD6                TDGQIYLQPELFYSGVRPAVDPGISVSRVGGSAQIKAMKKVAGTLKLAYSQYRELAAFSQ    397
tr|A0A6M4JQ33|A0A6M4JQ33_BACSU      TDGQIFLQSDLFFSGVRPAINAGLSVSRVGGSAQIKAMKKVSGTLRLDLASYRELEAFAQ    397
sp|Q2FF22|ATPA_STAA3                TDGQIFLQSDLFFSGVRPAINAGQSVSRVGGSAQIKAMKKVAGTLRLDLASYRELESFAQ    397
                                    **** :*: :**  * ***:: * ***::  * ******.:** * ::::.*  ::   :.:**: :*:

tr|L8EUQ4|L8EUQ4_STRR1              FGSDLDAASKAQLARGQRMTELLKQGQYQPMPVEEQVVSIWAGTTGKMDDVPVQDIRRFE    469
sp|P0ABB0|ATPA_ECOLI                FASDLDDATRKQLDHGQKVTELLKQKQYAPMSVAQQSLVLFAAERGYLADVELSKIGSFE    468
tr|A0A6N3JBE3|A0A6N3JBE3_FRATN      FASDLDEATRAQLNRGQRVTELLKQKQFSTLSVALMALSLYAADNGYLDNLEVSEVIPFE    468
sp|Q5NQZ1|ATPA_ZYMMO                FGSDLDASTQKLLNRGKRLTELLKQPQFHPMPFEEQVVSLFAGTNGYIDGIEVSDVNRYE    465
sp|Q180W8|ATPA_CLOD6                FGSDLDEDTKKRLAQGERIVEILKQGEHQPIKVENQVMIIYAVINNHLEDIPIDNIARFE    457
tr|A0A6M4JQ33|A0A6M4JQ33_BACSU      FGSDLDQATQAKLNRGARTVEVLKQDLNKPLPVEKQVAILYALTKGYLDDIPVADIRRFE    457
sp|Q2FF22|ATPA_STAA3                FGSDLDEFTASKLERGKRTVEVLKQDQNKPLPVEHQVLIIYALTKGYLDDIPVVDITRFE    457
                                    *.****   :     *  : :* :  .*:***       : .        ::*     .  :  .: : .:  :*

tr|L8EUQ4|L8EUQ4_STRR1              RELLDWMGREKKELLTSIAEGAKMSDDTIQALADTVATFKQQFETSDGKLLGEDAPVSTS    529
sp|P0ABB0|ATPA_ECOLI                AALLAYVDRDHAPLMQEINQTGGYNDEIEGKLKGILDSFKATQSW---------------    513
tr|A0A6N3JBE3|A0A6N3JBE3_FRATN      SALHALAETKYSDVIAEINETGKYDADIADKLKIIVEDCKANQAW---------------    513
sp|Q5NQZ1|ATPA_ZYMMO                EAMLSYMRSSHNDLLATIRETGDFSDETKSKLTAALDNFAKIFA----------------    509
sp|Q180W8|ATPA_CLOD6                SELYAFVDNNYPEISRKILG--GEDFT--HDLTDAINEFKEKFVEV-------------    500
tr|A0A6M4JQ33|A0A6M4JQ33_BACSU      EEYYMYLDQNHKDLLDGIAKTGNLPAD--EDFKAAIEGFKRTFAPSN-------------    502
sp|Q2FF22|ATPA_STAA3                DELNHWAESNATELLNEIRETGGLPDA--EKFDTAINEFKKSFSKSE-------------    502
                                               .    :    *              :    :

tr|L8EUQ4|L8EUQ4_STRR1              K 530
sp|P0ABB0|ATPA_ECOLI                - 513
tr|A0A6N3JBE3|A0A6N3JBE3_FRATN      - 513
sp|Q5NQZ1|ATPA_ZYMMO                - 509
sp|Q180W8|ATPA_CLOD6                - 500
tr|A0A6M4JQ33|A0A6M4JQ33_BACSU      - 502
sp|Q2FF22|ATPA_STAA3                - 502
```

Figure S12. Protein sequence alignment of ATP synthase subunit alpha (AtpA) indicates conserved pgK sites. PgK sites that have been identified in *S. rimosus* (STRR1) [45], *E. coli* (ECOLI) [46], *F. novicida* (FRATN) [47], *Z. mobilis* (ZYMMO) [48], *C. difficile* (CLOD6) [44], *B. subtilis* (BACSU) [38,39], and *S. aureus* (STAA3) [39] are highlighted in red.

```
sp|P0A6T5|GCH1_ECOLI        --------------MPSLSKEAALVHEALVARGLETPLRPPVHEMDNETRKSLIAGHMTE 46
tr|Q5NN57|Q5NN57_ZYMMO      ---------------------MEEIQDL-------AVMRPDQVN-VPEE----IQEAIRS 27
sp|Q55759|GCH1_SYNY3        MTIASSHSINNGMNTGKLVNQLSSLPDRNTHNGQEVKYHPENVELNKEK----MMDAVRV 56
sp|Q04MF9|GCH1_STRP2        -------------------------------------------MDTQK----IEAAVKM 12
sp|B5XLE8|GCH1_STRPZ        -----------------------------------MKKERLMSINKEK----AEAAIYQ 20
                                                                   :         :  

sp|P0A6T5|GCH1_ECOLI        IMQLLNLDLADDSLMETPHRIAKMYVDEIFSGLDYANFPK--ITLIENKMKVDEMVTVRD 104
tr|Q5NN57|Q5NN57_ZYMMO      LIRWAGDDPDREGLIDTPKRVAKAWK-YYCRGYREDPENH-LKTVFEEVGGYDDIVLLKD 85
sp|Q55759|GCH1_SYNY3        MLESVGEDPEREGLLKTPKRVAEAMQ-FLTQGYDQSLEKLVNGAIFDE--GHNEMVLVRD 113
sp|Q04MF9|GCH1_STRP2        IIEAVGEDANREGLQETPARVARMYQ-EIFSGLGQTAEEH-LSKSFEI--IDDNMVVEKD 68
sp|B5XLE8|GCH1_STRPZ        FLEAIGENPNREGLLDTPKRVAKMYA-EMFLGLGKDPKEE-FTAVFKE--QHEDVVIVKG 76
                            ::.  . :   :.* .** *:*.        *             :.     :::*  :.

sp|P0A6T5|GCH1_ECOLI        ITLTSTCEHHFVTIDGKATVAYIP-KDSVIGLSKINRIVQFFAQRPQVQERLTQQILIAL 163
tr|Q5NN57|Q5NN57_ZYMMO      IPFYSHCEHHMAPIMGHAHIAYLP-DKKVVGLSKLARVLKGYAHRLQIQERLTAQVADCI 144
sp|Q55759|GCH1_SYNY3        IDFFSLCEHHMLPFMGKAHLAYIP-NQKVVGLSKLARIVEMFSRRLQVQERLTRQIAEAV 172
sp|Q04MF9|GCH1_STRP2        IFFHTMCEHHFLPFYGRAHIAYIP-DGRVAGLSKLARTVEVYSKKPQIQERLNIEVADAL 127
sp|B5XLE8|GCH1_STRPZ        ISFYSICEHHLVPFYGKAHIAYLPSDGRVTGLSKLARAVEVASKRPQLQERLTSQIADAL 136
                            * : : ****:  : *:* :**:* .  * ****: * ::  ::: *:****. ::  .:

sp|P0A6T5|GCH1_ECOLI        QTLLGTNNVAVSIDAVHYCVKARGIRDATSATTTTSLGGLFKSSQNTRHEFLRAVRHHN- 222
tr|Q5NN57|Q5NN57_ZYMMO      WNNLRPRGVAVVISAVHSCMAARGVETPNVQTVTKTLRGIYSEDPEQAKMILNLMGV--- 201
sp|Q55759|GCH1_SYNY3        QEILDPQGVAVVMEATHMCMVMRGVQKPGSWTVTSAMIGSFQNEQKTREEFLNLIRHQPN 232
sp|Q04MF9|GCH1_STRP2        MDYLGAKGAFVIIEAEHMCMSMRGVRKPGTATLTTVARGLFETDKDLRDQAYRLMGL--- 184
sp|B5XLE8|GCH1_STRPZ        VEALNPKGTLVMVEAEHMCMTMRGIKKPGSKTITTTARGLYKESRAERQEVISLMTKD-- 194
                               *  ... * :.* * *:  **:.     * *.   * :. .    .     :

sp|P0A6T5|GCH1_ECOLI        --        222
tr|Q5NN57|Q5NN57_ZYMMO      --        201
sp|Q55759|GCH1_SYNY3        FY        234
sp|Q04MF9|GCH1_STRP2        --        184
sp|B5XLE8|GCH1_STRPZ        --        194
```

Figure S13. Protein sequence alignment of GTP cyclohydrolase 1 (Gch1) indicates conserved pgK sites. PgK sites that have been identified in *E. coli* (ECOLI) [46], *Z. mobilis* (ZYMMO) [48], *Synechocystis* sp. (SYNY3) [40,41], *S. pneumoniae* (STRP2) [37], and *S. pyogenes* M49 (STRPZ) [this work] are highlighted in red.

```
tr|L8ETF9|L8ETF9_STRR1              MAKLTQDELLAQFEEMTLIELSDFVKKFEEKFDVEAAAPAAVAVAAPGQGGGEAAAAEEQ      60
sp|P0A7K2|RL7_ECOLI                 -MSITKDQIIEAVAAMSVMDVVELISAMEEKFGVSAAAAVAVAAGP-------VEAAEEK      52
tr|A0A6N3JBG2|A0A6N3JBG2_FRATN      -MAITKEDILNAVAEMSVMDVCDLVKMMEDKFGVSAAAAVAVAAGPV---AGPAEAAEEK      56
sp|Q5NPK7|RL7_ZYMMO                 --MADLAKIVEDLSALTVLEAAELSKLLEEKWGVSASAAVAVAAAPA---AGG-AAAEEK      54
sp|Q18CE7|RL7_CLOD6                 ---MTIEQILEAIENMKVLELNELVKAAEEKFGVSASAPVMVAGAA----AGG-PAAEEK      52
sp|Q2FJA0|RL7_STAA3                 --MANHEQIIEAIKEMSVLELNDLVKAIEEEFGVTAAAPVAVAGAA----GGA-DAAAEK      53
sp|P66063|RL7_STRP1                 -MALNIENIIAEIKEASILELNDLVKAIEEEFGVTAAAPVAAAA------AGG-AEEAAK      52
RefSeq|WP_002984819.1|S. pyogenes   -MALNIENIIAEIKEASILELNDLVKAIEEEFGVTAAAPVAAAA-----AGGA--EEAAK      52
tr|A0A6M3Z765|A0A6M3Z765_BACSU      -MALNIEEIIASVKEATVLELNDLVKAIEEEFGVTAAAPVAVAGGA----AAG-GAAEEQ      54
sp|Q8YAA3|RL7_LISMO                 -MALNIEEIIASVKEASVLELNDLVKAIEEEFGVTAAAPVAVA-------AAG-GAAAEQ      51

tr|L8ETF9|L8ETF9_STRR1              DEFDVILTGAGDKKIQVIKVVRELTSLGLKEAKDLVDGTPKPVLEKVAKDAADKAAEALK      120
sp|P0A7K2|RL7_ECOLI                 TEFDVILKAAGANKVAVIKAVRGATGLGLKEAKDLVESAPAALKEGVSKDDAEALKKALE      112
tr|A0A6N3JBG2|A0A6N3JBG2_FRATN      TEFDVVLVDAGSNKIAAIKAVRGATGLGLKEAKDAVEGTPFTVKEAASKEEAEALKKQLE      116
sp|Q5NPK7|RL7_ZYMMO                 TEFDVILTGDGGKKINVIKEIRAITGLGLTDAKALVEGAPKPVKEGVAKDEAEKLKKQLE      114
sp|Q18CE7|RL7_CLOD6                 TEFDVVLTDVGSSKVGVIKAVREITGLGLKEAKEVVDNAPKTVKEGASKEEADQIKEKLE      112
sp|Q2FJA0|RL7_STAA3                 TEFDVELTSAGSSKIKVVKAVKEATGLGLKDAKELVDGAPKVIKEALPKEEAEKLKEQLE      113
sp|P66063|RL7_STRP1                 DSFDVELTSAGDKKVGVIKAVREITGLGLKEAKGLVDGAPANVKEGVAAAEAEEIKAKLE      112
RefSeq|WP_002984819.1|S. pyogenes   DSFDVELTSAGDKKVGVIKAVREITGLGLKEAKGLVDGAPANVKEGVAAAEAEEIKAKLE      112
tr|A0A6M3Z765|A0A6M3Z765_BACSU      SEFDLILAGAGSQKIKVIKVVREITGLGLKEAKELVDNTPKPLKEGIAKEEAEELKAKLE      114
sp|Q8YAA3|RL7_LISMO                 TEFTVELASAGDSKIKVIKVVREITGLGLKEAKELVDNAPKALKEGIAKDEAEEIKAKLE      111
                                          :   * .*: .:* ::  *.***.:**  *:.:*  : *      *:     *:

tr|L8ETF9|L8ETF9_STRR1              GAGASVEVK      129
sp|P0A7K2|RL7_ECOLI                 EAGAEVEVK      121
tr|A0A6N3JBG2|A0A6N3JBG2_FRATN      EAGAKVELK      125
sp|Q5NPK7|RL7_ZYMMO                 AAGATVELK      123
sp|Q18CE7|RL7_CLOD6                 AAGAKVEVK      121
sp|Q2FJA0|RL7_STAA3                 EVGATVELK      122
sp|P66063|RL7_STRP1                 EAGATITLK      121
RefSeq|WP_002984819.1|S. pyogenes   EAGATITLK      121
tr|A0A6M3Z765|A0A6M3Z765_BACSU      EVGASVEVK      123
sp|Q8YAA3|RL7_LISMO                 EVGANVEVK      120
                                     .** : :*
```

Figure S14. Protein sequence alignment of large ribosomal subunit protein bL12 (RplL) indicates conserved pgK sites. PgK sites that have been identified in *S. rimosus* (STRR1) [45], *E. coli* (ECOLI) [46], *F. novicida* (FRATN) [47], *Z. mobilis* (ZYMMO) [48], *C. difficile* (CLOD6) [44], *S. aureus* (STAA3) [39], *S. pyogenes* M1 (STRP1) [35], *S. pyogenes* M49 strain 591 [this work], *B. subtilis* (BACSU) [38], and *L. monocytogenes* (LISMO) [35] and are highlighted in red. The proteome sequence of *S. pyogenes* serotype M49, strain NZ131 which was used typically for peptide identification is significantly shortened at the N-terminus of RplL. Therefore, the new proteome sequence of *S. pyogenes* serotype M49, strain 591 (UniParc UP000814797 derived from NCBI RefSeq assembly GCF_000167435.2) was used.

```
tr|L8ETG4|L8ETG4_STRR1              -----------------MAKAKFERTKPHVNIGTIGHIDHGKTTLTAAITKVLHDAYPD-      42
sp|Q5NQ65|EFTU_ZYMMO                -----------------MAKAKFERNKPHCNIGTIGHVDHGKTTLTAAITKVLAEAGGG-      42
tr|A0A6N3S1E1|A0A6N3S1E1_BORPT      ---------------------------------------------------MLSNKFGG-      8
sp|P0CE47|EFTU1_ECOLI               -----------------MSKEKFERTKPHVNVGTIGHVDHGKTTLTAAITTVLAKTYGG-      42
tr|A0A6N3JC70|A0A6N3JC70_FRATN      -----------------MAKEKFERSKPHVNVGTIGHVDHGKTTLTAAITKVMAEKNGG-      42
sp|Q18CE4|EFTU_CLOD6                -----------------MAKAKYERTKPHVNIGTIGHVDHGKTTLTAAITKTLYDR--YQ      41
tr|J7M780|J7M780_STRP1              MAYRLSIKYILLEAFPKMAKEKYDRSKPHVNIGTIGHVDHGKTTLTAAITTVLARRLPSS      60
sp|B5XKI1|EFTU_STRPZ                -----------------MAKEKYDRSKPHVNIGTIGHVDHGKTTLTAAITTVLARRLPTS      43
sp|Q839G8|EFTU_ENTFA                -----------------MAKEKFDRSKSHVNIGTIGHVDHGKTTLTAAIATVLSKH-GG-      41
sp|Q2FJ92|EFTU_STAA3                -----------------MAKEKFDRSKEHANIGTIGHVDHGKTTLTAAIATVLAKN-GD-      41
sp|Q8Y422|EFTU_LISMO                -----------------MAKEKFDRSKPHVNIGTIGHVDHGKTTLTAAITTVLAKK-GY-      41
tr|A0A6M3Z6X2|A0A6M3Z6X2_BACSU      -----------------MAKEKFDRSKSHANIGTIGHVDHGKTTLTAAITTVLHKKSGK-      42
                                                                                    :

tr|L8ETG4|L8ETG4_STRR1              LNEASAFDQIDKAPEERQRGITISIAHVEYQTENRHYAHVDCPGHADYIKNMITGAAQMD      102
sp|Q5NQ65|EFTU_ZYMMO                -NTFVDYANIDKAPEERERGITISTSHVEYETETRHYAHVDCPGHADYVKNMITGAAQMD      101
tr|A0A6N3S1E1|A0A6N3S1E1_BORPT      -E-ARGYDQIDAAPEEKARGITINTSHVEYETETRHYAHVDCPGHADYVKNMITGAAQMD      66
sp|P0CE47|EFTU1_ECOLI               -A-ARAFDQIDNAPEEKARGITINTSHVEYDTPTRHYAHVDCPGHADYVKNMITGAAQMD      100
tr|A0A6N3JC70|A0A6N3JC70_FRATN      -M-ARKFDEIDSAPEEKARGITINTSHVEYESPNRHYAHVDCPGHADYVKNMITGAAQMD      100
sp|Q18CE4|EFTU_CLOD6                LGEAVDFANIDKAPEERERGITISTAHVEYETPNRHYAHVDCPGHADYVKNMITGAAQMD      101
tr|J7M780|J7M780_STRP1              VNQPKDYASIDAAPEERERGITINTAHVEYETATRHYAHIDAPGHADYVKNMITGAAQMD      120
sp|B5XKI1|EFTU_STRPZ                VNQPKDYASIDAAPEERERGITINTAHVEYETETRHYAHIDAPGHADYVKNMITGAAQMD      103
sp|Q839G8|EFTU_ENTFA                -GEAQSYDSIDNAPEEKERGITINTSHIEYETETRHYAHVDCPGHADYVKNMITGAAQMD      100
sp|Q2FJ92|EFTU_STAA3                -SVAQSYDMIDNAPEEKERGITINTSHIEYQTDKRHYAHVDCPGHADYVKNMITGAAQMD      100
sp|Q8Y422|EFTU_LISMO                -ADAQAYDQIDGAPEERERGITISTAHVEYQTDSRHYAHVDCPGHADYVKNMITGAAQMD      100
tr|A0A6M3Z6X2|A0A6M3Z6X2_BACSU      -GTAMAYDQIDGAPEERERGITISTAHVEYETETRHYAHVDCPGHADYVKNMITGAAQMD      101
                                          :  ** ****: *****. :*:**:: .*****:*.******:***********

tr|L8ETG4|L8ETG4_STRR1              GAILVVAATDGPMPQTKEHVLLARQVGVPYIVVALNKADMVDDEEILELVELEVRELLSE      162
sp|Q5NQ65|EFTU_ZYMMO                GAILVVSAADGPMPQTREHILLARQVGVPALVVFMNKVDQVDDPELLELVEMEIRELLSS      161
tr|A0A6N3S1E1|A0A6N3S1E1_BORPT      GAILVVSAADGPMPQTREHILLSRQVGVPYIIVFLNKADMVDDAELLELVEMEVRELLSK      126
sp|P0CE47|EFTU1_ECOLI               GAILVVAATDGPMPQTREHILLGRQVGVPYIIVFLNKCDMVDDEELLELVEMEVRELLSQ      160
tr|A0A6N3JC70|A0A6N3JC70_FRATN      GAILVCSAADGPMPQTREHILLSRQVGVPKIVVFLNKCDMVDDEELLELVEMEVRELLDQ      160
sp|Q18CE4|EFTU_CLOD6                GAILVCSATDGPMPQTREHILLSRQVGVPYIVVFLNKCDMVDDEELLELVEMEVRDLLTE      161
tr|J7M780|J7M780_STRP1              GAILVVASTDGPMPQTREHILLSRQVGVKHLIVFMNKVDLVDDEELLELVEMEIRDLLSE      180
sp|B5XKI1|EFTU_STRPZ                GAILVVASTDGPMPQTREHILLSRQVGVKHLIVFMNKVDLVDDEELLELVEMEIRDLLSE      163
sp|Q839G8|EFTU_ENTFA                GAILVVSAADGPMPQTREHILLSRNVGVPYIVVFLNKMDMVDDEELLELVEMEVRDLLSE      160
sp|Q2FJ92|EFTU_STAA3                GGILVVSAADGPMPQTREHILLSRNVGVPALVVFLNKVDMVDDEELLELVEMEVRDLLSE      160
sp|Q8Y422|EFTU_LISMO                GAILVVSAADGPMPQTREHILLSRQVGVPYIVVFMNKCDMVDDEELLELVEMEIRDLLTE      160
tr|A0A6M3Z6X2|A0A6M3Z6X2_BACSU      GAILVVSAADGPMPQTREHILLSKNVGVPYIVVFLNKCDMVDDEELLELVEMEVRDLLSE      161
                                    *.*** :::*******:**:**.::***  ::* :** * *** *:*****:*:*:** .

tr|L8ETG4|L8ETG4_STRR1              YEFPGDDVPVVKVSALKALEG-DKEW--GDSVLKLMAAVDESIPQPERDVDKPFLMPIED      219
sp|Q5NQ65|EFTU_ZYMMO                YDFPGDDIPIVKGSALAALED-KNPEIGKEAILSLMAAVDEYIPQPERPLDKSFLMPIED      220
tr|A0A6N3S1E1|A0A6N3S1E1_BORPT      YDFPGDDTPIVKGSAKLALEG-DKGELGEQAILSLAQALDTYIPTPERAVDGAFLMPVED      185
sp|P0CE47|EFTU1_ECOLI               YDFPGDDTPIVRGSALKALEG-DAEW--EAKILELAGFLDSYIPEPERAIDKPFLLPIED      217
tr|A0A6N3JC70|A0A6N3JC70_FRATN      YEFPGDDTPVIMGSALRAIEG-DEAY--VEKIVELVQAMDDYIPAPERDTEKPFILPIED      217
sp|Q18CE4|EFTU_CLOD6                YDFPGDDTPIVRGSALMALEDPKSEW--GDKIVELFEQIDEYIPAPERDTDKPFLMPVED      219
tr|J7M780|J7M780_STRP1              YDFPGDDLPVIQGSALKALEG-DTKF--EDIIMELMDTVDSYIPEPERDTDKPLLLPVED      237
sp|B5XKI1|EFTU_STRPZ                YDFPGDDLPVIQGSALKALEG-DSKY--EDIIMELMSTVDEYIPEPERDTDKPLLLPVED      220
sp|Q839G8|EFTU_ENTFA                YDFPGDDVPVIAGSALKALEG-DESY--EEKILELMAAVDEYIPTPERDTDKPFMMPVED      217
sp|Q2FJ92|EFTU_STAA3                YDFPGDDVPVIAGSALKALEG-DAQY--EEKILELMEAVDTYIPTPERDSDKPFMMPVED      217
sp|Q8Y422|EFTU_LISMO                YEFPGDDIPVIKGSALKALQG-EADW--EAKIDELMEAVDSYIPTPERDTDKPFMMPVED      217
tr|A0A6M3Z6X2|A0A6M3Z6X2_BACSU      YDFPGDDVPVVKGSALKALEG-DAEW--EAKIFELMDAVDEYIPTPERDTEKPFMMPVED      218
                                    *:***** *::  **   *::. .        : .*   :*  ** ***  :  :::*:**

tr|L8ETG4|L8ETG4_STRR1              VFTITGRGTVVTGRIERGVLKVNETVDIIGIKTEKTTTTVTGIEMFRKLLDEGQAGENVG      279
sp|Q5NQ65|EFTU_ZYMMO                VFSISGRGTVVTGRVETGIVKVGEEVEIVGLRD-TKKTTVTGVEMFRKLLDQGQAGDNIG      279
tr|A0A6N3S1E1|A0A6N3S1E1_BORPT      VFSISGRGTVVTGRIERGVVKVGEEIEIVGIKP-TVKTTCTGVEMFRKLLDQGQAGDNVG      244
sp|P0CE47|EFTU1_ECOLI               VFSISGRGTVVTGRVERGIIKVGEEVEIVGIKE-TQKSTCTGVEMFRKLLDEGRAGENVG      276
tr|A0A6N3JC70|A0A6N3JC70_FRATN      VFSISGRGTVVTGRIERGVVNVGDEVEVVGIRP-TQKTTVTGVEMFRKLLDRGEAGDNVG      276
sp|Q18CE4|EFTU_CLOD6                VFSITGRGTVATGRVERGVLKVQDEVELVGLTEAPRKVVVTGVEMFRKLLDQAQAGDNIG      279
tr|J7M780|J7M780_STRP1              VFSITGRGTVASGRIDRGTVRVNDEIEIVGIKEETKKAVVTGVEMFRKQLDEGLAGDNVG      297
sp|B5XKI1|EFTU_STRPZ                VFSITGRGTVASGRIDRGTVRVNDEIEIVGIKEETKKAVVTGVEMFRKQLDEGLAGDNVG      280
sp|Q839G8|EFTU_ENTFA                VFSITGRGTVATGRVERGEVRVGDEVEIVGIKDETSKTTVTGVEMFRKLLDYAEAGDNIG      277
sp|Q2FJ92|EFTU_STAA3                VFSITGRGTVATGRVERGQIKVGEEVEIIGLHD-TSKTTVTGVEMFRKLLDYAEAGDNIG      276
sp|Q8Y422|EFTU_LISMO                VFSITGRGTVATGRVERGQVKVGDEVEVIGIEEESKKVVVTGVEMFRKLLDYAEAGDNIG      277
tr|A0A6M3Z6X2|A0A6M3Z6X2_BACSU      VFSITGRGTVATGRVERGQVKVGDEVEIIGLQEENKKTTVTGVEMFRKLLDYAEAGDNIG      278
                                    **:*:*****.:**:: * :.* : ::::*:     . . **:***** ** . **:*:*

tr|L8ETG4|L8ETG4_STRR1              LLLRGIKREDVERGQVIIKPGSVTPHTEFEAQAYILSKDEGGRHTPFFNNYRPQFYFRTT      339
sp|Q5NQ65|EFTU_ZYMMO                ALLRGTARTEVERGQVLAKPGSITPHTEFKAEVYVLSKDEGGRHTPFFANYRPQFYFRTT      339
tr|A0A6N3S1E1|A0A6N3S1E1_BORPT      ILLRGTKREDVERGQVLAKPGSINPHTDFTAEVYILSKEEGGRHTPFFNGYRPQFYFRTT      304
sp|P0CE47|EFTU1_ECOLI               VLLRGIKREEIERGQVLAKPGTIKPHTKFESEVYILSKDEGGRHTPFFKGYRPQFYFRTT      336
tr|A0A6N3JC70|A0A6N3JC70_FRATN      ILVRGLKRDDVERGQVLCKPGSIKPHTKFEAEVYVLSKEEGGRHTPFFKGYRPQFYFRTT      336
sp|Q18CE4|EFTU_CLOD6                ALLRGVQRNEIERGQVLAKTGSVKAHTKFTAEVYVLKKEEGGRHTPFFDGYRPQFYFRTT      339
tr|J7M780|J7M780_STRP1              ILLRGVQRDEIERGQVIAKPSSINPHTKFKGEVYILSKDEGGRHTPFFNNYRPQFYFRTT      357
sp|B5XKI1|EFTU_STRPZ                ILLRGVQRDEIERGQVIAKPGSINPHTKFKGEVYILSKDEGGRHTPFFNNYRPQFYFRTT      340
sp|Q839G8|EFTU_ENTFA                ALLRGVAREDIERGQVLAKPATITPHTKFKAEVYVLSKEEGGRHTPFFTNYRPQFYFRTT      337
sp|Q2FJ92|EFTU_STAA3                ALLRGVAREDVQRGQVLAAPGSITPHTEFKAEVYVLSKDEGGRHTPFFSNYRPQFYFRTT      336
sp|Q8Y422|EFTU_LISMO                ALLRGVAREDIQRGQVLAKPGSITPHTNFKAETYVLTKEEGGRHTPFFNNYRPQFYFRTT      337
tr|A0A6M3Z6X2|A0A6M3Z6X2_BACSU      ALLRGVSREEIQRGQVLAKPGTITPHSKFKAEVYVLSKEEGGRHTPFFSNYRPQFYFRTT      338
                                     *:**  * :::****:   .::. *:.*  .:.*:*.*:********* .*********

tr|L8ETG4|L8ETG4_STRR1              DVTGVVTLPEGTEMVMPGDNTEMSVQLIQPVAMEEGLKFAIREGGRTVGAGQVTKIVK        397
sp|Q5NQ65|EFTU_ZYMMO                DVTGEITLPEDVEMVMPGDNIAFGVKLIAPIAMDPGLRFAIREGGRTVGAGVVSSIIK        397
tr|A0A6N3S1E1|A0A6N3S1E1_BORPT      DVTGTIDLPADKEMVLPGDNVSMTVKLLAPIAMEEGLRFAIREGGRTVGAGVVAKIIK        362
sp|P0CE47|EFTU1_ECOLI               DVTGTIELPEGVEMVMPGDNIKMVVTLIHPIAMDDGLRFAIREGGRTVGAGVVAKVLG        394
tr|A0A6N3JC70|A0A6N3JC70_FRATN      DITGAVELPEGVEMVMPGDNVKMTITLINPIAMDEGLRFAIREGGRTVGAGVVAKIIE        394
sp|Q18CE4|EFTU_CLOD6                DVTGACKLPEGIEMVMPGDNVTMEVDLINSIVVEEGLRFSIREGGRTVASGVVATIIE        397
tr|J7M780|J7M780_STRP1              DVTGSIELPAGTEMVMPGDNVTINVELIHPIAVEQGTTFSIREGGRTVGSGIVSEIEA        415
sp|B5XKI1|EFTU_STRPZ                DVTGSIELPAGTEMVMPGDNVTINVELIHPIAVEQGTTFSIREGGRTVGSGIVSEIEA        398
sp|Q839G8|EFTU_ENTFA                DVTGVVELPEGTEMVMPGDNVAMDVELIHPIAIEDGTRFSIREGGRTVGSGVVTEIVK        395
sp|Q2FJ92|EFTU_STAA3                DVTGVVHLPEGTEMVMPGDNVEMTVELIAPIAIEDGTRFSIREGGRTVGSGVVTEIIK        394
sp|Q8Y422|EFTU_LISMO                DVTGIVTLPEGTEMVMPGDNIELAVELIAPIAIEDGTKFSIREGGRTVGAGVVSNISK        395
tr|A0A6M3Z6X2|A0A6M3Z6X2_BACSU      DVTGIIHLPEGVEMVMPGDNTEMNVELISTIAIEEGTRFSIREGGRTVGSGVVSTITE        396
                                    *:**   ** . ***:****  : : *:  :.:: *  *:********.:* *: :
```

Figure S15. Protein sequence alignment of elongation factor Tu (EF-Tu) indicates conserved pgK sites. PgK sites that have been identified in *S. rimosus* (STRR1) [45], *Z. mobilis* (ZYMMO) [48], *B. pertussis* (BORPT) [49], *E. coli* (ECOLI) [46], *F. novicida* (FRATN) [47], *C. difficile* (CLOD6) [44], *S. pyogenes* M1 (STRP1) [35], *S. pyogenes* M49

(STRPZ) [this work], *E. faecalis* (ENTFA) [43], *S. aureus* (STAA3) [39], *L. monocytogenes* (LISMO) [35], and *B. subtilis* (BACSU) [38,39,35] and are highlighted in red.

```
sp|P48220|CH60_ZYMMO                 MAAKDVKFSRDARERILRGVDILADAVKVTLGPKGRNVVLDKAFGAPRITKDGVSVAKEI     60
tr|A0A0E8E5E7|A0A0E8E5E7_BORPT       MAAKQVLFADEARVRIVRGVNVLANAVKTTLGPKGRNVVLERSFGAPTVTKDGVSVAKEI     60
sp|P0A6F5|CH60_ECOLI                 MAAKDVKFGNDARVKMLRGVNVLADAVKVTLGPKGRNVVLDKSFGAPTITKDGVSVAREI     60
tr|A0A6N3JC79|A0A6N3JC79_FRATN       MAAKQVLFSDEARAKMLDGVNTLANAVKVTLGPKGRNVVLDKSFGAPTITKDGVSVAKEI     60
tr|L8EHR8|L8EHR8_STRR1               -MAKIIAFDEEARRGLERGMNQLADAVKVTLGPKGRNVVLEKKWGAPTITNDGVSIAKEI     59
sp|Q18CT5|CH60_CLOD6                 -MAKEIKFSEETRRALEAGVNKLADTVKVTLGPKGRNVILDKKFGSPLITNDGVTIAKEI     59
sp|B5XIW7|CH60_STRPZ                 -MAKDIKFSADARAAMVRGVDMLADTVKVTLGPKGRNVVLEKAFGSPLITNDGVTIAKEI     59
sp|Q2FF95|CH60_STAA3                 -MVKQLKFSEDARQAMLRGVDQLANAVKVTIGPKGRNVVLDKEFTAPLITNDGVTIAKEI     59
tr|A0A6M3Z7Y6|A0A6M3Z7Y6_BACSU       -MAKEIKFSEEARRAMLRGVDALADAVKVTLGPKGRNVVLEKKFGSPLITNDGVTIAKEI     59
sp|Q9AGE6|CH60_LISMO                 -MAKDIKFSEDARRAMLRGVDQLANAVKVTLGPKGRNVVLEKKFGSPLITNDGVTIAKEI     59
                                       .* : *  ::*  :  *:: **::**.*:*******:*:: : :* :*:***::*:**

sp|P48220|CH60_ZYMMO                 ELKDKFENMGAQMLREVASKTNDLAGDGTTTATVLAQAIVREGMKSVAAGMNPMDLKRGI     120
tr|A0A0E8E5E7|A0A0E8E5E7_BORPT       ELKDKFENIGAQLVKDVASKTSDNAGDGTTTATVLAQAVVQEGLKYVAAGFNPIDLKRGI     120
sp|P0A6F5|CH60_ECOLI                 ELEDKFENMGAQMVKEVASKANDAAGDGTTTATVLAQAIITEGLKAVAAGMNPMDLKRGI     120
tr|A0A6N3JC79|A0A6N3JC79_FRATN       ELEDKFENMGAQIVKEVASKTADVAGDGTTTATVLAQALLTEGLKAVAAGMNPMDLKRGI     120
tr|L8EHR8|L8EHR8_STRR1               ELEDPYEKIGAELVKEVAKKTDDVAGDGTTTATVLAQALVREGLRNVAAGANPMALKRGI     119
sp|Q18CT5|CH60_CLOD6                 ELEDRFENMGAQLVKEVATKTNDVAGDGTTTATVLAQAIIREGLKNVTAGANPILLRKGI     119
sp|B5XIW7|CH60_STRPZ                 ELEDHFENMGAKLVSEVASKTNDIAGDGTTTATVLTQAIVHEGLKNVTAGANPIGIRRGI     119
sp|Q2FF95|CH60_STAA3                 ELEDPYENMGAKLVQEVANKTNEIAGDGTTTATVLAQAMIQEGLKNVTSGANPVGLRQGI     119
tr|A0A6M3Z7Y6|A0A6M3Z7Y6_BACSU       ELEDAFENMGAKLVAEVASKTNDVAGDGTTTATVLAQAMIREGLKNVTAGANPVGVRKGM     119
sp|Q9AGE6|CH60_LISMO                 ELEDPFENMGAKLVSEVASKTNDVAGDGTTTATVLAQAMIQEGLKNVTAGANPVGVRRGI     119
                                     **:* :*::**::: :**.*: : ***********:**:: **:: *::* **: :::*:

sp|P48220|CH60_ZYMMO                 DLAATKVVESLRSRSKPVSDFNEVAQVGIISANGDEEVGRRIAEAMEKVGKEGVITVEEA     180
tr|A0A0E8E5E7|A0A0E8E5E7_BORPT       DKAVAAAVEELKKLSKPVTTSKEIAQVGSISANSDASIGQIIADAMDKVGKEGVITVEDG     180
sp|P0A6F5|CH60_ECOLI                 DKAVTAAVEELKALSVPCSDSKAIAQVGTISANSDETVGKLIAEAMDKVGKEGVITVEDG     180
tr|A0A6N3JC79|A0A6N3JC79_FRATN       DKATAKLVEELKALSKPCSDPKSIEQVGTISANSDATVGKLIADAMAKVGKEGVITVEEG     180
tr|L8EHR8|L8EHR8_STRR1               ERAVEAVSAALLEQAKDVETKEQIASTASISAA-DTQIGELIAEAMDKVGKEGVITVEES     178
sp|Q18CT5|CH60_CLOD6                 QKAVTVAVEELKNQSRIVETQEAISQVASISAG-DEEVGKLIAEAMEIVGKDGVITVEES     178
sp|B5XIW7|CH60_STRPZ                 ETATATAVEALKAIAQPVSGKEAIAQVAAVSSR-SEKVGEYISEAMERVGNDGVITIEES     178
sp|Q2FF95|CH60_STAA3                 DKAVKVAVEALHENSQKVENKNEIAQVGAISAA-DEEIGRYISEAMEKVGNDGVITIEES     178
tr|A0A6M3Z7Y6|A0A6M3Z7Y6_BACSU       EQAVAVAIENLKEISKPIEGKESIAQVAAISAA-DEEVGSLIAEAMERVGNDGVITIEES     178
sp|Q9AGE6|CH60_LISMO                 EKAVATAIEELKAISKPIESKESIAQVAAISSG-DEEVGKLIAEAMERVGNDGVITIEES     178
                                     : *.       *   :       : : ... :*:  .  :*  *::**  **::****:*:.

sp|P48220|CH60_ZYMMO                 KGFDFELDVVEGMQFDRGYLSPYFITNPEKMVAELADPYILIYEKKLSNLQSILPILESV     240
tr|A0A0E8E5E7|A0A0E8E5E7_BORPT       KSLENELDVVEGMQFDRGYLSPYFINSPEKQVAALDDPYVLIYDKKVSNIRDLLPVLEQV     240
sp|P0A6F5|CH60_ECOLI                 TGLQDELDVVEGMQFDRGYLSPYFINKPETGAVELESPFILLADKKISNIREMLPVLEAV     240
tr|A0A6N3JC79|A0A6N3JC79_FRATN       KGFEDELDVVEGMQFDRGYLSPYFATNQENMTTDLENPYILIVDKKISNIRDLLPVLEGV     240
tr|L8EHR8|L8EHR8_STRR1               QTFGLELELTEGMRFDKGYISAYFATDMERMEASLDDPYILIVNSKISNVKDLLPLLEKV     238
sp|Q18CT5|CH60_CLOD6                 QTMNTELDAVEGMQFDRGFVSAYMVTDVDKMEAVLNDPYILITDKKISNIQELLPVLEQI     238
sp|B5XIW7|CH60_STRPZ                 RGMETELEVVEGMQFDRGYLSQYMVTDNEKMVADLENPFILITDKKVSNIQDILPLLEEV     238
sp|Q2FF95|CH60_STAA3                 NGLNTELEVVEGMQFDRGYQSPYMVTDSDKMVAELERPYILVTDKKISSFQDILPLLEQV     238
tr|A0A6M3Z7Y6|A0A6M3Z7Y6_BACSU       KGFTTELEVVEGMQFDRGYASPYMVTDSDKMEAVLDNPYILITDKKITNIQEILPVLEQV     238
sp|Q9AGE6|CH60_LISMO                 KGFATELDVVEGMQFDRGYTSPYMVTDSDKMEAVLEKPYILITDKKINNIQEILPVLEQV     238
                                       :  **: .***:**:*: * *: .. :    . *  *::*: :.*:...:.:**:** :

sp|P48220|CH60_ZYMMO                 VQSGRPLLIIAEDIEGEALATLVVNKLRGGLKVAAVKAPGFGDRRKAMLEDIAILTKGEL     300
tr|A0A0E8E5E7|A0A0E8E5E7_BORPT       AKSSRPLLIIAEDVEGEALATLVVNNIRGILKTTAVKAPGFGDRRKAMLEDIAILTGGTV     300
sp|P0A6F5|CH60_ECOLI                 AKAGKPLLIIAEDVEGEALATLVVNTMRGIVKVAAVKAPGFGDRRKAMLQDIATLTGGTV     300
tr|A0A6N3JC79|A0A6N3JC79_FRATN       SKSGRALLIIAEDVESEALATLVVNNMRGVVKVCAVKAPGFGDRRKAMLEDIATLTGATF     300
tr|L8EHR8|L8EHR8_STRR1               MQSGKPLLIIAEDVEGEALSTLVVNKIRGTFKSVAVKAPGFGDRRKAMLGDIAILTGGTV     298
sp|Q18CT5|CH60_CLOD6                 VQQGKKLLIIAEDVEGEALSTLVVNKLRGTFDVVAVKAPGFGDRRKEMLQDIAILTGAQV     298
sp|B5XIW7|CH60_STRPZ                 LKTNRPLLIIADDVDGEALPTLVLNKIRGTFSVVAVKAPGFGDRRKAMLEDIAILTGGTV     298
sp|Q2FF95|CH60_STAA3                 VQSNRPILIVADEVEGDALTNIVLNRMRGTFTAVAVKAPGFGDRRKAMLEDLAILTGAQV     298
tr|A0A6M3Z7Y6|A0A6M3Z7Y6_BACSU       VQQGKPLLLIAEDVEGEALATLVVNKLRGTFNAVAVKAPGFGDRRKAMLEDIAVLTGGEV     298
sp|Q9AGE6|CH60_LISMO                 VQQGRPMLIIAEDVEGEAQATLVLNKLRGTFNVVAVKAPGFGDRRKAMLEDIAILTGGQV     298
                                      : .: :*::*::::.:*   .:*:* :** .    ************ ** *:* ** . .

sp|P48220|CH60_ZYMMO                 ISEDLGIKLENVTLNMLGSAKRVSITKENTTIVDGAGDQSTIKDRVEAIRSQIEATTSDY     360
tr|A0A0E8E5E7|A0A0E8E5E7_BORPT       ISEETGMSLEKATLQDLGQAKRIEVAKENTTIIDGAGDGKSIEARVKQIRAQIEEATSDY     360
sp|P0A6F5|CH60_ECOLI                 ISEEIGMELEKATLEDLGQAKRVVINKDTTTIIDGVGEEAAIQGRVAQIRQQIEEATSDY     360
tr|A0A6N3JC79|A0A6N3JC79_FRATN       VSEDLSMKLEETNMEHLGTASRVQVTKDNTTIIDGAGEKEAIAKRINVIKANIAEANSDY     360
tr|L8EHR8|L8EHR8_STRR1               ISEEVGLKLENAGLDLLGRARKVVITKDETTIVDGAGDSEQVQGRVNQIRAEIENSDSDY     358
sp|Q18CT5|CH60_CLOD6                 ISEELGYDLKEADLSMLGRASSVKVTKESTTIVDGSGDKKAIEDRVTQIKHQVEQTTSDF     358
sp|B5XIW7|CH60_STRPZ                 ITEDLGLELKDATMTALGQAAKITVDKDSTVIVEGSGSSEAIANRIALIKSQLETTTSDF     358
sp|Q2FF95|CH60_STAA3                 ITDDLGLDLKDASIDMLGTASKVEVTKDNTTVVDGDGDENSIDARVSQLKSQIEETESDF     358
tr|A0A6M3Z7Y6|A0A6M3Z7Y6_BACSU       ITEDLGLDLKSTQIAQLGRASKVVVTKENTTIVEGAGETDKISARVTQIRAQVEETTSEF     358
sp|Q9AGE6|CH60_LISMO                 ITEDLGLELKTATVDQLGTANKVVVTKDDTTIVEGAGDSTQISARVNQIRAQMEETTSEF     358
                                     :::: . .*: . :  ** *  : : *: *.:::* *.   :  *:  :: ::  : *::

sp|P48220|CH60_ZYMMO                 DREKLQERVAKLAGGVAVIKVGGATEVEVKERKDRVDDALHATRAAVQEGIVPGGGTALL     420
tr|A0A0E8E5E7|A0A0E8E5E7_BORPT       DREKLQERVAKLAGGVAVIRVGAATEVEMKEKKARVEDALHATRAAVEEGVVPGGGVALL     420
sp|P0A6F5|CH60_ECOLI                 DREKLQERVAKLAGGVAVIKVGAATEVEMKEKKARVEDALHATRAAVEEGVVAGGGVALI     420
tr|A0A6N3JC79|A0A6N3JC79_FRATN       DREKLQERLAKLSGGVAVIKVGAVTEAEMKEKKDRVDDALHATRAAVEEGIVAGGGVALI     420
tr|L8EHR8|L8EHR8_STRR1               DREKLQERLAKLAGGVAVIKAGAATEVELKERKHRIEDAVRNAKAAVEEGIVAGGGVALL     418
sp|Q18CT5|CH60_CLOD6                 DREKLMERLAKLAGGVAVVKVGAATEVELKERKLRIEDALNATRAAVEEGIVAGGGTAFV     418
sp|B5XIW7|CH60_STRPZ                 DREKLQERLAKLAGGVAVIKVGAPTETALKEMKLRIEDALNATRAAVEEGIVAGGGTALI     418
sp|Q2FF95|CH60_STAA3                 DREKLQERLAKLAGGVAVIKVGAASETELKERKLRIEDALNSTRAAVEEGIVAGGGTALV     418
tr|A0A6M3Z7Y6|A0A6M3Z7Y6_BACSU       DREKLQERLAKLAGGVAVIKVGAATETELKERKLRIEDALNSTRAAVEEGIVSGGGTALV     418
sp|Q9AGE6|CH60_LISMO                 DREKLQERLAKLAGGVAVVKVGAATETELKERKLRIEDALNSTRAAVEEGIVAGGGTALV     418
                                     ***** **:***:*****::.*. :*. :** * *::**:. ::***:* ***.*::

sp|P48220|CH60_ZYMMO                 YATKTLEGLN-GVNEDQQRGIDIVRRALQAPVRQIAQNAGFDGAVVAGKLIDGNDDKIGF     479
tr|A0A0E8E5E7|A0A0E8E5E7_BORPT       RAKQAITGLK-GDTADQNAGIKLILRAVEEPLRTIVTNAGDEASVVVNTV-LNGKGNYGY     478
sp|P0A6F5|CH60_ECOLI                 RVASKLADLR-GQNEDQNVGIKVALRAMEAPLRQIVLNCGEEPSVVANTV-KGGDGNYGY     478
tr|A0A6N3JC79|A0A6N3JC79_FRATN       RAQKALDGLT-GENDDQNHGIALLRKAIEAPLRQIVSNAGGESSVVVNQV-KANQGNYGY     478
tr|L8EHR8|L8EHR8_STRR1               QASSVFEKL--ELEGDEATGAAAVKLALEAPLKQISVNGGLEGGVVVEKV-RNLAVGHGL     475
sp|Q18CT5|CH60_CLOD6                 SVIPAIGTLIESLEGEVKLGAQIVKKALEEPLRQIAINAGLEGAVIVQNV-VNSEAETGF     477
sp|B5XIW7|CH60_STRPZ                 TVIEKVAAL--ELEGDDATGRNIVLRALEEPVRQIALNAGYEGSVVIDKL-KNSPAGTGF     475
sp|Q2FF95|CH60_STAA3                 NVYQKVSEI--EAEGDIETGVNIVLKALTAPVRQIAENAGLEGSVIVERL-KNAEPGVGF     475
tr|A0A6M3Z7Y6|A0A6M3Z7Y6_BACSU       NVYNKVAAV--EAEGDAQTGINIVLRALEEPIRQIAHNAGLEGSVIVERL-KNEEIGVGF     475
sp|Q9AGE6|CH60_LISMO                 SIYNKVAAL--EAEGDVETGINIVLRSLEEPVRQIAHNAGLEGSVIVERL-KHEAVGVGF     475
                                          .  :       :   *       ::  *:: *  * * : .*:   :         *
```

```
sp|P48220|CH60_ZYMMO                NAQTEKYEDLAATGVIDPTKVVRTALQDAASVAGLLITTEAAVGDLPEDKPA--PAMPGG      537
tr|A0A0E8E5E7|A0A0E8E5E7_BORPT      NAATGEYGDLVEQGVLDPTKVTRTALQNAASVASLLLTAEAAVVELMENKPAAAPAMPGG      538
sp|P0A6F5|CH60_ECOLI                NAATEEYGNMIDMGILDPTKVTRSALQYAASVAGLMITTECMVTDLPKNDAA-DLGAAGG      537
tr|A0A6N3JC79|A0A6N3JC79_FRATN      NAANDTYGDMVEMGILDPTKVTRSALQHAASIAGLMITTEAMVGEIKEAAPA--MPMGGG      536
tr|L8EHR8|L8EHR8_STRR1              NAATGEYVDMIAEGIIDPAKVTRSALQNAASIAALFLTTEAVIADKPEKAAAA-------      528
sp|Q18CT5|CH60_CLOD6                DALNEKYVNMIEAGIVDPTKVSRSALQNAASIASTFLTTEAAVADLPEKEDAG-M--P--      532
sp|B5XIW7|CH60_STRPZ                NAATGEWVDMIKTGIIDPVKVTRSALQNAASVASLILTTEAVVANKPEPAAPA-PAMP-A      533
sp|Q2FF95|CH60_STAA3                NAATNEWVNMLEEGIVDPTKVTRSALQHAASVAAMFLTTEAVVASIPEKNNDQ-------      528
tr|A0A6M3Z7Y6|A0A6M3Z7Y6_BACSU      NAATGEWVNMIEKGIVDPTKVTRSALQNAASVAAMFLTTEAVVADKPEENGGG-AGMP-D      533
sp|Q9AGE6|CH60_LISMO                NAANGEWVNMIDAGIVDPTKVTRSALQNASSVAALLLTTEAVVADKPDENGP---AAV-P      531
                                    :* .  : ::   *::**.** *:*** *:*:*. ::*:*. : .  .

sp|P48220|CH60_ZYMMO                MGGMGGMDF----- 546
tr|A0A0E8E5E7|A0A0E8E5E7_BORPT      MGGMGGMDF----- 547
sp|P0A6F5|CH60_ECOLI                MGGMGGMGGMM--- 548
tr|A0A6N3JC79|A0A6N3JC79_FRATN      MGG---MPGMM--- 544
tr|L8EHR8|L8EHR8_STRR1              -GAPGGMPGGDMDF 541
sp|Q18CT5|CH60_CLOD6                -GMGGGMPGMM--- 542
sp|B5XIW7|CH60_STRPZ                GMDPGMMGGF---- 543
sp|Q2FF95|CH60_STAA3                -PNMGGMPGMM--- 538
tr|A0A6M3Z7Y6|A0A6M3Z7Y6_BACSU      MGGMGGMGGMM--- 544
sp|Q9AGE6|CH60_LISMO                DMGMGGMGGMM--- 542
                                        *
```

Figure S16. Protein sequence alignment of 60 kDa chaperonin (chaperonin GroEL, GroEL) indicates conserved pgK sites. PgK sites that have been identified in *Z. mobilis* (ZYMMO) [48], *B. pertussis* (BORPT) [49], *E. coli* (ECOLI) [46], *F. novicida* (FRATN) [47], *S. rimosus* (STRR1) [45], *C. difficile* (CLOD6) [44], *S. pyogenes* M49 (STRPZ) [this work], *S. aureus* (STAA3) [39], *B. subtilis* (BACSU) [38], and *L. monocytogenes* (LISMO) [35] are highlighted in red.

```
tr|A0A0H2ZMW4|A0A0H2ZMW4_STRP2      MANKQDLIAKVAEATELTKKDSAAAVEAVFAAVADYLAAGEKVQLIGFGNFEVRERAERK      60
sp|P0C0H3|DBH_STRP1                 MANKQDLIAKVAEATELTKKDSAAAVDAVFSTIEAFLAEGEKVQLIGFGNFEVRERAARK      60
tr|A0A0H3C0P8|A0A0H3C0P8_STRPZ      MANKQDLIAKVAEATELTKKDSAAAVDAVFSTIEAFLAEGEKVQLIGFGNFEVRERAARK      60
tr|Q92A74|Q92A74_LISMO              MANKTDLVNSVAELADLSKKDAAKAVEAVFETIQTSLSKGEKVQLIGFGNFEVRERAARK      60
tr|A0A0H2XF50|A0A0H2XF50_STAA3      -MNKTDLINAVAEQADLTKKEAGSAVDAVFESIQNSLAKGEKVQLIGFGNFEVRERAARK      59
tr|A0Q6S6|A0Q6S6_FRATN              -MNKSELVSAIAKEADVTKEVASNTLDATIAAVTKALKNGDSVTLVGFGTFQVKERSPRE      59
sp|P0ACF0|DBHA_ECOLI                -MNKTQLIDVIAEKAELSKTQAKAALESTLAAITESLKEGDAVQLVGFGTFKVNHRAERT      59
tr|A0A0T7CR47|A0A0T7CR47_BORP1      -MNKTELIDHIASKADISKAAAGRSLDALIGAVKTTLKKGGTVTLVGFGTFAVSARAART      59
sp|P0ACF4|DBHB_ECOLI                -MNKSQLIDKIAAGADISKAAAGRALDAIIASVTESLKEGDDVALVGFGTFAVKERAART      59
                                      ** :*:  :*  ::::*  :  :::: : ::   *  *  * *:***.* *  *: *

tr|A0A0H2ZMW4|A0A0H2ZMW4_STRP2      GRNPQTGKEMTIAASKVPAFKAGKALKDAVK          91
sp|P0C0H3|DBH_STRP1                 GRNPQTGAEIEIAASKVPAFKAGKALKDAVK          91
tr|A0A0H3C0P8|A0A0H3C0P8_STRPZ      GRNPQTGAEIEIAASKVPAFKAGKALKDAVK          91
tr|Q92A74|Q92A74_LISMO              GRNPRTKEEIDIPASKVPAFKPGKALKEAVK          91
tr|A0A0H2XF50|A0A0H2XF50_STAA3      GRNPQTGKEIDIPASKVPAFKAGKALKDAVK          90
tr|A0Q6S6|A0Q6S6_FRATN              GRNPKTGETIKIPASKVPSFKAGKGLKDAVK          90
sp|P0ACF0|DBHA_ECOLI                GRNPQTGKEIKIAAANVPAFVSGKALKDAVK          90
tr|A0A0T7CR47|A0A0T7CR47_BORP1      GRNPRTGETIKIKKAKVPKFRPGKALKDAVN          90
sp|P0ACF4|DBHB_ECOLI                GRNPQTGKEITIAAAKVPSFRAGKALKDAVN          90
                                    ****:*   : *  ::** *  **.**:**:
```

Figure S17. Protein sequence alignment of DNA-binding protein Hu indicates conserved pgK sites. PgK sites that have been identified in *S. pneumoniae* (STRP2) [37], *S. pyogenes* M1 (STRP1) [35], *S. pyogenes* M49 (STRPZ) [this work], *L. monocytogenes* (LISMO) [35], *S. aureus* (STAA3) [39], *F. novicida* (FRATN) [47], *E. coli* (ECOLI) [46], *and B. pertussis* (BORPT) [49] and are highlighted in red.

```
sp|P0ABS1|DKSA_ECOLI                ---------MQEGQNRKTSSLSILAIAGVEPYQEKPGEEYMNEAQLAHFRRILEAWRNQL      51
tr|A0A0E7USP1|A0A0E7USP1_BORPT      MA-----TKAATKKSSKSTSDTAIDLPSEQELLAMPEADYMNDRQLAFFKERLKQLEQDI      55
tr|Q5NNU9|Q5NNU9_ZYMMO              MASNFTASKVDTNGIKTSALEKAPSVAAPDDYRPSPDEPFMNERQQAYFREKLLKWKEAI      60
                                                   .::  .   : . :     *   :**: * *.*:. *   .: :

sp|P0ABS1|DKSA_ECOLI                RDEVDRTVTHMQDEAANFPDPVDRAAQEEEFSLELRNRDRERKLIKKIEKTLKKVEDEDF      111
tr|A0A0E7USP1|A0A0E7USP1_BORPT      LANAGETTEHLRETQ-FVPDPADRATIEEEHALELRTRDRERKLLKKVQQAIARIDSGEY      114
tr|Q5NNU9|Q5NNU9_ZYMMO              LIESRGTLAQLRQESLKEADLTDRASSETDWSIELRTRDRQRKLISKIDAALRRLDEGEY      120
                                      :   *  ::::      * .***: * : ::***.***:***:.*:: :: :::. ::

sp|P0ABS1|DKSA_ECOLI                GYCESCGVEIGIRRLEARPTADLCIDCKTLAEIREKQMAG-            151
tr|A0A0E7USP1|A0A0E7USP1_BORPT      GWCEETGEPIGVPRLLARPTATLSLEAQERREMRQKLYGD-            154
tr|Q5NNU9|Q5NNU9_ZYMMO              GYCEVTGEPISLARLEARPIATMTIEAQEKHERHEKVSREE            161
                                    *:**  *  *.: ** *** * : ::.:   * ::*
```

Figure S18. Protein sequence alignment of RNA polymerase-binding transcription factor DksA indicates conserved pgK sites. PgK sites that have been identified in *E. coli* (ECOLI) [46], *B. pertussis* (BORPT) [49], and *Z. mobilis* (ZYMMO) [48] are highlighted in red.
