# Supplementary figures and images for: Lysine Phoshoglycerylation Is Widespread in Bacteria and Overlaps with Acylation
Source: Microorganisms. 2024 Jul 30;12(8):1556. doi: 10.3390/microorganisms12081556 (PMC11356508; doi:10.3390/microorganisms12081556)

## Experiment 1

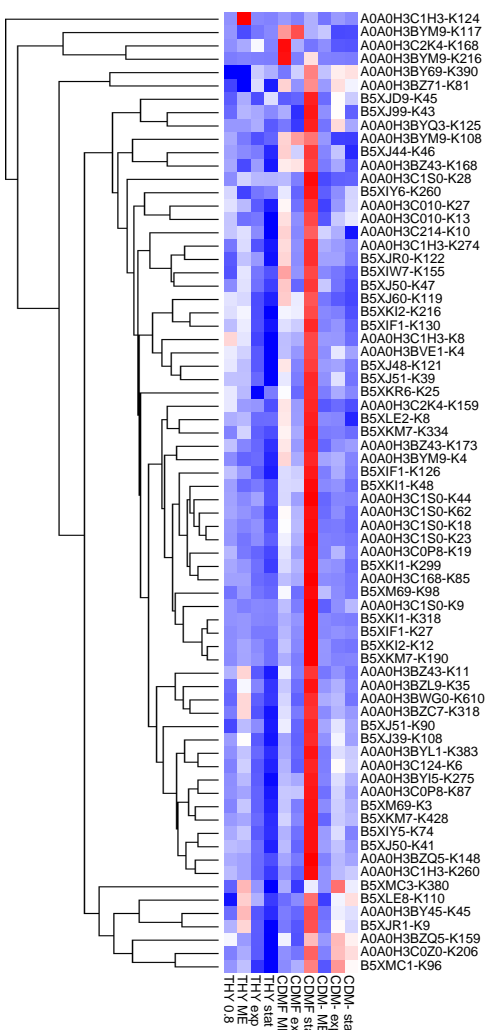

### Data Distribution

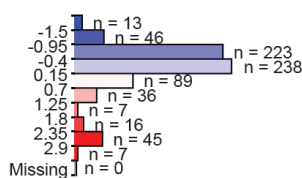



Experiment 3

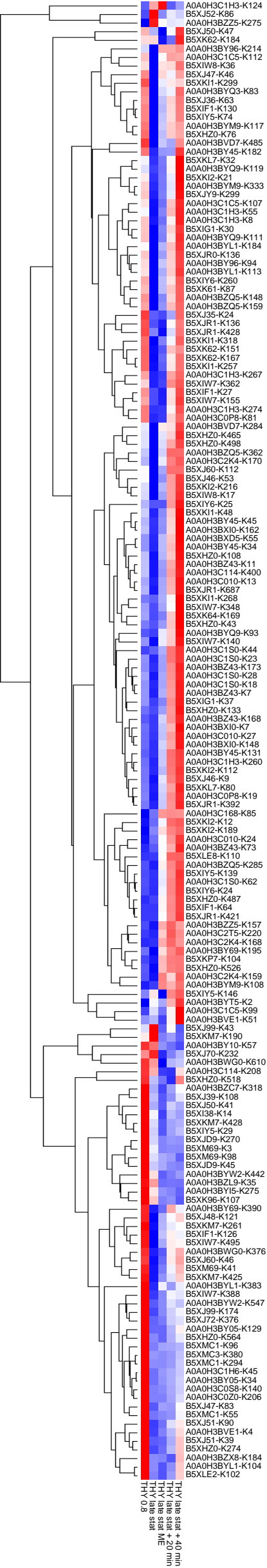

Data Distribution

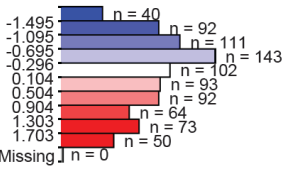

Supplement: Supplementary file 1 [file microorganisms-12-01556-s001.zip › Supplementary Data Sheet S1.pdf]
